# The Type Specimens of *Plectocolea* and *Solenostoma* (Marchantiophyta) in Some Japanese and European Herbaria

**DOI:** 10.3390/plants12233935

**Published:** 2023-11-22

**Authors:** Vadim A. Bakalin, Seung Se Choi

**Affiliations:** 1 Laboratory of Cryptogamic Biota, Botanical Garden-Institute FEB RAS, Makovskogo Street 142, Vladivostok 690024, Russia; 2 Team of National Ecosystem Survey, National Institute of Ecology, Seocheon 33657, Republic of Korea

**Keywords:** Marchantiophyta, Solenostomataceae, *Solenostoma*, *Plectocolea*, Pacific Asia, East Asia, type specimens, authentic materials

## Abstract

Solenostomataceae are among the most diverse and, at the same time, least described liverwort families in Pacific Asia. Many species therein are known only from type descriptions, which are sometimes incomplete. The present account is based on a study of 81 type specimens belonging to 61 species known mostly in East Asia, although also in other regions; it provides line-art figures and photomicrographs of all the studied taxa. One lectotype and one epitype are designated, and six new combinations are proposed. as Additionally, the indication of a necessity of an epitype for *Plectocolea setulosa* Herzog is justified.

## 1. Introduction

This paper continues the work that was begun in 2014 (Bakalin, 2014 [1]), when the first author prepared a conspectus of type specimens of Solenostomataceae stored in the herbarium of the Conservatoire et Jardin Botanique de la Ville de Genève (international acronym G). That study provided descriptions and figures of the types of taxa mostly described by the famous German hepaticologist F. Stephani (1842–1927). The reasons that inspired us to continue this study are the same as in the previous case: the occasional inappropriate shortness of available descriptions, incompleteness (or absence) of illustrations, the partial discrepancies between illustrations and descriptions based on the morphology of type specimens, and differentiation between the features of type specimens with subsequent interpretations by various writers. The present study contains descriptions and illustrations of 81 type specimens of 61 species stored in the herbaria BM, G, HIRO, JE, NICH, PC and STR.

## 2. Results

A total of 81 type specimens belonging to 61 species were studied, including 71 holotypes or isotypes, 1 paratype, 1 syntype, 1 epitype and 7 lectotypes or isolectotypes. Line-art figures and grayscale photographs were compiled for all studied taxa. The lectotype for *Nardia granulata* Steph. is selected. The epitype for *Plectocolea setulosa* Herzog is proposed. Six new combinations are proposed (*Plectocolea tuberculifera* (Herzog) Bakalin et S.S. Choi comb. nov., *Plectocolea borneensis* (Amakawa) Bakalin et S.S. Choi comb. nov., *Plectocolea champawatensis* (S.N. Srivast. et Amakawa) Bakalin et S.S. Choi comb. nov., *Plectocolea flavialbicans* (Amakawa et Grolle) Bakalin et S.S. Choi comb. nov., *Plectocolea pfleidereri* (Amakawa & Váňa) Bakalin et S.S. Choi comb. nov., and *Plectocolea plagiochilacea* (Grolle) Bakalin et S.S. Choi comb. nov.). We propose the elevation of one variety to the species rank, and a new name is proposed (*Solenostoma amakawanum* Bakalin et S.S. Choi, nom. nov. pro *Jungermannia pyriflora* var. *gracillima* Amakawa).

## 3. Discussion

The total taxonomic diversity of the genera *Solenostoma*, *Metasolenostoma*, *Protosolenostoma*, and *Plectocolea* (=*Solenostoma* s.l.) in the world is difficult to estimate. The main pool of studied specimens comes from the Pacific Asia, where there may be slightly over 150 taxa. Of these, sufficiently detailed descriptions are available for approximately 100 species. The situation in other macro-regions of Earth has been better evaluated due to the publications of Váňa [2,3,4], etc. There are no summary treatments for *Solenostoma* s.l. in Pacific Asia, although there are incomplete and currently out-of-date treatments by Amakawa for the Sino-Himalayas and Southeast Asia [5,6,7], etc., Japan [8], China [9], the Russian Far East [10], and Korea [11]. Only in recent years has more or less complete information on the oil bodies of about 50% of *Solenostoma* species found outside Japan (Russian Far East, Southern China, Vietnam, and the Korean Peninsula) been obtained [12]. The preliminary results of studies on the morphology of species from these areas show that morphologically new species should be described. Even a relatively small and preliminary study from South Korea, which was generally recognized as producing only an incomplete list compared to the Japanese study, provided two species new to science [11].

Most of the taxa treated here have type localities in the Sino-Himalaya; these are 21 species. The next most come from the ‘macro-region’ of Japan plus Taiwan (17 species, including 15 from Japan and 2 from Taiwan). Malesia is represented by 8 species (6 from Indonesia and 2 from Malaysia). Melanesia is represented by five species, the same number as from Europe. Information on other regions is rather of an additional nature, and is therefore represented by a small number of species: Africa—one, Asia Minor—one, South America—one, and North America—two. To a large extent, this distribution reflects both the center of diversity of the family in the Eastern Hemisphere and the scope of our interests. The same distribution suggests a clear under-exploration of northern Indochina, since it is unlikely that the relative richness of the Sino-Himalaya and Malesia regions are separated by a ‘gap’ in Indochina, despite the presence of fairly high mountains there.

## 4. Taxonomic Treatment

*Alicularia haskarliana* Nees, Syn. Hepat. 1: 12, 1844.

Type: Indonesia, Java insula ad terram, denso caespite; inter alios muscos inventa; Haskarl (Holotypus STR, s.n.!).

Accepted name: *Plectocolea haskarliana* (Nees) Mitt. Flora Vitiensis 405. 1871. (=*Solenostoma haskarlianum* (Nees) R.M.Schust. ex Váňa et D.G.Long, Nova Hedwigia 89 (3/4): 502, 2009).

**Figure 1:**
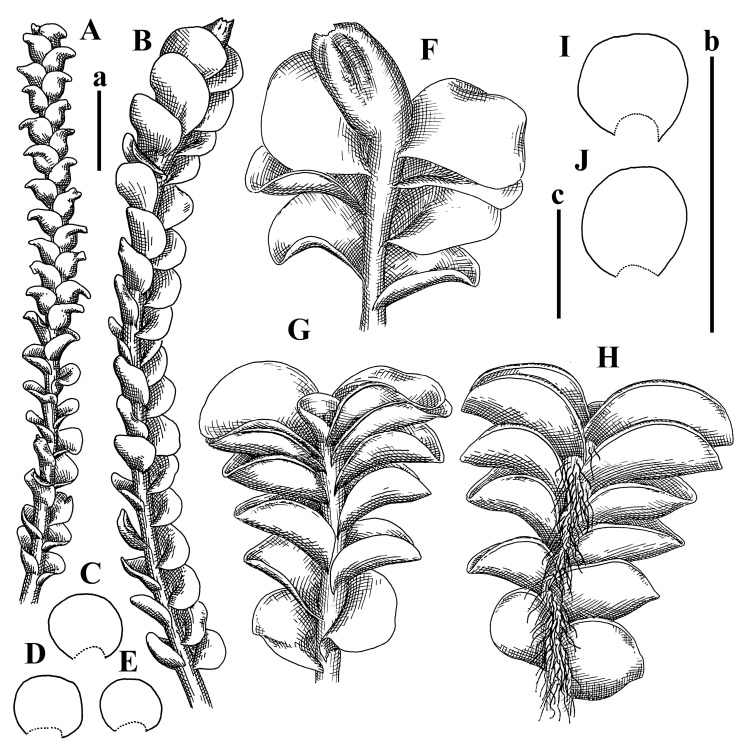
*Alicularia haskarliana* Nees Holotypus, STR, s.n.: (**A**) male plant; (**B**) perianthous plant; (**C**–**E**) leaves. *Aplozia stephanii* Schiffn. Lectotypus, G00120794: (**F**) perianthous plant; (**G**) shoot, dorsal view; (**H**) shoot, ventral view; (**I**,**J**) leaves. Scales: a—1 mm for (**A**–**E**); b—5 mm for (**F**–**H**); c—2 mm for (**I**,**J**).

**Figure 2:**
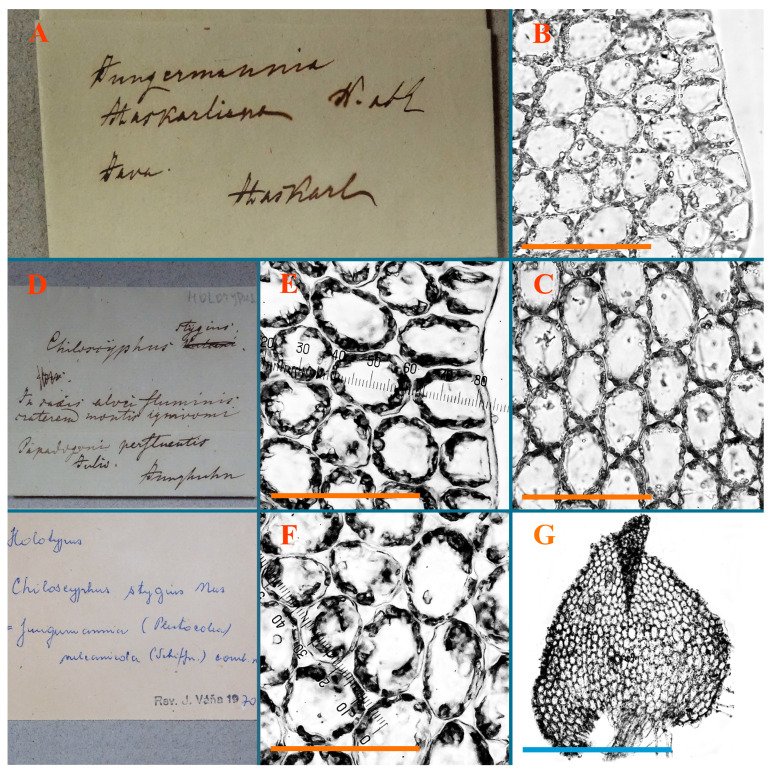
*Alicularia haskarliana* Nees Holotypus, STR, s.n.: (**A**) holotype label. *Aplozia stephanii* Schiffn. Lectotypus, G00120794: (**B**) cells leaf margin; (**C**) midleaf cells; *Chiloscyphus stygius* Nees STR, s.n.: (**D**) holotype label. *Eucalyx boninensis* Horik. Holotypus, HIRO, s.n.: (**E**) leaf margin cells; (**F**) midleaf cells. *Haplozia tuberculifera* Herzog, Holotypus, JE–04009127: (**G**) leaf. Scales: 100 µm for (**B**,**C**,**E**,**F**); 1 mm for (**G**).

Description: Plants erect, in dense patches, brownish purple to (rarer) yellowish brownish, 1000 (male) to 1750 (female) µm wide, 15–20 mm long. Rhizoids numerous, brownish, decurrent in very distinct fascicle along dorsal side of the stem. Stem branching not seen (even as subfloral innovations). Leaves obliquely inserted, obliquely spreading and obliquely oriented, decurrent for 2/2–3/2 of stem width in both sides, sheathing the stem in the base, above concave–canaliculate, transversely elliptic, 675–750 × 875–1000 µm, loosely undulate along margin, contiguous to imbricate, leaf lamina strongly rhizogenous, rhizoids start only 2 cells inward of leaf margin. Midleaf cells 37.5–50.0 × 25.0–37.5 µm, thin-walled, trigones moderate to large, bulging, cuticle nearly smooth; cells along margin 17.5–27.5 µm, merely thick-walled, with external wall strongly thickened, trigones large, bulging. Dioicous. Androecia intercalary, with 10–20 pairs of bracts, 2–3-androus, making bracts inflate in lower half, and recurved along margin. Perianth conical, pluriplicate, exerted for 1/2 of the length; perigynium ca. 2 perianth lengths; bracts obliquely spreading or sheathing the perianth (Figure 1A–E and Figure 2A).

Comment: There are several specimens of *Alicularia haskarliana* in STR. One of them was marked by Váňa as Holotype; however, the specimen he indicated does not have any other handwriting marks (even indicating that the specimen is collected in Java). In our opinion, other specimens should be regarded as Holotype, where there is distinct mark collected in Java by Haskarl. Moreover, it is not necessary to create lectotypification in this case, because there is no evidence that other specimens were collected by Haskarl et al.

Plants have dense rhizoid fascicles decurrent down along dorsal side of stem, which helps to recognize the species easily. Some difficulties may occur in the distinctions between *P. haskarliana* and *P. ariadne* (Taylor) Mitt., due to the unstable height of the perigynium and overlapping cell measures (commonly treated as the distinction features). Easy distinction is possible in the living condition, when it is possible to observe oil bodies granulated in *P. hasskarliana* and very finely granulated in *P. ariadne*, as noted in [12].

*Aplozia stephanii* Schiffn., Denkschr. Kaiserl. Akad. Wiss., Math.-Naturwiss. Kl. 67: 195, 1898.

Type: Indonesia, Java, in monte Pangerango, region alpina, ad terram; 9 May 1894, V. Schiffner 594 (lectotypus designated by Bakalin [1] G00120794!; isolectotypus PC0103855!).

Accepted name: *Solenostoma stephanii* (Schiffn.) Steph., Bull. Herb. Boissier (sér. 2) 1 (5): 496 (58), 1901.

A description based on plants in lectotype was published by Bakalin [1]; additional illustrations are here (Figure 1F–J and Figure 2B,C).

Comment: (1) Leaf margin is not constantly revolute (which is not so characteristic, and similar to that observed in *Jungermannia mieheana* Steph., regarded as a synonym of *Solenostoma bauerii* (Schiffn.) Steph., [13], cf. also [1], this feature is more obvious in the ventral side of the shoot. The species is characterized by very large plants, 5–5.5 mm wide; rhizoids dense, brownish, originated near ventral leaf base, but also from other parts of ventral side of the stem; leaves suborbicular to transversely elliptic, 1750–3000 × 2300–3250 µm; midleaf cells 32.0–62.5 × 30.0–37.5 µm, thin-walled, trigones moderate in size, convex; cells along margin 20.0–37.5 µm, thin-walled, with external wall thickened, trigones moderate in size, convex.

(2) Another specimen: PC-0103856! (V. Schiffner 520; Prov. Praenger, in regione superior montis ignivomi Gedeh, ad terram infra craterem, region alpina, 2700 m a.s.l. 10 July 1894) is the syntype of the taxon and represent the small form of the species, with plants 2–3 mm wide, with margin revolute in ventral side only (and in any way near shoot apices only).

*Chiloscyphus stygius* Nees, Synopsis Hepaticarum 189. 1845.

Type: Indonesia. Java, in saxis alvei fluminis craterem montic ignivomi Papndayang perfluentis; Julio, Junghuhn, s.n. (Holotype STR!)

Accepted name: *Plectocolea stygia* (Nees) Bakalin et S.S. Choi comb. nov. Basionym *Chiloscyphus stygius* Nees, Synopsis Hepaticarum 189. 1845.

=*Nardia vulcanicola* Schiffn., Denkschr. Kaiserl. Akad. Wiss., Math.-Naturwiss. Kl. 67: 191, 1898 (=*Solenostoma vulcanicola* (Schiffn.) Nyushko ex Potemkin et Sofronova, Liverworts and Hornworts of Russia 1: 289. 2009; *Plectocolea vulcanicola* (Schiffn.) Bakalin Arctoa 23: 117. 2014).

**Figure 3:**
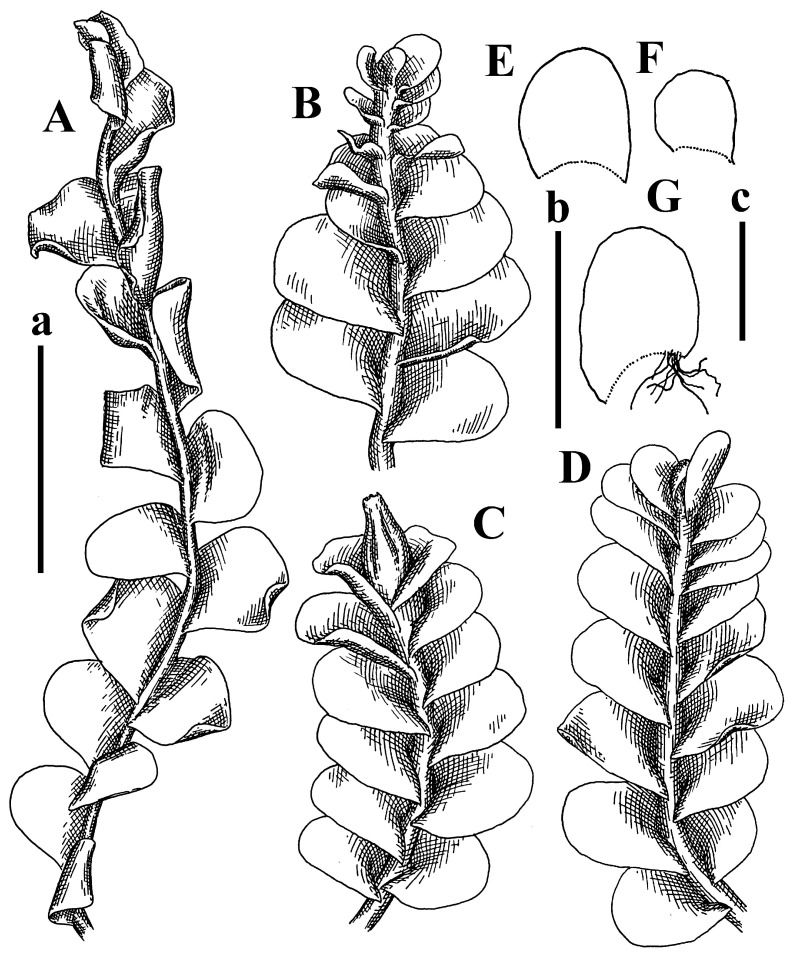
*Chiloscyphus stygius* Nees Holotypus. STR, s.n.: (**A**) plant habit. *Eucalyx boninensis* Horik. Holotypus, HIRO, s.n.: (**B**,**D**) plant habit; (**C**) perianthous plant; (**E**–**G**) leaves. Scales: a—3 mm for (**A**); b—2 mm for (**B**–**D**); c—1 mm for (**E**–**G**).

Description (plants very badly preserved due to originally lax texture): Plants soft, lax, gentle, encrusted by rusty orange-colored fine ground (similar to travertine), prostrate, in loose mats, 1500–3000 µm wide and 20–30 mm long, whitish to blackish in color. Rhizoids common, soft, flexuous, nearly colorless, obliquely spreading, forming loose mat under stem. Leaves subhorizontally inserted, transversely elliptic lingulate, shortly decurrent dorsally, undulate at the margin, 1125–1500 × 1250–1500 µm. Midleaf cells (mostly strongly collapsed, that features are unclear), ca. 45–55 × 20–35 µm, thin-walled, trigones small to indistinct, concave (Figure 2D and Figure 3A).

Comment: This taxon was regarded as the synonym of *Plectocolea vulcanicola* starting from [13], but despite obvious priority, no combinations with *Chiloscyphus stygius* were provided. Both types (*Nardia vulcanicola* and *Chiloscyphus stygius*) were collected in virtually the same habitat (although in different volcanoes), which additionally confirms the identity of both names.

*Eucalyx boninensis* Horik., Journal of Science of the Hiroshima University, Series B, Division 2 (Botany) 2: 142. f. 10. 1934.

Type: Japan, Bonin Island, Chichijima, Mt. Chuosan; 27 July 1930 Y. Horikawa 1940; (Holotypus HIRO, s.n.!)

Accepted name: *Plectocolea boninensis* (Horik.) S. Hatt., Bulletin of the Tokyo Science Museum 11: 37. 1944.

Description: Plants prostrate, pallid, yellowish to yellowish brownish, dorsiventrally flattened, 2–3 mm wide and 10–20 mm long, forming loose mats on fine soil. Stem 150–300 µm in diameter, slightly flexuous, sparsely laterally branched as subfloral innovations only. Rhizoids sparse to (rarely) dense, colorless to brownish and pale purplish to pinkish, originated mostly near ventral leaf base, but not only there, obliquely to erect spreading, separated or united into unclear fascicles or forming loose mat under stem. Leaves contiguous, flattened to slightly convex (giving a sometimes ‘chiloscyphoid’ appearance), very obliquely to subhorizontally inserted and oriented, 950–1500 × 900–1500 µm, nearly lingulate, not or barely decurrent dorsally, ventral end of insertion line subtransverse, not decurrent ventrally. Midleaf cells 40–60 × 30–50 µm, thin-walled, trigones moderate in size, convex, cuticle smooth; along margin 30–40 µm, thin-walled or walls slightly and unequally thickened in radial wall due to trigones’ confluence, trigones moderate in size to (rarer) large, convex, external wall thickened, cuticle smooth or nearly so. Dioicous. Androecia terminal, narrower than sterile branch, with 3–6 pairs of bracts, 2–4-androus, male bracts strongly inflated in lower half and obliquely spreading and slightly concave–canaliculate above. Perianth conical, 1500–2000 × 600–1000 µm, pluriplicate, with 3–4 main plicae, exerted for 2/3–3/4 of its length or more, composed of elongated cells, unistratose to base; perigynium 1/5–1/4 of perianth length, with one pair of bracts; bracts similar to leaves, but slightly wider, sheathing perianth in lower 1/4 and obliquely spreading above, and commonly with widely recurved margin (Figure 2E,F and Figure 3B–G).

Comment: The species was synonymized by Váňa and Inoue [14] with very broadly treated *Plectocolea truncata* (Nees) Herzog. Here, we treat it as the species distinct from *P. truncata,* following the work of Váňa [3]. Indeed, this taxon is characterized, in comparison with *P. truncata* s. str., by larger leaf cells, the absence of a leaf border formed by cells with thickened walls (here, only the external wall is thickened), and especially subhorizontally oriented and slightly convex leaves that gives the appearance of *Chiloscyphus*.

*Haplozia tuberculifera* Herzog, Ann. Bryol. 5: 84, 1932.

Type: Ost-Borneo: An einem Baumstamm; Tandjoeng Redet, Bez. Beran; 1913 R. Wegner (Holotypus JE04009127!).

Accepted name: *Plectocolea tuberculifera* (Herzog) Bakalin et S.S. Choi comb. nov. Basionym: *Haplozia tuberculifera* Herzog, Ann. Bryol. 5: 84, 1932. (=*Solenostoma tuberculiferum* (Herzog) Váňa, Hentschel et Heinrichs, Cryptog. Bryol. 31 (2): 138, 2010).

**Figure 4:**
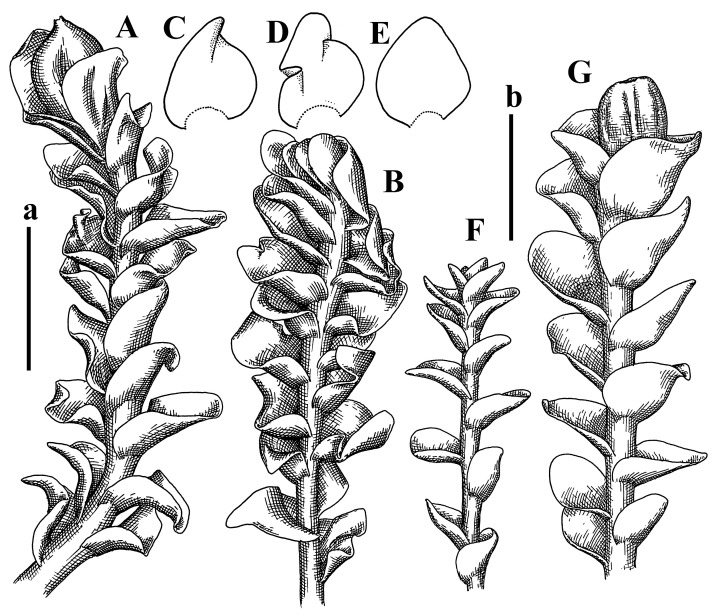
*Haplozia tuberculifera* Herzog, Holotypus, JE–04009127: (**A**) perianthous plant; (**B**) plant habit; (**C**–**E**) leaves. *Jungermannia abyssinica* Nees, Holotypus, STR, s.n.: (**F**) plant habit; (**G**) perianthous plant. Scales: a—2 mm for (**A**–**E**); b—2 mm for (**F**,**G**).

**Figure 5:**
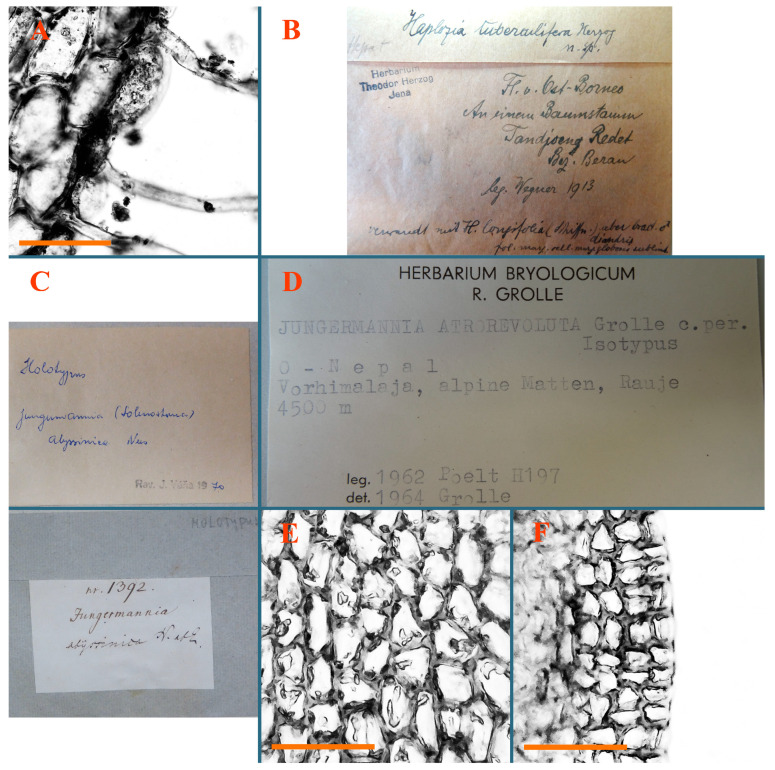
*Haplozia tuberculifera* Herzog, Holotypus, JE–04009127: (**A**) leaf margin cells; (**B**) holotype label). *Jungermannia abyssinica* Nees, Holotypus, STR, s.n.: (**C**) holotype label. *Jungermannia atrorevoluta* Grolle ex Amakawa, Isotypus, HIRO, s.n.: (**D**) isotype label; (**E**) midleaf cells; (**F**) leaf margin cells. Scales: 100 µm for (**A**); 50 µm for (**E**,**F**).

Description: Plants grayish dirty green to deep grayish, merely soft, probably prostrate to ascending, well-developed shoots 2–3 mm wide and 20–30 mm long (probably longer in the nature). Stem branching not seen (we also did not see ventral innovations with ‘tubercles’). Rhizoids numerous, grayish to brownish, forming loose and thick but distinct fascicle going down the stem, or rhizoids obliquely spreading; no rhizoid fascicle developed, originating from ventral side of the stem or from margin of ventral leaf base. Leaves obliquely inserted, dorsally decurrent for 1/2–2/2 of stem width, obliquely oriented and spreading, concave–canaliculate, undulate not only along margin (due to high softness), ovate triangular when flattened in the slide, 1.5–1.6 × 1.4–1.5 mm, margin distinctly crenulate. Midleaf cells very large, thin-walled, trigones small, but distinct, concave, 75–100 × 40–65 µm, cuticle smooth; cells along margin 50–90 µm, thin-walled, with thin external wall too; on ventral side near base, marginal cells distinctly rhizogenous. Dioicous. Androecia tentatively intercalary with 8–10 and more pairs of bracts (we did not see the distance between sterile leaves of different generations), monandrous; male bracts strongly ventricose in lower half and erect spreading above. Figure 2G, Figure 4A–E and Figure 5A,B.

Comment: The species is characterized by very large leaf cells, a crenulate leaf margin, peculiarly rhizogenous ventral leaf margin base, and dense rhizoids.

*Jungermannia abyssinica* Nees, Synopsis Hepaticarum 93. 1844.

Type: Abyssinia. In montis Silke, supra regionem fruticum ad rivules et rupes umbroses; 22.II.1840. W.P. Schimper (s.n.) (Holotypus STR, s.n.!)

Accepted name: *Solenostoma abyssinicum* (Nees) Steph., Sp. Hepat. 2: 53. 1901.

Comment: This species was previously illustrated based on the isotype in G [1] (Figure 4F,G and Figure 5C). Plants in the holotype are nearly identical to the isotype. The description of androecia should be added: androecia in 1–2 pairs below female bracts, 1–2-androus, antheridia easily removable; male bracts very loosely inflated in lower half; pure male branches rarely occur (possibility of protheoandry). Similar to *S. spherocarpum* (Hook.) Steph. and *S. confertissimum* (Nees) Schljakov, from which it may be differentiated using the following Table 1.

The original source [15] cites number 1392 that is absent in isotype in G, but present in herb STR. We are not sure the plants in the ‘isotype’ in G really originate from the divided holotype.

The species was regarded as synonymous with *S. confertissimum* in Shaw et al. (2015 [16]) due to of the unity of a specimen from Kenya in the same clade with the specimen named as *S. confertissimum* from Austria, although an adjacent clade contains all other specimens of *S. confertissimum*. However, we think this taxon may be recognized, and the concept of *S. confertissimum* probably should be clarified; as a consequence, the circumscription of ‘*S. confertissimum*’ may be clarified too.

*Jungermannia atrorevoluta* Grolle ex Amakawa, J. Hattori Bot. Lab. 29: 255, 1966.

Type: Ost-Nepal, Vorhimalaja, Alpine Matten, Rauje, 4500 m alt.; 1962 Poelt H197 (Holotypus JE, s.n.!, Isotypus HIRO, s.n.!).

Accepted name: *Solenostoma atrorevolutum* (Grolle ex Amakawa) Váňa et D.G.Long, Nova Hedwigia 89 (3/4): 496, 2009.

**Figure 6:**
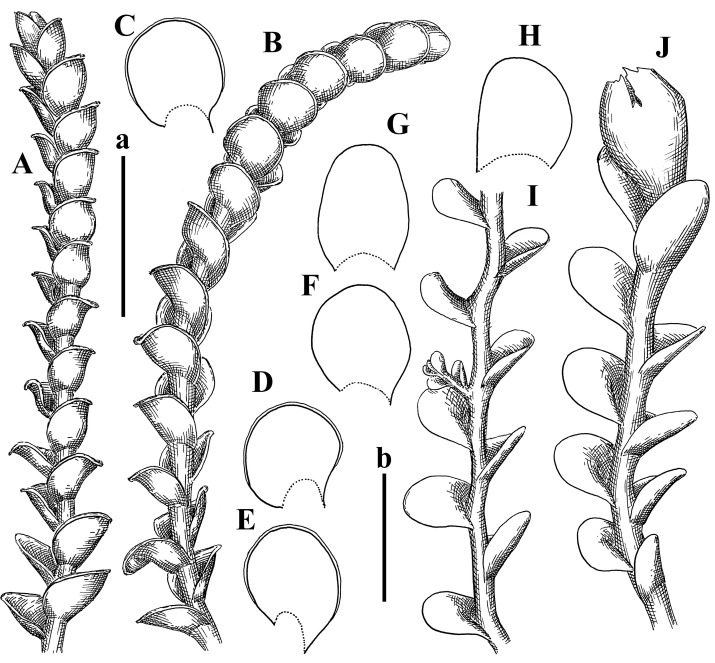
*Jungermannia atrorevoluta* Grolle ex Amakawa, Isotypus, HIRO, s.n.: (**A**,**B**) plant habit; (**C**–**E**) leaves, abaxial view. *Jungermannia bengalensis* Amakawa Isotypus, HIRO, s.n.: (**F**–**H**) leaves; (**I**) plant habit fragment; (**J**) perianthous plant. Scales: a—1 mm for (**A**–**E**); b—1 mm for (**F**–**J**).

Description: Plants 0.6–1.0 mm wide, brown to yellowish brownish, more or less rigid, erect, forming loose patches. Stem 70–120 µm in diameter, brown, sparsely laterally branched. Rhizoids numerous, forming distinct fascicle and decurrent down the stem, the fascicle diameter 1.5–2.0 times larger than the stem diameter, originated from the stem. Leaves transversely inserted, dorsally decurrent for 1–2(3) stem widths and for 2–4 stem widths decurrent ventrally, imbricate and closely sheathing the stem and lower half of leaf above, narrowly revolute along margin, concave–canaliculate to canaliculate and spoon-shaped (giving appearance of Marsupella revoluta), 500–600 × 600–700 µm, nearly orbicular. Midleaf cells oblong, 14–20 × 10–15 µm, thin-walled, trigones moderate in size, triangle to slightly convex, cuticle smooth; cells along leaf margin thin-walled to unequally thickened due to trigones’ confluence, 7–15 µm; external wall slightly thickened to nearly thin, cuticle smooth (Figure 5D–F and Figure 6A–E).

Comment: Brown color, narrowly revolute leaf margin, and rhizoids in distinct thick fascicle. It is worth mentioning that the specimen in JE is marked as isotypus, the same as the specimen in HIRO. Since the species was described by Grolle (on the authority of Amakawa [6]), we think the specimen in JE should be regarded as a holotype.

*Jungermannia bengalensis* Amakawa, J. Hattori Bot. Lab. 31: 112, 1968 (Amakawa 1968b). (=*Jungermannia filamentosa* Amakawa Journal of the Hattori Botanical Laboratory 30: 194. f. 15: a–k. 1967. illegitimate, later homonym *Jungermannia filamentosa* Lehm. & Lindenb. Novarum et Minus Cognitarum Stirpium Pugillus 6: 29–30. 1834).

Type: India, West Bengal, near Sandakphu, Darjeeling area, 11,900–11,600 ft. alt. [3627–3535 m alt.], on wet rock; 26 April 1965 Z. Iwatsuki and A.J. Sharp (Holotypus NICH262349!; Isotypus HIRO, s.n.!)

Accepted name: *Solenostoma bengalense* (Amakawa) Váňa et D.G. Long, Nova Hedwigia 89 (3/4): 496, 2009.

Description: Plants ascending in loose to dense mats, brownish to yellowish brownish, without traces of red pigmentation, 0.7–1.5 mm wide and 8–15 mm long. Stem 150–220 µm in diameter, sparsely laterally branched, with commonly new normally developed branches originating from rhizomatous base (looks similar to geotropic stolon). Rhizoids sparse (dense near branches’ origins), obliquely spreading, commonly united into unclear fascicles. Leaves distant, obliquely to erect spreading, slightly canaliculate or loosely sheathing stem near base and then convex above, 600–1000 × 550–1100 µm, nearly ovate. Midleaf cells oblong, 30–70 × 20–35 µm, thin- to slightly thick-walled, trigones vestigial, cuticle virtually smooth; cells along leaf margin 20–40 µm, thin-walled to obscurely thick-walled, trigones vestigial. Possibly dioicous. Perianth obovate, exerted for the whole length, loosely 3–4-plicate in upper 1/5–1/4 of its length, suddenly contracted to a very shortly beaked mouth, 1300–1800 × 900–1000 µm, bistratose in lower half; perigynium virtually absent; bracts similar to large leaves, erect and spreading (Figure 6F–J).

Comment: The species is similar to *Solenostoma minutissimum* (Amakawa) Bakalin, Vilnet & Furuki, from which differs in leaf shape, with larger leaf cells, the absence of red pigmentation, and perianth shape (obovate and suddenly shortly beaked versus fusiform, gradually narrowing to a wide and conical beak in *S. minutissimum*).

*Jungermannia borneensis* Amakawa, J. Hattori Bot. Lab. 33: 160, 1970.

Type: North Borneo (Malaysia), between Kambaranga Radio Station and waterfalls, Mt. Kinabalu, on cliff, alt 2146–2000 m a.s.l.; 17 May 1963 M. Mizutani 2553 (Holotypus NICH252353!).

Accepted name: *Plectocolea borneensis* (Amakawa) Bakalin et S.S. Choi comb. nov. Basionym *Jungermannia borneensis* Amakawa, J. Hattori Bot. Lab. 33: 160, 1970. (=*Solenostoma borneense* (Amakawa) Váňa, Hentschel et Heinrichs, Cryptog. Bryol. 31 (2): 136, 2010).

**Figure 7:**
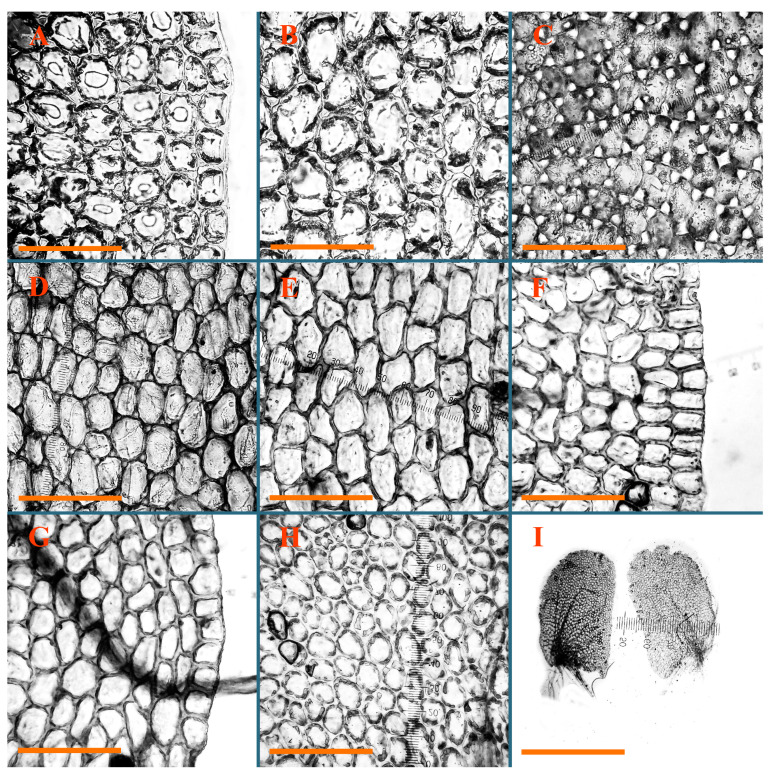
*Jungermannia borneensis* Amakawa Holotypus, NICH–252353: (**A**) leaf margin cells; (**B**) midleaf cells. *Jungermannia caelestis* Inoue et Váňa Holotypus, TNS–48164: (**C**) midleaf cells; (**D**) cells near leaf base). *Jungermannia champawatensis* S.N.Srivast. et Amakawa Isotypus, HIRO, s.n.: (**E**) midleaf cells; (**F**) leaf margin cells in leaf upper part; (**G**) leaf margin cells in leaf middle part. *Jungermannia comata* Nees Holotypus, STR, s.n.: (**H**) midleaf cells; (**I**) leaves. Scales: 100 µm for (**A**–**H**); 1 mm for (**I**).

**Figure 8:**
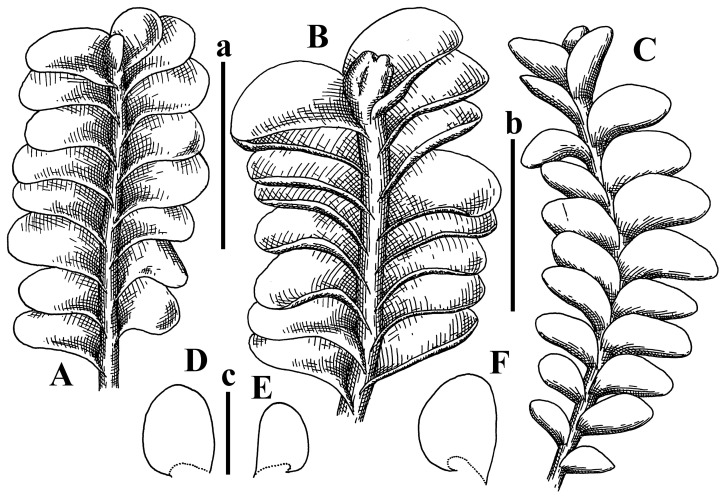
*Jungermannia borneensis* Amakawa Holotypus, NICH–252353: (**A**) plant habit dorsal view; (**B**) perianthous plant; (**C**) plant habit ventral view; (**D**–**F**) leaves. Scales: a—3 mm for (**A**,**B**); b—3 mm for (**C**); c—2 mm for (**D**–**F**).

Description: Plants yellowish brownish, prostrate to very loosely ascending, (2.5–)3–4 mm wide and 15–25 or more mm long, rather soft, but not lax. Stem 300–375 µm in diameter, not branched (even as subfloral innovations), brownish. Rhizoids rather rigid, grayish to brownish, decurrent down the stem or obliquely spreading, in the upper part of shoot and sometimes decurrent in nearly clear fascicle, originated mostly near ventral leaf bases, but not only there. Leaves 1425–2125 × 1125–1800 µm, concave–canaliculate, obliquely inserted, obliquely ovate, obliquely spreading; ‘canal’ line is slightly incurved, contiguous, rarely distant in depauperate plants, dorsally decurrent for 1/2–2/3 of stem width, ventrally clearly arcuately inserted, decurrent for 1/3 of stem width of even more. Midleaf cells 37.5–75 × 35–45 µm, thin-walled, trigones moderate in size, coarsely convex, walls brownish, cuticle smooth to obscurely striolate; cells along leaf margin 25–40 µm, thin-walled, trigones moderate in size to large, bulging, external wall noticeably thickened, cuticle smooth or loosely striolate. Dioicous. (Amakawa did not describe the female organs; he describes only male ones, but we were able to find a female and did not find a male). Only immature perianths present. Perianth nearly hidden within bracts, 5-plicate, starting from the base, more or less suddenly contracted to a non-beaked mouth, composed of subisodiametric to shortly oblong cells, shortly ellipsoidal, ca. 1000 × 800 µm; perigynium virtually absent, bracts similar to leaves, concave–canaliculate, obliquely spreading, not sheathing the perianth in the base (Figure 7A–D and Figure 8A–F).

Comment: Accepted under name *Solenostoma borneense* (Amakawa) Váňa, Hentschel et Heinrichs in [17]. In narrow generic treatment of Solenostomataceae, it should be attributed to *Plectocolea*. There is the problem of the generic placement of this plant. Although originally, it was placed in *Jungermannia* subg. *Plectocolea* (and then followed in this respect by Söderström et al. [17] with placement in *Solenostoma* subg. *Plectocolea*), the perianths of plants are composed of subisodiametric cells, and perigynium is absent. However, taking into account that the perianths in the specimens are strongly immature, and the peculiar rhizoid distribution (in a clear brownish fascicle and originating mostly near ventral leaf bases) similar to that in *Plectocolea comata,* we prefer to follow the existing classification until molecular genetic research will clarify the generic placement of the species.

*Jungermannia caelestis* Inoue et Váňa, Stud. Cryptog. Papua N. Guinea: 16, 1979.

Type: Papua New Guinea, Central District, Albert Edward, summit area, elevation about 3700–3800 m a.s.l.; October 1975, H. Inoue 24,810; (Holotypus TNS48164!; Isotypus JE04009131!)

Accepted name: *Solenostoma caeleste* (Inoue et Váňa) Váňa, Hentschel et Heinrichs, Cryptog. Bryol. 31 (2): 136, 2010.

**Figure 9:**
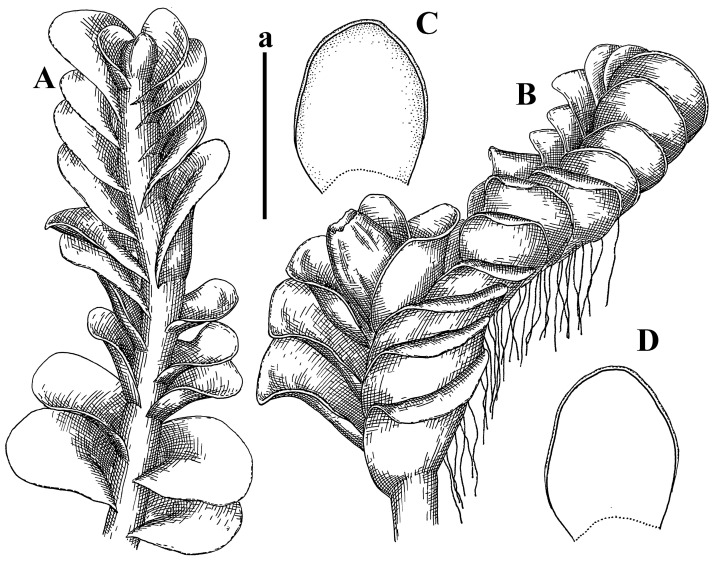
*Jungermannia caelestis* Inoue et Váňa Holotypus, TNS–48164: (**A**) plant habit; (**B**) perianthous plant; (**C**,**D**) leaves abaxial view. Scale: a—1 mm for (**A**–**D**).

Description: Plants ascending, brownish to yellowish brownish, with branches near area of their origin pinkish whitish pink, pink coloration also rarely present near shoot apices, 5–10 × (1.0–)1.25–1.75 mm. Stem 200–250 µm in diameter, brownish, sparsely laterally branched (branching lateral-intercalary, but originating very near to ventral leaf margin base). Rhizoids numerous, brownish to grayish, obliquely spreading, not forming the fascicle. Leaves contiguous, commonly enclosed with one another, obliquely ovate to obliquely elliptic, narrowly recurved along margin (more widely and obviously in the ventral leaf margin), canaliculate, obliquely spreading, sometimes slightly recurved away of the stem apex, (824−)950–1500 × (650−)725–1200 µm. Midleaf cells shortly oblong, 25–55 × 20–30 µm, walls thin, brownish, trigones large, convex, cuticle smooth; along margin 20–25 µm, thin-walled, trigones large, convex, external wall strongly thickened, cuticle smooth; basal cells 42.5–62.6 × 22.5–35 µm, thin-walled, walls brownish to yellowish brownish, trigones moderate, convex, cuticle smooth. Possibly dioicous. Perianth exerted for 1/3 of its length, shortly ellipsoidal to shortly clavate, with 4–5 plicae in upper 1/3–1/4, gradually or suddenly contracted to the non-beaked mouth, ca. 2 m log in the maturity and 1.5 mm wide, mouth sometimes pink-colored; unistratose to the base; mouth loosely crenulate, composed of subisodiametric thin-walled cells; upper part cells 25–35 × 17.5–27.5 µm, thin-walled, with large convex trigones, cuticle smooth; middle-part cells shortly oblong, 27.5–45 × 17.5–27.5 µm, cuticle smooth, trigones large, convex; lower-part cells similar to the middle. Perigynium virtually absent. Bract sheathing the perianth in lower half and obliquely spreading above, similar in size to large leaves (Figure 7C,D and Figure 9).

Comment: As the authors wrote in [18], this species is characterized by bluish coloration when fresh and similar to *S. niveum* (Grolle) R.M. Schust. ex Váňa, Hentschel & Heinrichs (differing in its unistratose midleaf) and to *S. stephanii* (Schiffn.) Steph. (differing in its smaller size (up to 2 mm wide), with oblong leaves and bluish coloration absent when plants are fresh. In the herbarium the bluish coloration is absent; the plants in the studied specimen are ca. 1.0–1.75 mm wide, and leaves are oblong, with narrowly recurved leaf margin or margin almost plane. In our opinion, the species is closely related to *S. bauerii* (Schiffn.) Steph., being, however, different in its mostly non-recurved leaf margin and moderately sized leaf cells’ trigones (the variation in these features is poorly understood).

*Jungermannia champawatensis* S.N.Srivast. et Amakawa, Proc. Natl. Acad. Sci. India, B 61 (2): 205, 1991.

Type: North-West Himalaya (India), Champawat, 1600 m a.s.l., on rock; September 1977 S.N. Srivastava 327; (Isotypus HIRO, s.n.!)

Accepted name: *Plectocolea champawatensis* (S.N.Srivast. et Amakawa) Bakalin et S.S. Choi comb. nov. Basionym: *Jungermannia champawatensis* S.N.Srivast. et Amakawa, Proc. Natl. Acad. Sci. India, B 61 (2): 205, 1991 (=*Solenostoma champawatense* (S.N.Srivast. et Amakawa) Váňa et D.G. Long, Nova Hedwigia 89 (3/4): 496, 2009).

**Figure 10:**
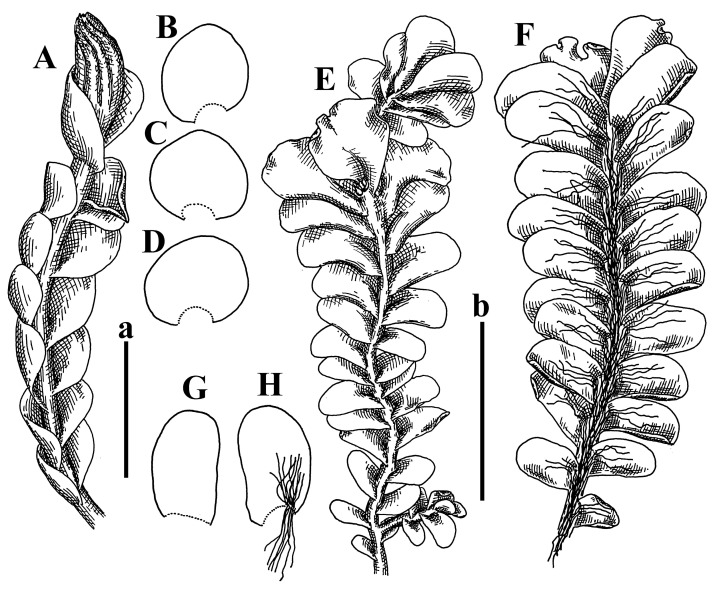
*Jungermannia champawatensis* S.N.Srivast. et Amakawa Isotypus, HIRO, s.n.: (**A**) perianthous plant; (**B**–**D**) leaves. *Jungermannia comata* Nees Holotypus, STR, s.n.: (**E**) female plant habit; (**F**) plant habit ventral view; (**G**,**H**) leaves. Scales: a—2 mm for (**A**–**D**); b—2 mm for (**E**–**H**).

Description: Plants dirty green to brownish green, more or less erect to ascending, forming loose mats, 0.7–1.5 mm wide and 15–20 mm long. Stem brownish, 150–250 µm in diameter, not branched, even as subfloral innovations. Rhizoids very dense, purple-brown, to brownish in upper part of stem, produced from the stem, more or less rigid, forming thick distinct fascicle decurrent down the stem, sometimes obliquely spreading in lower part of shoot. Leaves laterally appressed to the stem, contiguous to imbricate and then overlapping lower part of the leaf above, flattened to slightly concave, obliquely to subtransversely inserted, barely or up to 1/2 of stem width decurrent dorsally and transversely to arcuately inserted ventrally where not decurrent; in the slide, widely ovate to suborbicular well-developed leaves 1200–1400 × 1600–1700 µm. Midleaf cells subisodiametric to shortly oblong, 20–40 × 20–30 µm, thin-walled, trigones moderate in size, slightly convex, cuticle nearly smooth to indistinctly papillose-striolate; cells along leaf margin 20–34 µm, thin-walled to slightly thick-walled with thickened external wall, trigones moderate in size to rather small, concave, cuticle smooth or nearly so. Possibly dioicous. Perianth pluriplicate, strongly turbinate in upper half-1/3 of its length, exerted for 1/2–3/5 of its length, 1700–2000 × 600–700 µm, composed of elongated cells; perigynium very low to 1/5–1/4 of perianth length, with 1 pair of bracts; bracts closely sheathing perianth or loosely sheathing in upper 1/3 (Figure 7E–G and Figure 10A–D).

Comment: The species is characterized by thick brown-purple rhizoid fascicles, erect growth, laterally appressed leaves, rhizoids originating from the ventral side of the stem, a low perigynium and strongly turbinate perianth. This species somewhat resembles *Plectocolea tetragona*, although it is smaller and has no rhizoigenous cells in the leaves.

*Jungermannia comata* Nees, Enum. Pl. Crypt. Javae: 78, 1830.

Type: Indonesia, Java, “Crescit in humidis ad terram” [19], (Holotypus STR, s.n.!)

Accepted name: *Plectocolea comata* S. Hatt. Bulletin of the Tokyo Science Museum 11: 38. 1944 (=*Solenostoma comatum* (Nees) C.Gao, Fl. Hepat. Chin. Boreali-Orient.: 73, 1981)

**Figure 11:**
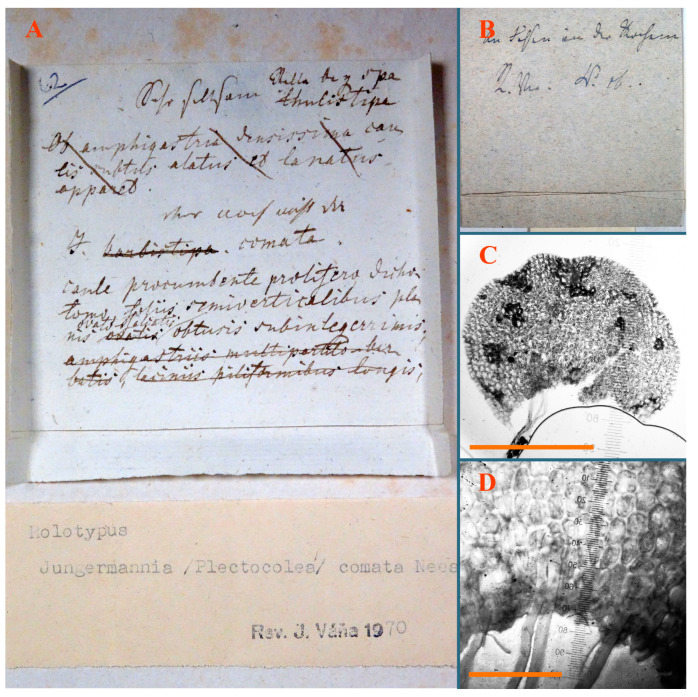
*Jungermannia comata* Nees Holotypus, STR, s.n.: (**A**) holotype label. *Jungermannia confertissima* Nees Holotypus, STR, s.n.: (**B**) holotype label; (**C**) leaf; (**D**) leaf basal cells with rhizoid initial cells. Scales: 500 µm for (**C**); 100 µm for (**D**).

Description: Plants pale greenish brownish, prostrate, plagiochiloid in appearance, in loose patches, 2250–2800 µm wide and 15–25 mm long, branched as subfloral innovations (the specimen contains only unfertilized archegonia). Rhizoids numerous, colorless, originating mostly near the ventral leaf base, but not only there, forming distinct brownish fascicle, or spreading upward along ventral side of the leaf; in the lower part of shoot, rhizoids sometimes obliquely spreading and separated, commonly purplish. Leaves obliquely inserted, dorsally shortly decurrent, obliquely to erect spreading and subhorizontally oriented, slightly convex, with dorsal margin recurved and obliquely lingulate, 1200–1250 × 750–800 µm. Midleaf cells oblong, 30–40 × 20–26 µm, thin-walled, trigones moderate to large, concave to convex, cuticle coarsely papillose; cells along leaf margin 12.5–20.0 µm, nearly thin-walled or walls slightly thickened, trigones moderate, concave, cuticle coarsely verrucose, margin crenulate. Dioicous. Archegonial bracts similar to leaves, but wider, sheathing the archegonia in the base, and convex and subhorizontally oriented above; one subfloral innovation quickly growing into normal branch (Figure 7H,I, Figure 10E–H and Figure 11A).

Comment: This is a very distinct species due to rhizoids originating mostly near ventral leaf base, forming a loose to merely distinct fascicle; some, however, go upward along the ventral leaf surface and the coarsely papillose-verrucose leaf cuticle.

*Jungermannia confertissima* Nees, Naturgesch. Eur. Leberm. 1: 291, 1833.

Type: Austria. “Salzburger Alpen, “in der Kochem” (=Wochein), F. Muller” [13], (Holotypus STR, s.n.!, Isotypus PC103265!)

Accepted name: *Solenostoma confertissimum* (Nees) Schljakov, Novosti Sist. Nizš. Rast. 17: 239, 1980.

**Figure 12:**
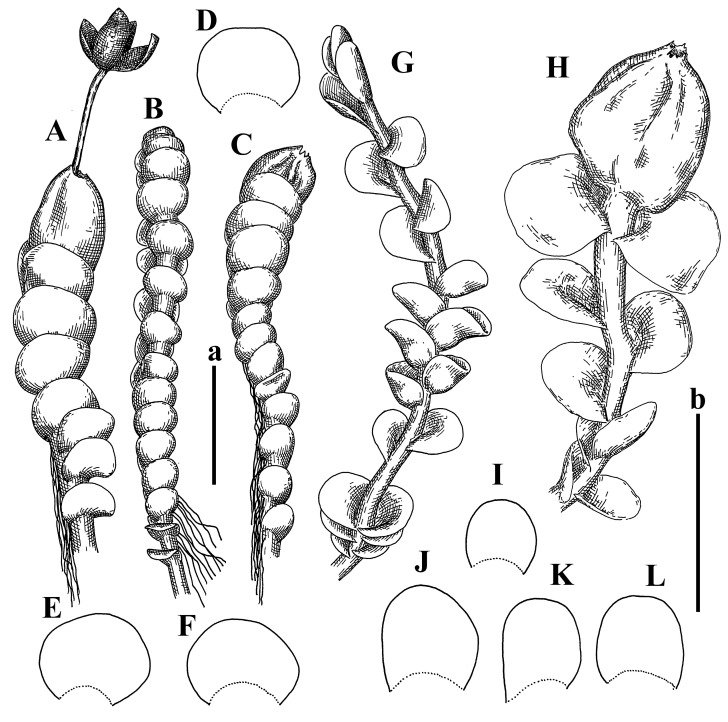
*Jungermannia confertissima* Nees Holotypus, STR, s.n.: (**A**,**C**) perianthous plants; (**B**) plant habit; (**D**–**F**) leaves. *Jungermannia crassula* Nees et Mont. Holotypus, STR, s.n.: (**G**) male plant; (**H**) perianthous plant; (**I**–**L**) leaves. Scales: a—2 mm for (**A**–**F**); b—1 mm for (**G**–**L**).

Comment: This species is distinctive due to its paroicous inflorescence, wider than long, sparsely rhizogenous leaves, and arcto-boreal (sub?)circumpolar distribution. Its questionable identity with *Solenostoma abyssinicum* is discussed above (Figure 11B–D and Figure 12A–F).

*Jungermannia crassula* Nees et Mont., Ann. Sci. Nat. Bot. (sér. 2) 5: 54, 1836.

Type: Chili, “Juan Fernandez insula, ad terram inter *Peltigeram*” [20]; Montagne (Holotypus STR, s.n.!).

Accepted name: *Solenostoma crassulum* (Nees et Mont.) Steph., Bull. Herb. Boissier (sér. 2) 1 (5): 497 (59), 1901.

**Figure 13:**
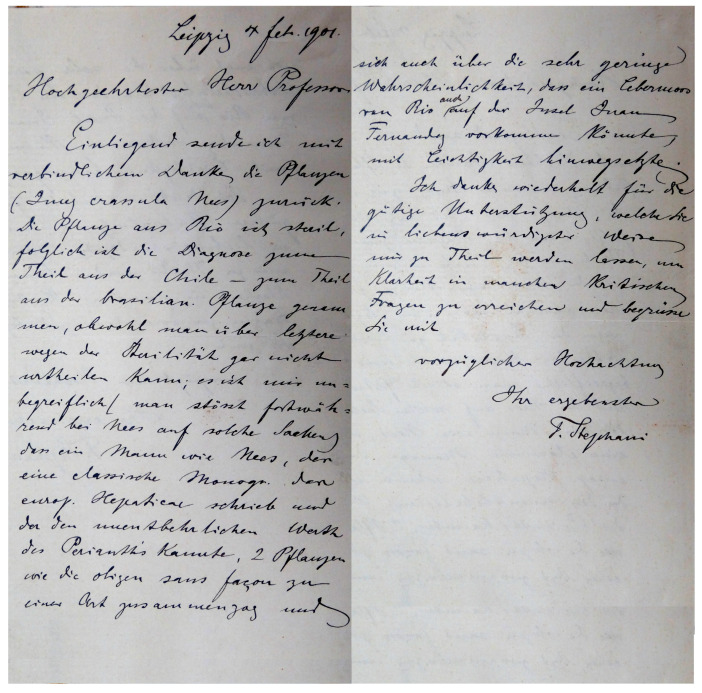
*Jungermannia crassula* Nees et Mont. Holotypus, STR, s.n.: Franz Stephani’s letter on the type status of the specimen. Dr. Alfons Schäfer-Verwimp (Germany) kindly translated this letter to English as follows: “Highly revered Professor, with many thanks I send the plants (Jungermannia crassula Nees) back to you. The plant from Rio is sterile, consequently the diagnose is partly from the Chile plant—partly from the Rio plant, though one cannot judge about the latter one because of its sterility; it is incomprehensible to me that a man like Nees who wrote a classical monograph on European Hepaticae and certainly knew the value of the perianth, two plants like the above ones united to one species without hesitation, and also overcame with ease the low probability, that a liverwort from Rio also could occur on the island of Juan Fernandez. I thank again for the generous support …”.

**Figure 14:**
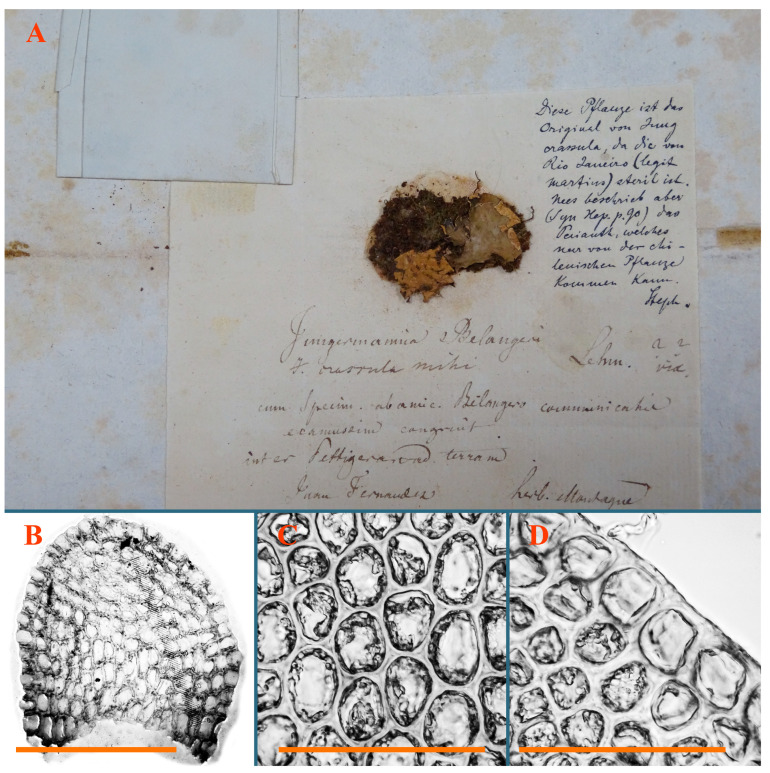
*Jungermannia crassula* Nees et Mont. Holotypus, STR, s.n.: (**A**) holotype specimen with Stephani’s note; (**B**) leaf. *Jungermannia crenuliformis* Austin Isotypus, F, s.n.: (**C**) midleaf cells; (**D**) leaf margin cells. Scales: 300 µm for (**B**); 100 µm for (**C**,**D**).

Description: Plants creeping to ascending, green to yellowish greenish, sometimes with purple-tinged margins, forming more or less dense pure mats 625–1500 µm wide (slightly wider near the perianth) and 5–7 mm long, very soft and gentle. Rhizoids not numerous, colorless, obliquely spreading. Leaves distant, suborbicular to imbricate and nearly ovate, 280–400 × 300–450 µm, plane to slightly concave, very obliquely inserted, erect spreading and subhorizontally oriented, shortly decurrent dorsally. Midleaf cells 25.0–37.5 × 15–30 µm, thin-walled, sub-isodiametric to oblong, trigones small to vestigial, concave; cells along leaf margin with moderate to large, slightly concave to convex trigones, 20–25 µm along margin. Dioicous. Androecia intercalary, with 2 pairs of bracts, possibly monandrous; bracts inflated in lower half. Perianth exerted for 3/4–4/4 of the length, ellipsoidal, 4-plicate in upper half, suddenly contracted to the beaked mouth; perigynium absent; female bracts slightly concave, obliquely to erect spreading (Figure 12G–L, Figure 13 and Figure 14A,B).

Comment: As was indicated in Synopsis Hepaticarum, the species is based on specimens of *Jungermannia belangeriana* Montagne (nom. herb., non Lehm. & Lindenb, 1835), and then re-described as *J. crassula* by Nees and Montagne. Therefore, the specimen from Juan Fernandez has the priority for the specimen that van Martius (Rio de Janeiro) first cited in the Synopsis Hepaticarum. The present specimen is the holotype of *J. crassula,* as was handwritten by Stephani (Figure 13). Another specimen in the STR *Jungermannia crassula* Paratype is from Rio de Janeiro. It is merely similar to the holotype, with the exception of more a pachydermous leaf cell structure (midleaf cells moderate in size, slightly convex trigones).

*Jungermannia crenuliformis* Austin, Bull. Torrey Bot. Club 3 (3): 10, 1872.

Type: U.S.A, on rocks and rivulets near Closter, New Jersey; Sullivant (Isotypus F, s.n.!)

Accepted name: *Plectocolea crenuliformis* (Austin) Mitt. Transactions of the Linnean Society of London, 2nd series: Botany 3(3): 198. 1891. (*Solenostoma crenuliforme* (Austin) Steph., Bull. Herb. Boissier (sér. 2) 1 (5): 494 (56), 1901)

**Figure 15:**
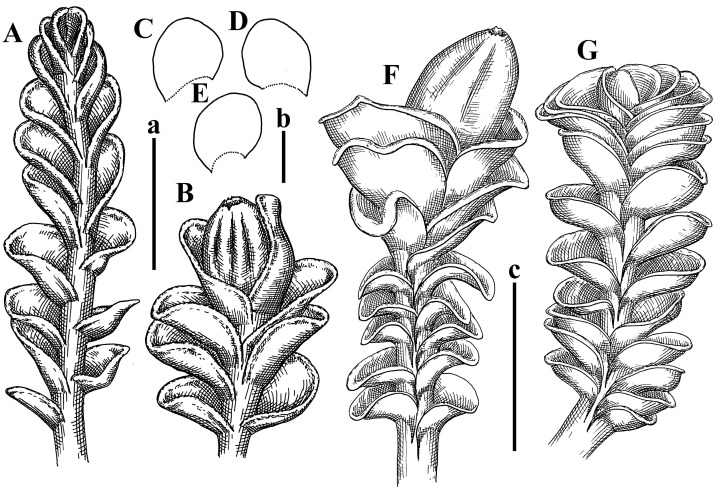
*Jungermannia crenuliformis* Austin Isotypus, F, s.n.: (**A**) plant habit; (**B**) perianthous plant; (**C**–**E**) leaves. *Jungermannia diversiclavellata* Amakawa et Grolle Holotypus, NICH–286094: (**F**) perianthous plant; (**G**) plant habit. Scales: a—1 mm for (**A**,**B**); b—500 µm for (**C**–**E**); c—3 mm for (**F**,**G**).

Plants prostrate to loosely ascending, yellowish brownish, sometimes with purplish to rusty pigmentation near ventral leaf base, perigynium and adjacent areas and (rarely) in the leaf lamina, but not in leaf margin, 0.8–1.4 mm wide and 3–5 mm long, forming dense mat. Stem brownish, not branched, with the exception of ventral subfloral innovations (1 per gynoecium), growing into a normal branch, 160–260 µm in diameter. Rhizoids common, brownish to brown and purple brown, decurrent along ventral side of stem, sometimes forming indistinct fascicle or erect spreading, and forming loose mat under the stem. Leaves mostly contiguous, rarer distant or subimbricate and then enclosed with another, obliquely inserted, dorsally short or up to 1/2 of stem width decurrent, ventrally arcuately inserted and up to 1/2 of stem width decurrent, concave–canaliculate to concave, widely obliquely ovate to obliquely reniform and transversely elliptic, 720–800 × 880–976 µm. Midleaf cells subisodiametric, thick-walled, trigones moderate in size, convex to triangle, 24.0–36.8 × 22.4–32.0 µm; cells along leaf margin thick-walled, larger than intramarginal, trigones moderate in size to large, mostly concave, rarely convex, sometime confluent, cuticle smooth. Dioicous. Androecia intercalary with 2(3) pairs of bracts, bracts smaller than leaves, moderately inflated in lower half and canaliculate and obliquely spreading above. Perianth conical, pluriplicate, hidden within bracts or exerted for 1/4–1/3 of the length, composed of elongate, pellucid cells; perigynium ca. 1/2–2/2 of perianth length, with 1 pair of bracts; female bracts sheathing perianth and canaliculate, not spreading above (Figure 14C,D and Figure 15A–E).

*Jungermannia diversiclavellata* Amakawa et Grolle, J. Hattori Bot. Lab. 31: 107, 1968.

Type: Papua New Guinea “NE New Guinea, Mt. Wilhelm, by L. Aunde, on soil., 3800 m” [7]; 1965 Hewson 173 (Holotypus NICH286094!, original label provide the name ‘*heteroclavellata’*, however in original description it is ‘*diversiclavellata’*)

Accepted name: *Solenostoma diversiclavellatum* (Amakawa et Grolle) R.M.Schust. ex Váňa et D.G. Long, Nova Hedwigia 89 (3/4): 501, 2009.

Description: Plants ascending to somewhat prostrate, greenish brownish to yellowish brownish, purplish near perianth base, in loose patches, 3.0–3.5 mm wide, 8–15 mm long. Stem not branched, with the exception of rare subfloral innovations, yellowish. Rhizoids numerous, brownish to deep brownish, forming distinct fascicle decurrent down the stem or, sometimes, obliquely spreading, then in the fascicles or separated, originated both from ventral leaf lamina and ventral side of stem (not from the perianth). Leaves obliquely inserted, dorsally decurrent for 1/2–2/2 of stem width, ventrally arcuately inserted, commonly shortly, or up to 1/2 of stem width decurrent, contiguous to subimbricate and then enclosed with one another, concave–canaliculate, along margin narrowly revolute, widely obliquely ovate to widely obliquely triangular, 1600–1920 × 2080–2720 µm. Midleaf cells sub-isodiametric to shortly oblong, 38.4–64.0 × 40.0–48.0 µm, trigones moderate in size, convex, walls thin, cuticle smooth. Cells along leaf margin 40.0–56.0 µm, thin-walled, external wall thick, trigones moderate, convex, cuticle smooth. Dioicous. Androecia intercalary, with 8–10 pairs of bracts (or more), different generation divided by 2(3) pairs of leaves, 1–2-androus, similar to leaves, but inflated in lower 1/3. Perianth 5-plicate, gradually to more or less suddenly contracted to non- beaked mouth, ca. 4000 × 1800 µm, loosely or densely rhizogenous in lower half of the perianth; if purplish-colored there, the produced rhizoids also have a purplish tint, unistratose for almost whole extent (bistratose in lower 1/5), composed of subisodiametric thin-walled cells; perigynium very low (no higher than 1/6 of perianth length; female bracts concave–canaliculate, loosely sheathing perianth, similar to leaves, but larger (Figure 15F,G).

*Jungermannia flagellaris* Amakawa, J. Hattori Bot. Lab. 29: 258, 1966.

Type: Nepal, Vorhimalaja, Okhaldunga, Abies-Rhododendron-Wald um Thodung Serting, am Block, 3200 m a.s.l.; 1962 Poelt H30 (Holotypus NICH276488!)

Accepted name: *Solenostoma flagellare* (Amakawa) Váňa et D.G.Long, Nova Hedwigia 89 (3/4): 501, 2009.

**Figure 16:**
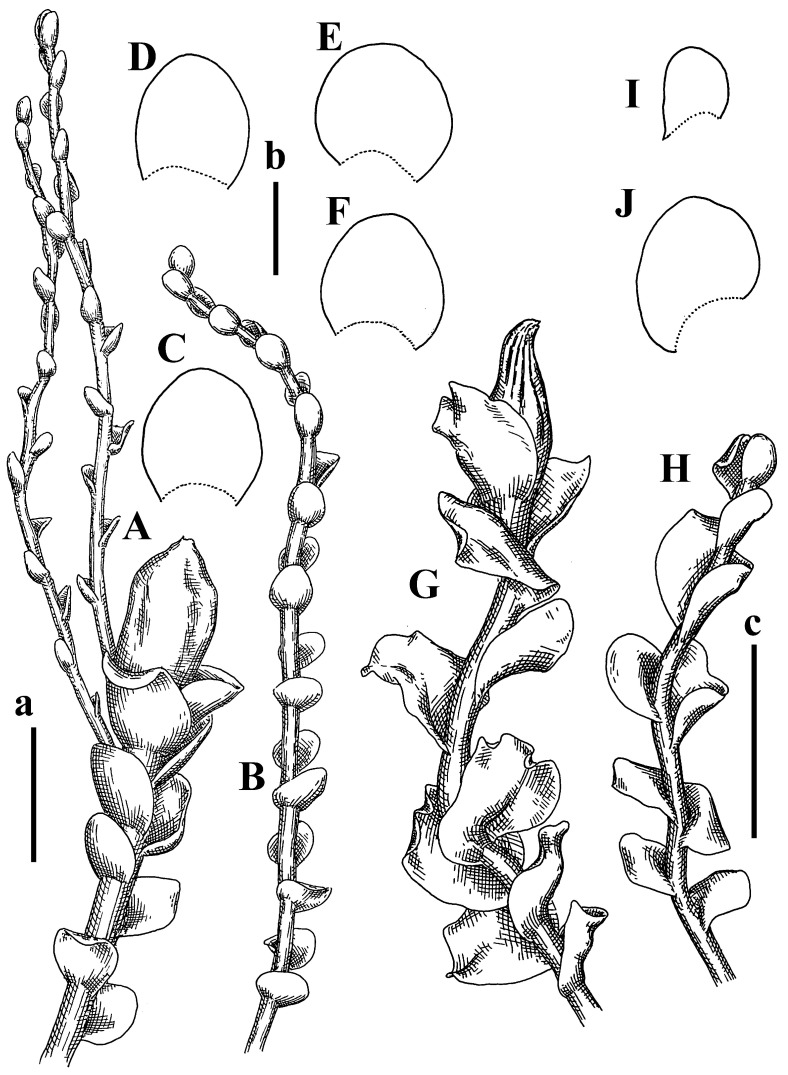
*Jungermannia flagellaris* Amakawa Holotypus, NICH–276488: (**A**) perianthous plant; (**B**) plant habit; (**C**–**F**) leaves. *Jungermannia flavialbicans* Amakawa et Grolle Holotypus, NICH–286093: (**G**) perianthous plant; (**H**) plant habit; (**I**,**J**) leaves. Scales: a—1 mm for (**A**,**B**); b—300 µm for (**C**–**F**); c—2 mm for (**G**–**J**).

**Figure 17:**
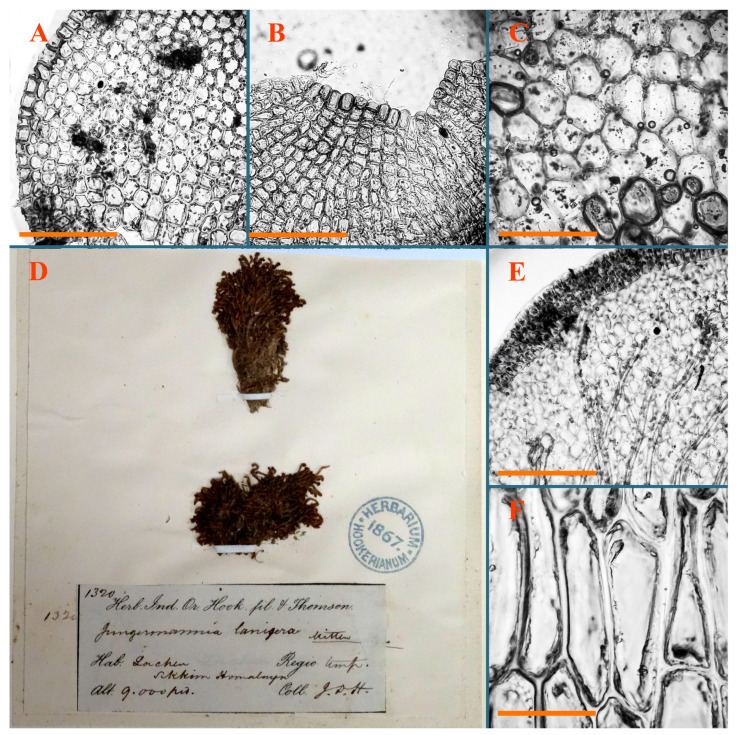
*Jungermannia flagellaris* Amakawa Holotypus, NICH–276488: (**A**) leaf cells; (**B**) perianth mouth). *Jungermannia flavialbicans* Amakawa et Grolle Holotypus, NICH–286093: (**C**) midleaf cells. *Jungermannia flavorevoluta* Váňa Isotypus, BM, s.n.: (**D**) isotype label; (**E**) leaf margin cells. *Jungermannia fossombronioides* Austin Isotypus, F, s.n.: (**F**) cells in perianth middle part. Scales: 100 µm for (**A**–**C**,**E**); 50 µm for (**F**).

Description: Plants filiform, brownish to brown, sometimes with purplish to rusty tint near apices (but not in the perianth), 0.25–0.5 mm wide (near perianth to 1 mm wide) and 3–10 mm long, nearly prostrate. Stem 60–130 µm in diameter, brownish to brown, rarely laterally branched, with very common lateral and ventral subfloral innovations (1–3 per perianth); branches and subfloral innovations grow into normally developed branches, but not fertilized (at least we did not observe this). Rhizoids sparse to nearly absent, brownish, erect spreading, separated or united into unclear fascicles, rarely forming a mat under the stem. Leaves distant, nearly concave to canaliculate-concave, in 3–4 pairs below the perianth slightly inflated in lower half as if they contain antheridia (however, they were not found), suborbicular to mostly ovate, 260–380 × 250–410 µm (1:0.9–1.1), obliquely to subtransversely inserted, not or barely decurrent in both sides. Midleaf cells obscurely thickened, shortly elongated, 15.0–22.5 × 12.5––17.5 µm, trigones small, concave, cuticle smooth; cells along margin 12.5–17.5 µm, with obscurely thickened walls, trigones small, concave, external wall obscurely thickened, cuticle smooth. Possibly dioicous. Perianth with 1–3 subfloral innovations, obovate to nearly clavate, very loosely 3-plicate in upper 1/3, gradually narrowing to the beaked mouth, exerted for 2/3 of its length, bistratose in lower third, ca. 900 × 450 µm, perianth mouth crenulate, composed of shortly elongated cells with obscurely thickened walls; perianth middle-part cells composed of subisodiametric cells, 12.5–20 × 7.5–12.5 µm, thin-walled, trigones vestigial to very small; perigynium virtually absent, bracts similar to large leaves (Figure 16A–F and Figure 17A,B).

Comment: This species is the type of *Solenostoma* sect. *Nematocaulon*.

*Jungermannia flavialbicans* Amakawa et Grolle, J. Hattori Bot. Lab. 31: 108, 1968.

Type: New Guinea, “NE New Guinea. Morobe District, road Wau to Edie Creek, 2000 m, sandstone, but running water” [7]; 1965, Hewson 454 (Holotypus NICH286093!)

Accepted name: *Plectocolea flavialbicans* (Amakawa et Grolle) Bakalin et S.S. Choi comb. nov. Basionym: *Jungermannia flavialbicans* Amakawa et Grolle, J. Hattori Bot. Lab. 31: 108, 1968 (=*Solenostoma flavialbicans* (Amakawa et Grolle) Váňa et D.G.Long, Nova Hedwigia 89 (3/4): 501, 2009).

Description: Plants glaucous in color (pale brownish to whitish), prostrate to ascending, 1.5–2.4 mm wide and 10–25 mm long, lax and soft. Stem 125–250 µm in diameter, whitish, not branched, even as subfloral innovations. Rhizoids colorless to grayish, rather dense, obliquely to erect spreading, separated or united into unclear fascicles, and mostly forming loose mat under stem. Leaves obliquely to very obliquely inserted, obliquely to subhorizontally oriented, obliquely spreading, dorsally decurrent for 1/2–3/2 of stem width, ventrally subtransversely inserted, not or barely decurrent, distant to contiguous; in general, outline mostly flattened to variously curved, commonly with undulate leaf margins, large leaves widely obliquely ovate to transversely elliptic and reniform, loosely crispate (very rarely emarginate at apex) at margin to (smaller leaves) ovate to obliquely ovate, 1100–1800 × 1100–2200 µm. Midleaf cells very thin-walled, subisodiametric to shortly oblong, penta- to hexagonal, very thin-walled, 32.5–52.4 × 25.0–45.0 µm, trigones small to very small, concave, cuticle loosely papillose; cells along leaf margin 25–40 µm, thin-walled, with small, concave trigones, external wall also thin, cuticle slightly striolate-papillose. Dioicous. Androecia intercalary, with 4–6 pairs of bracts; bracts strongly inflate in lower third and obliquely spreading and strongly undulate above. Perianth conical, pluriplicate, gradually narrowing to the mouth, ca. 2.0 × 1.0 mm, composed of elongated cells; perigynium ca 1/3 of perianth length, with 1 pair of bracts; female bracts nearly reniform, strongly undulate at margin, similar to large leaves (Figure 16G–J and Figure 17C).

Comment: the species is very closely morphologically similar to *Plectocolea grossitexta* Steph. S. Hatt. (although in the latter rhizoids, color may be purplish and brownish) in lax texture and large leaf cells. The robust difference is the distribution (New Guinea versus Japan). The pale color is one of the bright features of this species.

*Jungermannia flavorevoluta* Váňa, J. Hattori Bot. Lab. 36: 63, 1972 [1973].

Type: India, Sikkim Himalaya, 9000 ft.; J.D. Hooker 1320 (Isotypus BM, s.n.!).

Accepted name: *Solenostoma flavorevolutum* (Váňa) Váňa et D.G. Long, Nova Hedwigia 89 (3/4): 501, 2009.

**Figure 18:**
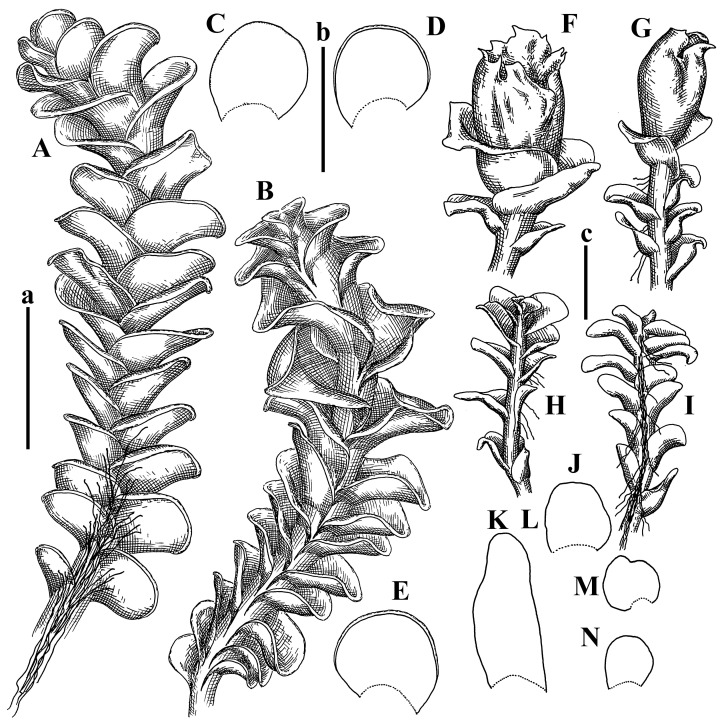
*Jungermannia flavorevoluta* Váňa Isotypus, BM, s.n.: (**A**) plant habit ventral view; (**B**) plant habit dorsal view; (**C**) leaf adaxial view; (**D**,**E**) leaf abaxial view. *Jungermannia fossombronioides* Austin Isotypus, F, s.n.: (**F**,**G**) perianthous plants; (**H**,**I**) plant habit; (**K**) female bract; (**J**,**L**–**N**) leaves. Scales: a—1 mm for (**A**,**B**); b—1 mm for (**C**–**E**); c—1 mm for (**F**–**N**).

Description: Plants erect, more or less rigid, yellowish brownish, with markedly purple-colored androecial bracts, sparsely laterally branched, 1250–1500 µm wide and 8–15 mm long. Rhizoids numerous, brownish (especially when united into fascicle), originating in outer surface of leaf lamina, decurrent down in ventral or dorsal side of the stem in thick (thicker that stem) distinct fascicle. Leaves subimbricate, obliquely inserted, but subtransversely oriented (which in imbricate leaved shoots gives impression of transversely inserted leaves), decurrent for 1–3 stem widths ventrally and for 1–1.5 of stem widths dorsally, in most cases narrowly or more widely revolute or recurved along margin, obliquely transversely elliptic; when flattened in the slide, 750–900 × 950–1375 µm. Midleaf cells 20.0–32.5 × 15.0–20.0 µm, thin-walled, trigones moderate to large in size, slightly convex, cuticle smooth; cells along margin 7.5–17.5 µm, nearly thin-walled or walls unequally thickened due to trigones’ confluence, external wall slightly thickened, trigones mostly large, but sometimes moderate, cuticle smooth. Dioicous. Androecia (only one generation is observed at shoot apices; therefore, it is not known whether it is intercalary androecia or terminal, and stem will then die) with 3–4 or more(?) pairs of bracts; bracts purple-colored, 4–5-androus, stalk biseriate (this feature was seen unclearly) (Figure 17D,E and Figure 18A–E).

Comment: Due to its strongly rhizogenous leaves and distinct rhizoid fascicle, this species indeed resembles *Solenostoma lanigerum*, as the specimen was originally named by Mitten. However, its smaller cells and narrowly revolute leaf margin distinguish *S. flavorevolutum* from *S. lanigerum*. Váňa [21] discusses the differences from *Solenostoma clavellatum,* which are in brownish to brownish greenish coloration, smooth cuticle, and copious rhizoids originating from leaves and perianths.

*Jungermannia fossombronioides* Austin, Proc. Acad. Nat. Sci. Philadelphia 21: 220, 1869.

Type: U.S.A., On rocks and rivulets near Closter, New Jersey (Isotypus F, s.n.!).

Accepted name *Plectocolea fossombronioides* (Austin) Mitt. Transactions of the Linnean Society of London, 2nd series: Botany 3(3): 198. 1891. (=*Solenostoma fossombronioides* (Austin) R.M.Schust., Hepat. Anthocerotae N. Amer. 2: 1027, 1969.

**Figure 19:**
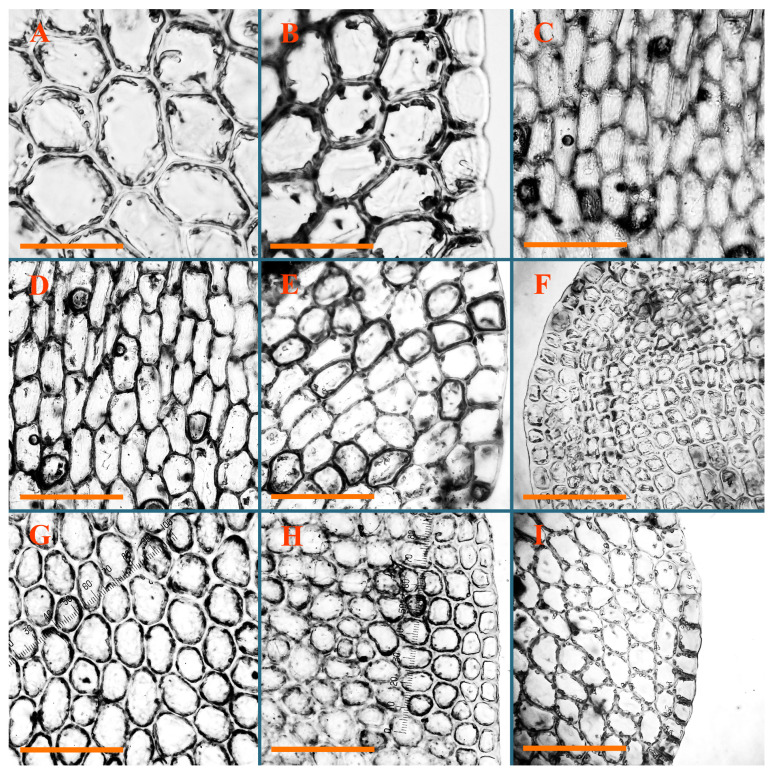
*Jungermannia fossombronioides* Austin Isotypus, F, s.n.: (**A**) midleaf cells; (**B**) leaf margin cells. *Jungermannia glauca* Amakawa Isotypus TNS–174427: (**C**) leaf base cells showing papillae; (**D**) leaf base cells; Holotypus, NICH–236837: (**E**) leaf margin cells. *Jungermannia grollei* Amakawa Holotypus, NICH–276490: (**F**) leaf margin cells. *Jungermannia heterolimbata* Amakawa Isotypus, HIRO, s.n.: (**G**) midleaf cells; (**H**) leaf margin cells. *Jungermannia hewsoniae* Amakawa et Grolle Holotypus, NICH–286092: (**I**) leaf margin cells. Scales: 50 µm for (**A**,**B**); 100 µm for (**C**–**I**).

Description: Plants prostrate to loosely ascending, yellowish brownish, forming loose to (rarely) dense patches, 0.8–1.5 mm wide and 5–10 mm long, more or less soft textured, pellucid. Stem 176–320 µm in diameter, not branched, with rare exception for ventral subfloral innovations. Rhizoids rather common, rarely sparse, purple to purple brown and (rarer) purplish, decurrent down along the stem, where sometimes forming distinct fascicle or obliquely spreading and forming loose mat under stem, closely attaching plants to the substratum. Leaves distant to contiguous, mostly concave–canaliculate, obliquely to almost erect spreading or even deflexed away of the apex, large leaves undulate along margin, obliquely inserted, dorsally barely or up to 1/2–2/3 of stem width decurrent, ventrally transversely to arcuately inserted, not or shortly (less than 1/4 of stem width) decurrent, lingulate to widely ovate, rarely crispate along margin, 960–1100 × 880–1150 µm. Midleaf cells thin- to slightly thick-walled, trigones small, concave, cells shortly oblong, 32.0–60.0 × 35.0–48.0 µm, cuticle smooth; cells along leaf margin 20.0–32.0 µm, thin-walled, trigones small, concave, cuticle smooth. Paroicous. Androecia with 3–4 pairs of bracts, just below female bracts, male bracts strongly to loosely inflated in lower half. Perianth cylindrical to obovate, gradually narrowing to the non-beaked mouth, pluriplicate, with 4–5 main plicae, not turbinate at mouth, composed of elongated cells, bistratose in lower half, cells in the perianth middle ca. 70.0–120.0 × 12.8–32.0 µm, thin- to slightly thick-walled, trigones small, concave, cuticle smooth; perigynium low, ca. 1/5 of perianth length, with 1 pair of bracts; female bracts closely sheathing perianth in lower 1/2 to whole length or erect spreading to recurved with undulate and crispate leaf margin above (Figure 17F, Figure 18F–N and Figure 19A,B).

*Jungermannia glauca* Amakawa, Fl. E. Himalaya: 511, 1966.

Type: East Nepal, Baroya Khimty—Thakma Khola, 2500–3000 m alt.; 16 November 1963, H. Kanai, G. Murata & M. Togashi (Holotypus NICH236837!; Isotypus TNS174427!)

Accepted name: *Plectocolea glauca* (Amakawa) Bakalin Arctoa 23: 102. 2014. (=*Solenostoma glaucum* (Amakawa) Váňa et D.G.Long, Nova Hedwigia 89 (3/4): 502, 2009).

**Figure 20:**
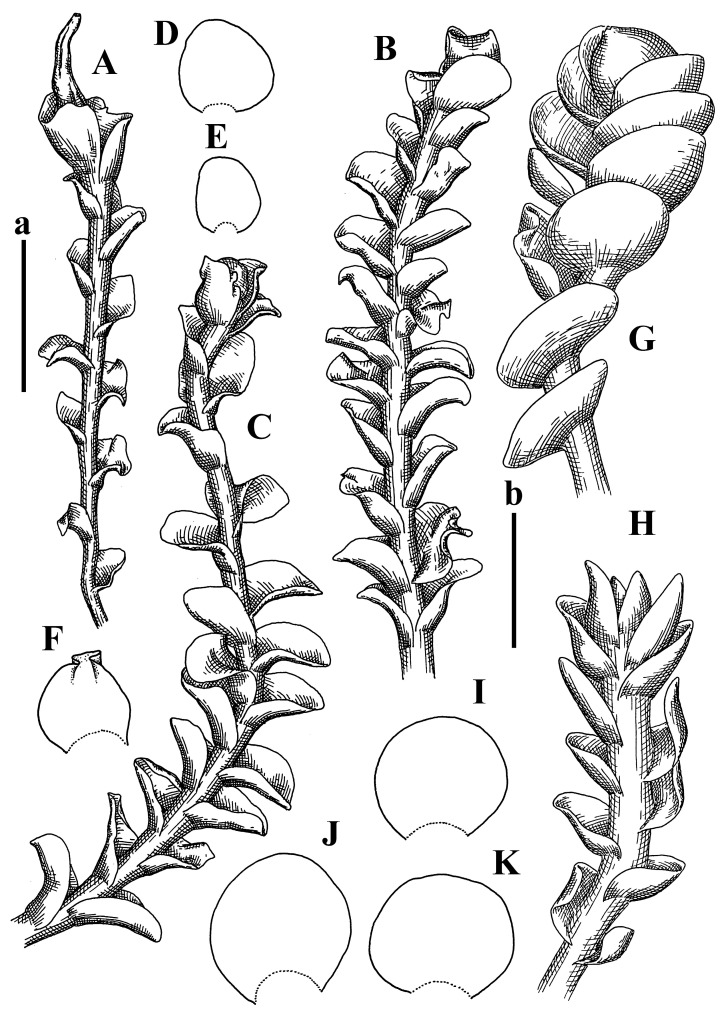
*Jungermannia glauca* Amakawa Holotypus, NICH–236837: (**A**) perianthous plant; (**B**) plant habit; TNS–174427 (**C**) plant habit; (**D**–**F**) leaves. *Jungermannia grollei* Amakawa Holotypus, NICH–276490: (**G**) perianthous plant; (**H**) plant habit; (**I**–**K**) leaves. Scales: a—3 mm for (**A**–**F**); b—500 µm for (**G**–**K**).

Description: Plants whitish with blackish tint, especially obvious near leaf margins in the shoot apex. Rhizoids in thin, but more or less distinct fascicle decurrent down the stem, colorless to grayish. Midleaf cells thin-walled, oblong, 45–75 × 32–42 µm, trigones small, concave, cuticle smooth to papillose-striolate; cells along leaf margin 30–42.5 µm, loosely thickened, trigones small, concave, cuticle smooth to loosely striolate-papillose. Dioocous. Androecia intercalary, with 8–12 pairs of bracts, inflated in lower half and canaliculate and obliquely spreading above (‘canal’ line commonly slightly decurved down). Perianth long-conical, 2200–3000 × 600–1000 µm, exerted for 1/2 of its length, perigynium virtually absent, female bracts similar to leaves (Figure 19C–E and Figure 20A–F).

Comment: Amakawa [22] pointed out some rhizoids originate from initial leaf cells; we did not find this feature in the holotype, although were able to observe it in the isotype (TNS), probably because rhizogenous leaves are quite rare in this species. The plant color and characteristics are similar to travertine.

*Jungermannia grollei* Amakawa, J. Hattori Bot. Lab. 29: 260, 1966.

Type: Nepal, Vorhimalaya, Okhaldunda, Abies-Rhododendron-Wald, um Thodung, 3000 m a.s.l.; 1962, Poelt H16 (Holotypus NICH276490!).

Accepted name: *Solenostoma riclefii* Váňa et D.G.Long, Nova Hedwigia 89 (3/4): 507, 2009. (*nom. nov. pro Jungermannia grollei* Amakawa)

Description: Plants prostrate to loosely ascending in perianthous tips, brownish with obvious purple tint or brown–purple-colored near apex, 0.5–0.7 mm wide and 3–5 mm long, closely attached to the substratum. Stem 100–120 µm in diameter, not branched, with rare exceptions for ventral subfloral innovations. Rhizoids more or less dense, colorless, erect spreading in unclear fascicles, closely attaching plant to the substratum. Leaves concave to canaliculate–concave, subtransversely inserted, contiguous to imbricate, not or barely decurrent in both sides, 320–400 × 420–520 µm, suborbicular to widely ovate, obliquely spreading and nearly transversely oriented or covering lower half of the next leaf. Midleaf cells 22.5–35 × 12.5–20.0 µm, shortly oblong, thin-walled, trigones moderate in size, nearly concave to slightly convex, cuticle loosely papillose to papillose–striolate and smooth; cells along leaf margin 17.5–25 µm, trigones large, mostly concave, nearly confluent (cells therefore have strongly unequally thickened walls, external wall noticeably thickened, cuticle smooth or nearly so). Possibly dioicous. Perianth nearly hidden within bracts or exerted for 1/4 of its length, purple-colored, 3-plicate in upper 1/3, suddenly contracted to the distinct beak, ca. 400 × 300 µm; perigynium virtually absent; bracts similar to leaves or slightly wider (Figure 19F and Figure 20G–K).

Comment: The species is morphologically similar to *Solenostoma pusillum* (C.E.O. Jensen) Steph., but differs in possibly dioicous inflorescence and unequally thickened cell walls along leaf margin (where there are sometimes confluent trigones).

*Jungermannia heterolimbata* Amakawa, J. Hattori Bot. Lab. 30: 183, 1967.

Type: Indian West Bengal, near Sandakphu, Darjeeling area, 11,900–11,600 ft. alt. [3627–3535 m alt.], on wet cliff; 26 April 1965 Z. Iwatsuki and A.J. Sharp B689 (Isotypus HIRO, s.n.!; the holotype should be in NICH, but not located there).

Accepted name: *Solenostoma heterolimbatum* (Amakawa) Váňa et D.G.Long, Nova Hedwigia 89 (3/4): 503, 2009.

**Figure 21:**
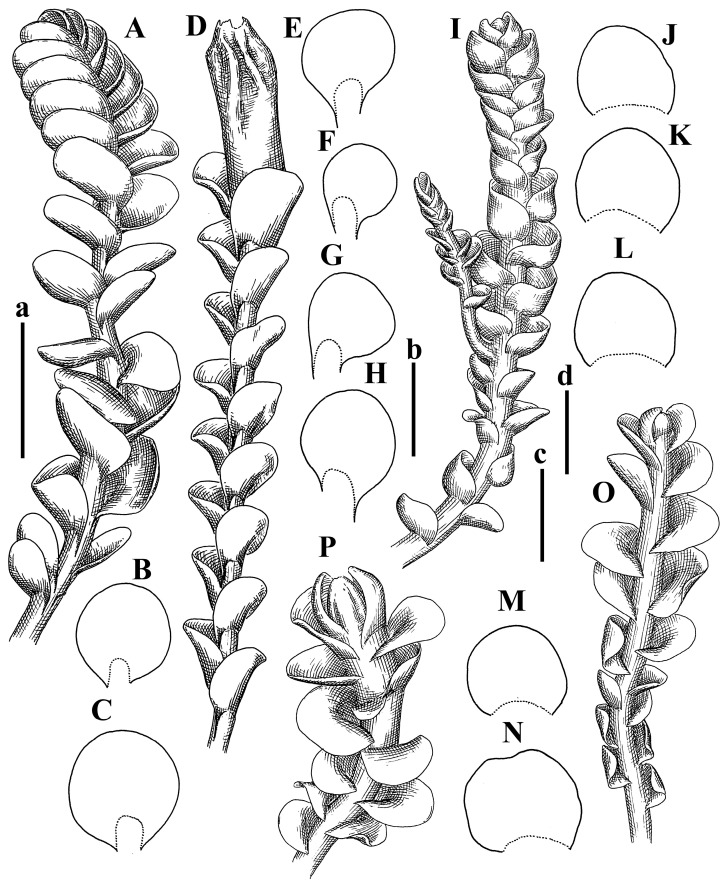
*Jungermannia heterolimbata* Amakawa Isotypus, HIRO, s.n.: (**A**) plant habit; (**B**,**C**) leaves. *Jungermannia hewsoniae* Amakawa et Grolle Holotypus, NICH–286092: (**D**) perianthous plant; (**E**–**H**) leaves. *Jungermannia hokkaidensis* Váňa Syntypus, NICH–53520: (**I**) male branch; Holotypus, NICH–53484: (**J**–**N**) leaves; (**O**) plant habit; (**P**) perianthous plant. Scales: a—2 mm for (**A**–**C**); b—1 mm for (**D**–**H**); c—1 mm for (**I**,**O**,**P**); d—500 µm for (**J**–**N**).

Description: Plants large, robust, 2.5–3.0 mm wide and 15–20 mm long, yellowish brownish erect. Stem ca 350 µm in diameter, sparsely laterally branches (commonly lateral branches stop development shortly). Rhizoids colorless to brownish, decurrent down the stem and forming more or less distinct fascicle or rhizoids sparse and decurrent down, but not forming fascicle. Leaves distant to continuous, subtransversely inserted and oriented, decurrent dorsally for 1/2–1/1 of stem width, ventrally decurrent for 1–2(3) stem widths, obliquely spreading, concave-canaliculate to concave, 1400–1600 × 1700–1800 µm, nearly orbicular. Midleaf cells subisodiametric to shortly oblong, thin-walled to obscurely thick-walled, 26–40 × 20–34 µm, trigones moderate in size, triangle to slightly concave or convex, sometimes with visible median lamina, cuticle smooth; cells along leaf margin 10–20 µm, unequally thick-walled, with large to moderate in size, triangle to concave trigones, sometimes confluent in tangential walls, cuticle smooth (Figure 19G,H and Figure 21A–C).

*Jungermannia hewsoniae* Amakawa et Grolle, J. Hattori Bot. Lab. 31: 108, 1968.

Type: Papua New Guinea, “Morobe Distr., road Wau to Edie Creek, 2000 m, on sandstone” [7]; 1965, Hewson 456; (Holotypus NICH-286092!).

Accepted name: *Solenostoma hewsoniae* (Amakawa et Grolle) R.M.Schust. ex Váňa, Hentschel et Heinrichs, Cryptog. Bryol. 31 (2): 136, 2010.

**Figure 22:**
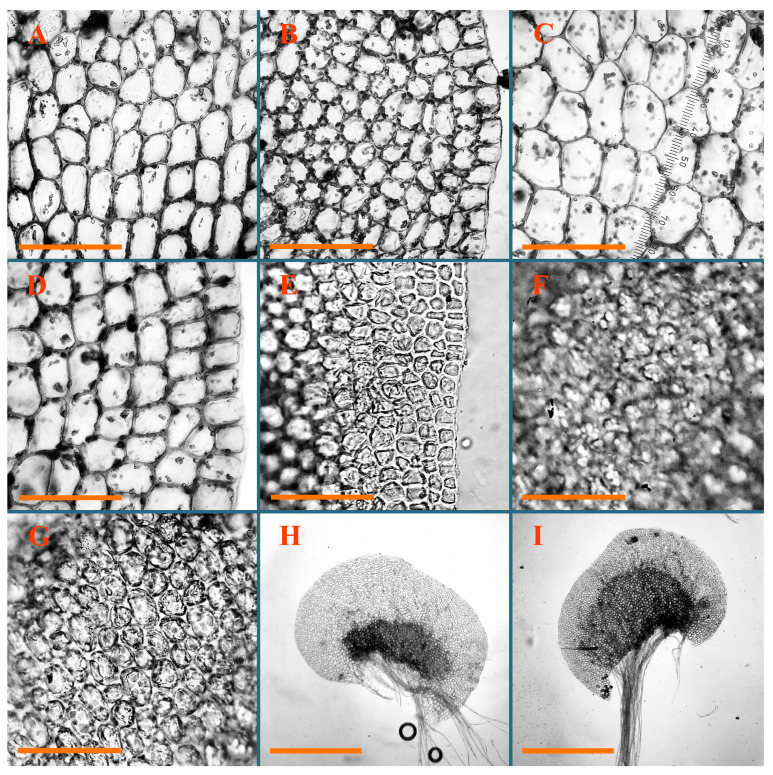
*Jungermannia hewsoniae* Amakawa et Grolle Holotypus, NICH–286092: (**A**) midleaf cells. *Jungermannia hokkaidensis* Holotypus, NICH–53484: (**B**) leaf margin cells. *Jungermannia hyalina* Lyell Lectotypus, BM–000968932: (**C**) midleaf cells; (**D**) leaf margin cells. *Jungermannia lanigera* Mitt. Isolectotypus, PC–010354: (**E**) leaf margin cells; (**F**) midleaf cells showing papillae; (**G**) midleaf cells; (**H**,**I**) leaves. Scales: 100 µm for (**A**–**G**); 500 µm for (**H**,**I**).

Description: Plants erect in loose patches, 0.8–1.5 mm wide and 15–25 mm long, yellowish brownish, more of less rigid. Stem 150–300 µm in diameter, brownish to brown, not branched, even as subfloral innovations. Rhizoids colorless to grayish, sparse to numerous, in well-developed shoots forming loose to more or less distinct thin fascicle decurrent down the stem, or, in weaker shoots, separated and obliquely spreading. Leaves concave–canaliculate, loosely sheathing the stem in the base, and obliquely spreading above (thus ‘canal’ line decurved down), distant, subtransversely to transversely inserted, decurrent in both sides for 1–2 stem widths, 750–1000 × 950–1200 µm, transversely elliptic, sometimes loosely crispate along margin, rarely truncate to almost emarginate at apex. Midleaf cells subisodiametric to oblong, thin-walled, walls brownish, 25.0–42.5 × 20.0–30 µm, trigones moderate in size, convex, cuticle smooth; cells along leaf margin 15–30 µm, more or less thin-walled to obscurely thickened, trigones moderate in size, convex, cuticle smooth. Dioicous. Androecia with 8–10 and more pairs of bracts, intercalary, bracts inflated in lower 1/2–2/3 and recurved along margin above. Perianth 2500 × 800 µm, exerted for 4/5 of its length, gradually narrowing to non-beaked mouth, 4--plicate in upper 1/4 (where it becomes narrowed), unistratose to base, composed of more or less elongated cells, in the perianth middle ca. 45–100 × 20–32 µm, thin-walled, with moderate in size to small concave to slightly convex trigones, cuticle nearly smooth, but striolate in lower 1/4 of the perianth; perigynium virtually absent; female bracts similar to large leaves, sheathing perianth or concave–canaliculate and obliquely spreading (Figure 19I, Figure 21D–H and Figure 22A).

*Jungermannia hokkaidensis* Váňa, J. Hattori Bot. Lab. 35: 314, 1972. (nom. nov. pro *Jungermannia subelliptica* var. *nana* Amakawa Journal of the Hattori Botanical Laboratory 22: 21. f. 17: m–t. 1960.; =*Plectocolea nana* Amakawa nom. herb.)

Type: Japan. Hokkaido, Rishiri Island, near summit of Rishiri, 1600–1719 m a.s.l., on sandy soil in gravelly ‘alpine garden’; 22 July 1954, D. Shimizu (Holotypus NICH53484!) Syntypus is another specimen with the same label but having herbarium barcode number NICH53520!).

Accepted name: *Solenostoma hokkaidense* (Váňa) Váňa, Hentschel et Heinrichs, Cryptog. Bryol. 31 (2): 137, 2010.

Description: Plants prostrate to ascending in perianthous shoots, yellowish brownish (color probably changed due to age), more or less rigid, 0.8–1.7 mm wide, 5–8 mm long, forming loose mats, sometimes encrusted by soil particles. Stem 200–300 µm in diameter, slightly wider near perigynium, not branched, with rare exception of subfloral innovations, whitish when dry, with thin-walled cells in outer layer, 13–20 µm in diameter, becoming more thick-walled inward, where similar in size, and in central part becoming larger and again thin-walled, up to 30 µm in diameter. Rhizoids colorless to brownish, sparse to numerous, erect spreading in unclear fascicles and forming mat under the stem. Leaves distant to contiguous, obliquely inserted, concave to slightly convex, not or barely decurrent dorsally, subtransversely to arcuately inserted ventrally, where not or barely decurrent, nearly reniform, sometimes retuse at apex, 480–530 × 560–700 µm, obliquely spreading and obliquely oriented. Midleaf cells 20–32 × 17–25 µm, thin-walled, moderate in size, triangle to slightly concave or convex trigones, cuticle smooth; cells along leaf margin 17–25 µm, thin-walled, with moderate-in-size, slightly convex trigones, external wall slightly thickened, cuticle smooth. Dioicous. Perianth nearly hidden within bracts to shortly (up 1/4 of the length) exerted, conical, ca. 300 × 600 µm, sometimes with 1 subfloral innovation, composed of elongated (sometimes slightly) cells; perigynium ca. 2–2.5 perianth length, with 2 pairs of bracts, upper pair smaller than sterile leaves, closely sheathing the perianth, lover pair similar to leaves, mostly emarginate at apex (Figure 21I–P and Figure 22B).

Comment: (1) The androecia description from NICH-53520 is as follows: androecia intercalary, spicate, different generations divided by 1(2) pairs of sterile leaves, with 4–6 pairs of bracts, bracts cupped, with more or less narrowly recurved leaf margin, purplish in inflated area, 1–2-androus.

(2) The species resembles a small form of *Solenostoma obscurum* (A. Evans) R.M. Schust. from which it differs in colorless to brownish rhizoids (versus purple) and outer cells in the stem cross-section, although they are thin-walled as in *S. obscurum*; however, they are not different in size from the intramarginal layer, which has only slightly larger cells.

(3) The species is morphologically similar to West Asian *Solenostoma subtilissimum*. Vana (1972b: 314) distinguished *S. hokkaidense* from *S. subtilissimum* “by more oval to ovoid perianth and very small androecia (only 1–3 pairs of bracts)”. The latter feature is not applicable due to large variation, as also shown by the present description.

*Jungermannia hyalina* Lyell, Brit. Jungermann.: Table 63, 1814.

Type: Great Britain, “New forest”; 1813, Lyell (Lectotypus designated by Váňa [23] BM-000968932!).

Accepted name: *Solenostoma hyalinum* (Lyell) Mitt., Nat. hist. Azores: 319, 1870.

**Figure 23:**
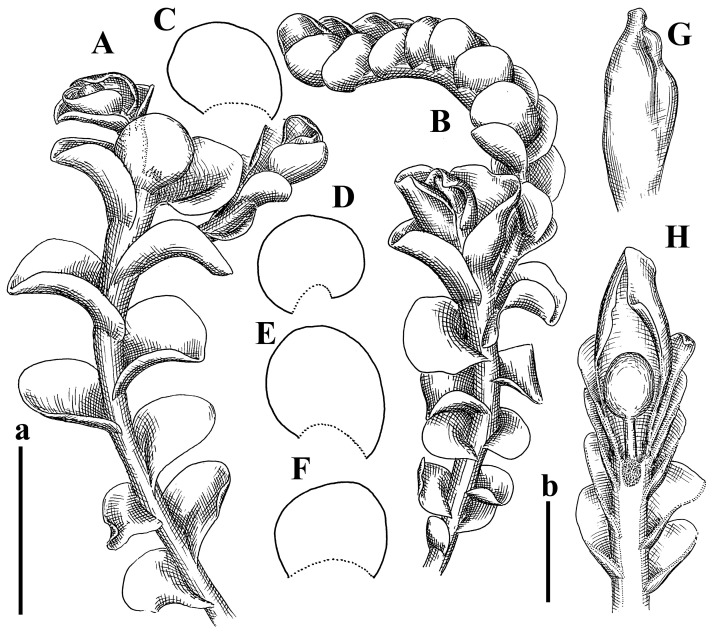
*Jungermannia hyalina* Lyell Lectotypus, BM–000968932: (**A**,**B**) female plants; (**C**–**F**) leaves. *Jungermannia lanigera* Mitt. Isolectotypus, PC–010354: (**G**) perianth; (**H**) longitudinal section of the apex of perianthous plant in lateral plane. Scales: a—2 mm for (**A**–**F**); b—1 mm for (**G**,**H**).

Description: Plants prostrate to ascending, brownish in herbarium, hardly can be called ‘hyaline’, in loose mat, 1250–2800 µm wide, frequently laterally branched in several pairs of leaves below the perianth. Rhizoids common to numerous, brownish, sometimes with slight violet tint, obliquely to erect spreading, separated or united into unclear fascicles or forming mat under stem, originating from ventral side of stem (more intensively near ventral leaf bases). Leaves mostly contiguous, to slightly distant, or m smaller shoots subimbricate, very obliquely inserted, dorsally decurrent for 1/2–3/2 of stem width, ventrally subtransversely inserted, not decurrent, in normally developed shoots’ leaves from loosely sheathing base obliquely spreading and obliquely to subhorizontally oriented, slightly concave–canaliculate to almost plane, loosely undulate at margin, leaves in smaller shoots subimbricate, concave, not decurrent dorsally; when flattened in the slide obliquely reniform to slightly obliquely transversely elliptic, sometimes with truncate apex, 800–1500 × 1125–2250 µm, large leaves sometimes slightly crispate along margin. Midleaf cells 32,5–65.0 × 25.0–40.0 µm, nearly thin-walled, trigones moderate, nearly concave to triangular, cuticle smooth; cells along margin 22.5–32.5 µm, nearly thin-walled, external wall thickened, trigones moderate in size to small, concave. Dioicous (androecia not seen). Perianth terminal with 1–2 subfloral innovations, composed of oblong cells, nearly conical to couple–shaped, 3–5-plicate, gradually narrowing to not beaked mouth, exerted for 1/4 of the length or hidden within bracts; perigynium 1/2–2/3 of perianth length, with one pair of bracts; bracts sheathing the perianth for the almost whole extent and then obliquely spreading in upper 1/3, undulate at the margin (Figure 22C,D and Figure 23A–F).

Comment: The identity of the specimens from East Asia commonly referred to *Solenostoma hyalinum* may be questioned.

*Jungermannia lanigera* Mitt., J. Proc. Linn. Soc., Bot. 5 (18): 91, 1860 [1861].

Type: India, Sikkim, Chola 10,000–14,000 ft.; 06 October 1848 J.D. Hooker no. 1318, (Lectotypus designated by Amakawa [5] NY (not seen); Isolectotypes BM010890919!; NICH225128!; PC0103541!).

Accepted name: *Solenostoma lanigerum* (Mitt.) Váňa et D.G.Long, Nova Hedwigia 89 (3/4): 503, 2009.

**Figure 24:**
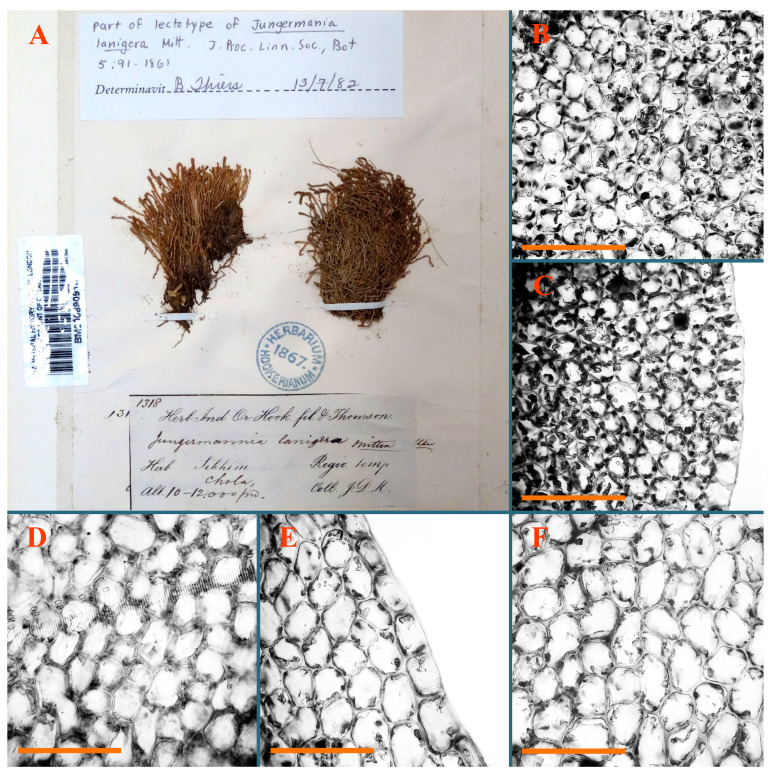
*Jungermannia lanigera* Isolectotypus BM010890919: (**A**) isolectotype specimen label. *Jungermannia monticola* f. *major* Holotypus, NICH–12732: (**B**) midleaf cells; (**C**) leaf margin cells. *Jungermannia obovata* Nees Lectotypus, STR, s.n.: (**D**) midleaf cells. *Jungermannia ohbae* Amakawa, Holotypus, NICH–318218: (**E**) leaf margin cells; (**F**) midleaf cells. Scales: 100 µm for (**B**–**F**).

**Figure 25:**
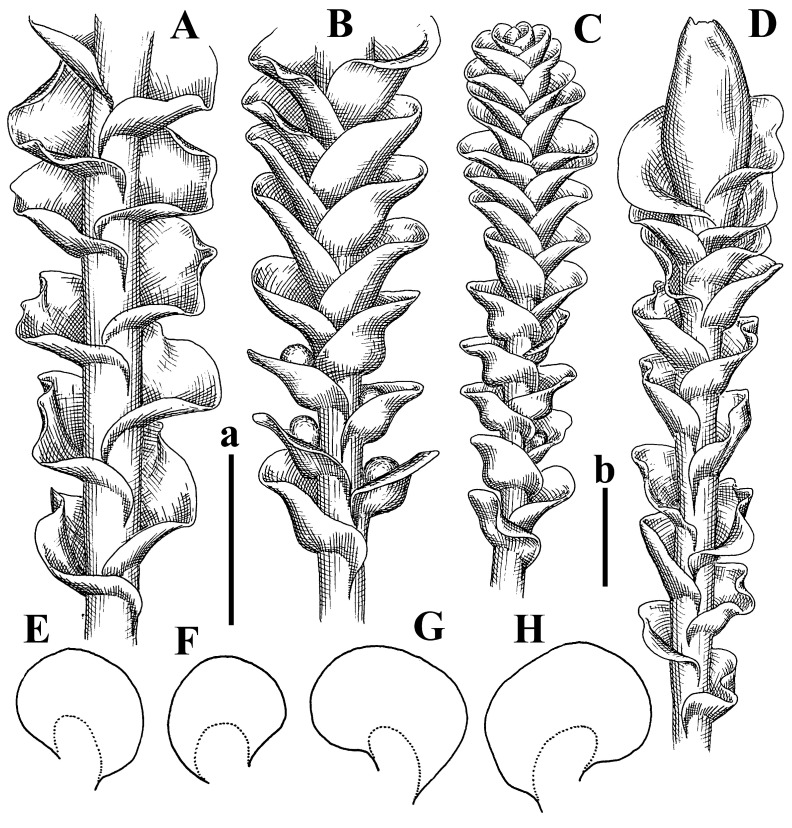
*Jungermannia lanigera* Mitt. Isolectotypus, PC–010354: (**A**) plant habit fragment; (**B**) male plant fragment; (**C**) male plant; (**D**) perianthous plant; (**E**–**H**) leaves. Scales: a—1 mm for (**A**,**B**); b—1 mm for (**C**–**H**).

Description: Plants rigid, erect, in general view (in the patch) yellowish brownish to whitish yellowish, when separated become more darkly colored due to brownish to rusty brownish coloration of stem and lower halves of leaves (the latter sometimes acquire red brown pigmentation) and hyaline to whitish very pale brownish upper halves of leaves, 1500–1750 µm wide and 15–25 mm long. Normal branches are not seen, but immature and very short branches in the sinuses of leaves in several pairs below perianth as well as in the sinuses of male bracts (after antheridia are destroyed due to age) are seen (probably some of them will give the start for ordinary branches, that, however, is not obvious). Rhizoids numerous, originated from leaf lamina and perianth (with the exception of upper 1/4 of the letter), nearly colorless to brownish, decurrent down in the distinct fascicle commonly along dorsal side of the shoot. Leaves contiguous to subimbricate, obliquely inserted, ventrally decurrent for 1–1.5 of stem widths, dorsally decurrent for 1–2 stem widths, loosely sheathing the stem in lower third and obliquely spreading, loosely concave–canaliculate above, subtransversely to obliquely oriented, commonly undulate along margin (at least larger ones) when wet, transversely elliptic to obliquely reniform when flattened in the slide, 625–750 × 1125–1625 µm. Midleaf cells 17.5–35.0 × 15.0–22.5 µm, thin-walled, trigones moderate in size, slightly convex, cuticle distinctly papillose; cells along leaf margin 7.5–15.0 µm, unequally thickened due to trigones’ confluence, trigones large, nearly triangle to slightly concave or convex. Dioicous. Androecia intercalary, with 5–7 pairs of bracts, nearly spicate, with strongly inflated lover half and recurved upper third, 2–4-androus, stalk biseriate, ca 30–40 µm long. Perianth terminal, nearly fusiform, ca. 2300 × 1000 µm, exerted for 1/2–2/5 of the length, loosely plicate in upper third, gradually narrowing to the non-beaked mouth, 2–3-stratose in lower 3/4 of the length; perigynium vestigial or at least less than 1/5 of perianth length; bracts similar to leaves, undulate, sheathing the perianth (Figure 22E–I, Figure 23G,H, Figure 24A and Figure 25).

Comment: Abundantly rhizogenous leaves, rhizoids in distinct fascicle (sometimes going along dorsal side of the stem) and plane leaf margin distinguish the species.

*Jungermannia monticola* f. *major* S.Hatt., J. Hattori Bot. Lab. 3: 8, 1948 (1950).

Type: Japan, Yakushima Island, between Miyanoura and Hananoego, ca 1800 m a.s.l., granitic rock; 27 September 1940, S. Hatttori 7804 (Holotypus NICH12732!).

Accepted name: *Solenostoma major* (S. Hatt.) Bakalin & Vilnet The Bryologist 115(4): 574. 2012.

**Figure 26:**
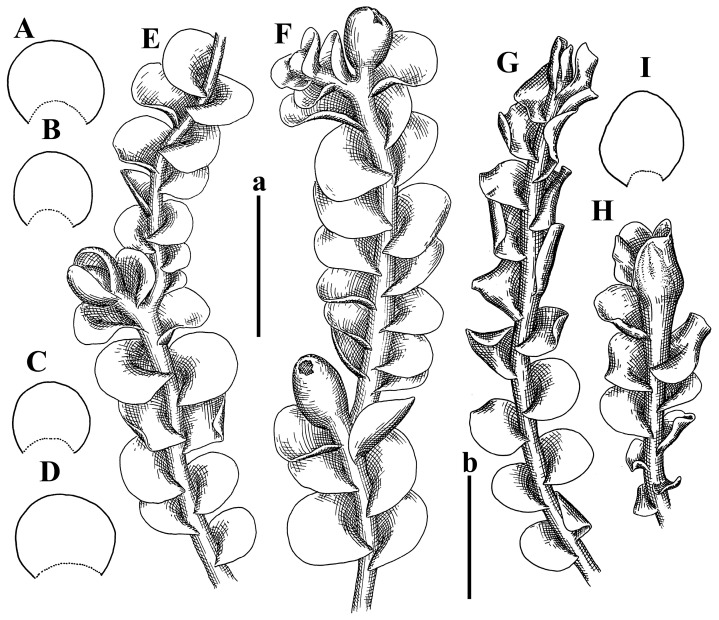
*Jungermannia monticola* f. *major* S.Hatt. Holotypus, NICH–12732: (**A**–**D**) leaves; (**E**) plant habit; (**F**) perianthous plant. *Jungermannia obovata* Nees Lectotypus, STR, s.n.: (**G**) plant habit; (**H**) perianthous plant; (**I**) leaf. Scales: a—2 mm for (**A**–**F**); b—2 mm for (**G**–**I**).

Description: Plants brownish green to yellowish greenish (probably due to age), prostrate to ascending, 1.5–2.5 mm wide, 10–20 mm long, forming loose mats. Stem flexuous, 200–300 µm in diameter, not branched, with the exception of subfloral innovations, shortly growing into normal branches and fertilizing again. Rhizoids numerous, colorless to grayish, obliquely spreading, uniting in loose fascicles or forming loose mat under stem. Leaves obliquely to subtransversely inserted, concave to canaliculate–concave and nearly plane, contiguous, widely ovate to transversely elliptic, 850–1150 × 1100–1500 µm, obliquely spreading and nearly obliquely oriented, dorsally shortly (not more than 1/4 of stem width) decurrent, ventrally sub- to transversely inserted, not or barely decurrent. Midleaf cells subisodiametric, 17–30 × 17–30 µm, more or less thin-walled, with moderate-in-size, slightly convex to concave trigones, cuticle smooth; cells along leaf margin 20–25 µm, thin-walled, with thickened external wall, trigones moderate in size to large, convex, cuticle smooth. Possibly dioicous. Perianth (only unfertilized perianths were found) with one subfloral innovation (innovation is so strong that perianth sometimes looks situated on lateral short branch, but not on main axis), obovate, smooth to loosely 3--plicate in upper 1/4, suddenly contracted to narrowly beaked mouth on truncate or slightly depressed apex, bistratose in lower half, composed of subisodiametric thin-walled cells, with moderate-in-size, convex trigones, mouth crenulate; perigynium absent; bracts similar in size to leaves, concave to loosely sheathing the perianth in lower half and with recurved margin in upper 1/3–1/4 (Figure 24B,C and Figure 26A–F).

Comment: This taxon was discussed by Bakalin and Vilnet [24]. It is characterized by a strongly inflated (in upper part loosely plicate) perianth and colorless rhizoids forming a mat along the ventral side of the stem. The most morphologically similar is *Solenostoma sunii* Bakalin et Vilnet, as discussed in [11].

*Jungermannia obovata* Nees, Naturgesch. Eur. Leberm. 1: 332, 1833 (Nees 1833c).

Type: Czech Republic, “Krkonoze, Upska jama” [4], 28 June 1824; Flotow (Lectotypus designated by Váňa [4] STR, s.n.!)

Accepted name: *Solenostoma obovatum* (Nees) C.Massal., Epat. erb. critt. ital.: 17, 1903.

**Figure 27:**
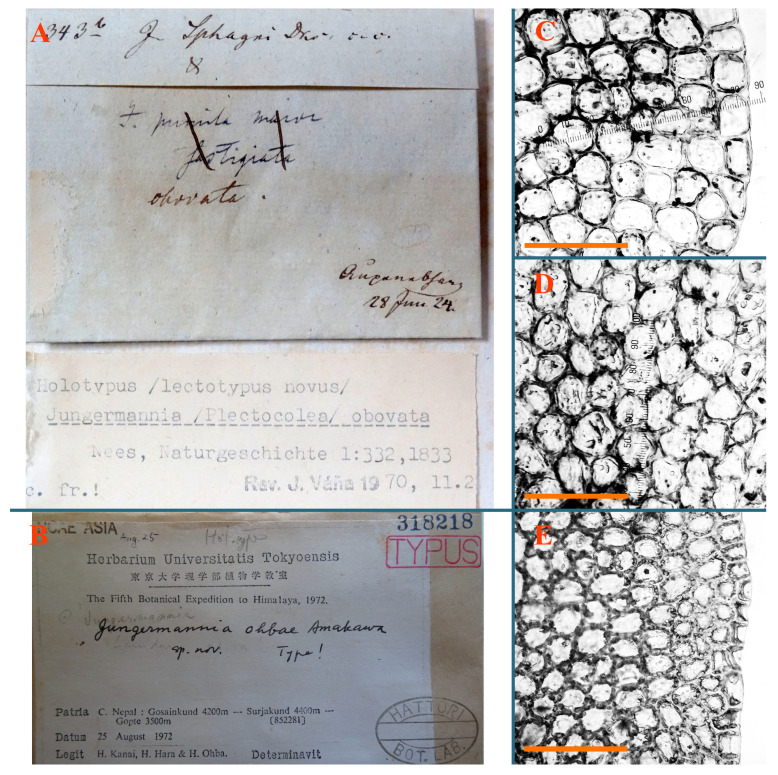
*Jungermannia obovata* Nees Lectotypus, STR, s.n.: (**A**) lectotype label. *Jungermannia ohbae* Amakawa, Holotypus, NICH–318218: (**B**) holotype label. *Jungermannia pfleidereri* Amakawa & Váňa Isotypus, HIRO, s.n.: (**C**) leaf margin cells; (**D**) midleaf cells. *Jungermannia plagiochilacea* Grolle Holotypus, NICH–73009: (**E**) leaf margin cells. Scales: 100 µm for (**C**,**D**,**E**).

Description: Plants presumable erect, merely rigid, yellowish brownish to greenish brownish, 1000–2500 µm wide. Rhizoids purple to purplish brown, numerous, obliquely spreading in unclear fascicles. Leaves distant to contiguous; larger leaves are undulate along margin (probably due to material age), ovate to obliquely ovate, 1000–1375 × 1050–1500 µm, concave–canaliculate to nearly plane, obliquely inserted, shortly to 2/3 of stem width decurrent dorsally, obliquely to erect spreading and obliquely oriented. Midleaf cells 25.0–50.0 × 25.0–35.0 µm, thin-walled, trigones moderate to small, concave, cuticle densely papillose–striolate; cells along margin 20–25 µm, thin-walled, trigones small. Paroicous. Perianth conical, hidden within bracts or very shortly exerted, perigynium ca 2 perianth lengths, female bracts appressed to the perianth, sometimes with loosely recurved margin (Figure 24D, Figure 26G–I and Figure 27A).

Comment: This species is indeed very closely related to *Solenostoma subellipticum* (Lindb. ex Heeg) R.M. Schust., although the latter differs in colorless to pinkish rhizoids and also more northern distribution. Moreover, we did not see the typical *S. obovatum* in North-East Asia, all so named specimens may belong to *S. subellipticum* in the narrow sense.

*Jungermannia ohbae* Amakawa, Bull. Univ. Mus. Univ. Tokyo 8: 218, 1975.

Type: Central Nepal, Gosainkund 4200 m a.s.l.—Surjakund 4400 m a.s.l.—Gopte 3500 m a.s.l.; 25 August 1972, H. Kanai, H. Hara, H. Ohba (Holotypus NICH318218!) Note: the herbarium number in Hattori [25] is indicated NICH312380, but the specimen with that number was not located in NICH, while the number cited above is marked as the type.

Accepted name: *Solenostoma ohbae* (Amakawa) C.Gao, Bryofl. Xizang: 495, 1985.

**Figure 28:**
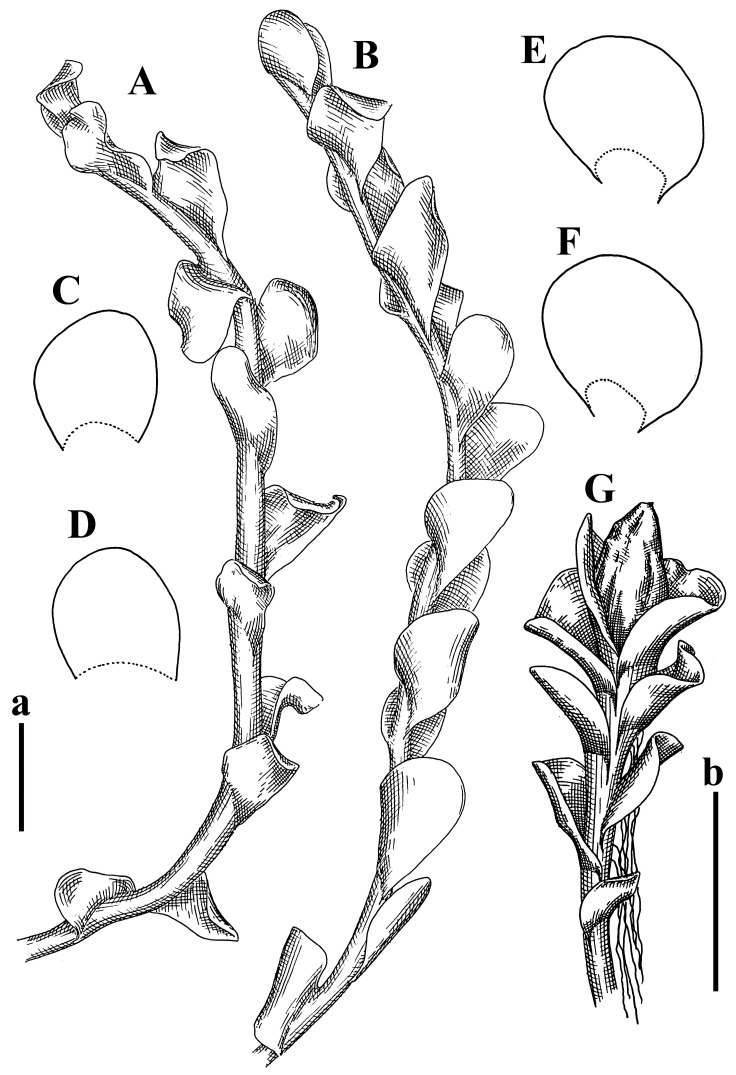
*Jungermannia ohbae* Amakawa, Holotypus, NICH–318218: (**A**,**B**) plant habit; (**C**,**D**) leaves. *Jungermannia pfleidereri* Amakawa & Váňa Isotypus, HIRO, s.n.: (**E**,**F**) leaves; (**G**) perianthous plant fragment. Scales: a—1 mm for (**A**–**D**); b—1 mm for (**E**–**G**).

Description: Plants prostrate to ascending, lax, soft, brownish to purplish, purple color more deep in shoot apices and (sometimes) ventral leaf bases. Rhizoids sparse, colorless to grayish, erect spreading, separated or united into unclear fascicles. Leaves distant, very obliquely inserted, decurrent for 1–2 stem width in dorsal side, ventrally subtransversely to arcuately inserted, not or up 2/3 of stem width decurrent, nearly ovate to obliquely ovate, 1100–1200 × 1200 µm, sometimes with more deeply colored rim (although leaf rim cells are not thickened). Midleaf cells nearly subisodiametric, thin-walled, 37–50 × 30–38 µm, walls brownish to pinkish, trigones small to moderate in size, concave; cells along leaf margin 25–38 µm, thin-walled, trigones moderate in size, concave, external wall thickened, cuticle smooth. Possibly dioicous. Perianth obovate to nearly ob-conical, with 1–3 subfloral ventral innovations, suddenly contracted to beaked mouth, not deeply 3–4-plicate in truncate apex, composed of subisodiametric cells (Figure 24E,F, Figure 27B and Figure 28A–D).

Comment: Purple-colored shoot apices, very obliquely inserted leaves and suddenly truncate obconical perianth are distinctive in this species.

*Jungermannia pfleidereri* Amakawa & Váňa Journal of the Hattori Botanical Laboratory 35: 388. 1972.

Type: India orientalis, Madura; Pfleiderer (Isotypus HIRO, s.n.!)

Accepted name: *Plectocolea pfleidereri* (Amakawa & Váňa) Bakalin et S.S. Choi comb. Nov. Basionym: *Jungermannia pfleidereri* Amakawa & Váňa Journal of the Hattori Botanical Laboratory 35: 388. 1972. (=*Solenostoma pfleidereri* (Amakawa & Váňa) Sushil K. Singh Liverw. Hornw. India, 267, 2016).

Description: Plants prostrate, yellowish brownish, prostrate to loosely ascending, more or less closely attached to the substratum, 0.7–1.5 mm wide and 5–8 mm long, soft. Stem not branched, brownish, 100–150 µm in diameter. Rhizoids numerous, obliquely spreading to decurrent down the stem and forming indistinct fascicle, brownish to purplish. Leaves distant to contiguous, obliquely inserted, decurrent for 0.5–1.5 of stem widths in dorsal side, subtransversely to arcuately inserted dorsally, where not or barely decurrent, mostly truncate to emarginate at apex, slightly undulate, slightly concave–canaliculate, narrowly obliquely spreading to sheathing the stem (and then sometimes covering lower part of the next leaf closer to apex), transversely elliptic to reniform, 700–800 × 1000–1400 µm. Midleaf cells nearly isodiametric, 20–40 × 20–35 µm, thin-walled, trigones moderate in size, triangle to slightly concave or convex, cuticle smooth; cells along leaf margin 20–36 µm, thin-walled, trigones moderate in size, concave to slightly convex, external wall slightly thickened, cuticle smooth or nearly so. Perianth brownish, conical, ca. 1100 × 650 µm, pluriplicate, exerted for 1/2 of its length; perigynium ca. 1/2 of perianth length, with 1 pair of bracts, bracts loosely sheathing perianth in lower half and loosely obliquely spreading above (Figure 27C,D and Figure 28E–G).

*Jungermannia plagiochilacea* Grolle, J. Hattori Bot. Lab. 58: 197, 1985 (Based on *Jungermannia plagiochiloides* Amakawa, n. illeg. (later homonym) Journal of the Hattori Botanical Laboratory 22: 25. f. 20. 1960. auct. non *Jungermannia plagiochiloides* (Spruce) Mitt. Timehri 5: 221. 1886.

Type: Japan, Fukuoka Pref., Tsukushi-yabakei, on wet rock; 15 September 1957 T. Amakawa 2461 (Holotypus NICH-73009!).

Accepted name: *Plectocolea plagiochilacea* (Grolle) Bakalin et S.S. Choi comb. nov. Basionym: *Jungermannia plagiochilacea* Grolle, J. Hattori Bot. Lab. 58: 197, 1985. (=*Solenostoma plagiochilaceum* (Grolle) Váňa et D.G.Long, Nova Hedwigia 89 (3/4): 505, 2009).

**Figure 29:**
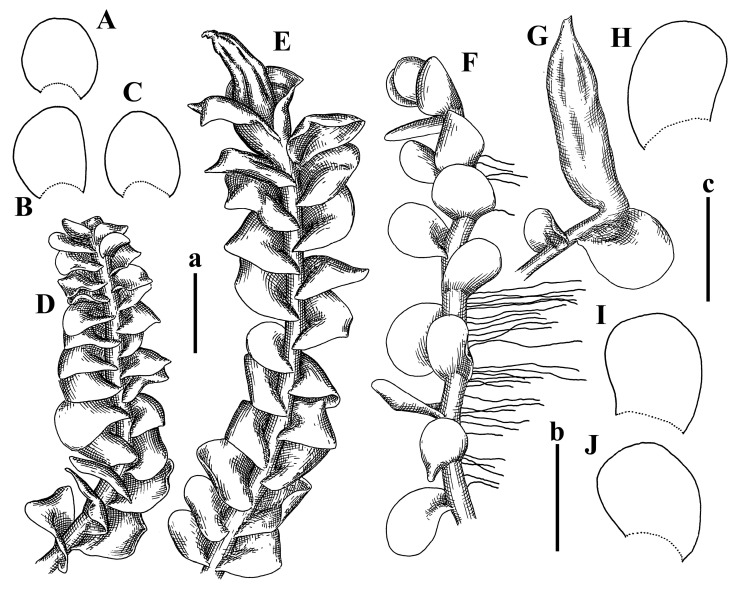
*Jungermannia plagiochilacea* Grolle Holotypus, NICH–73009: (**A**–**C**) leaves; (**D**) plant habit; (**E**) perianthous plant. *Jungermannia poeltii* Amakawa Isotypus JE, s.n.: (**F**) plant habit; (**G**) perianthous plant fragment; (**H**–**J**) leaves. Scales: a—2 mm for (**A**–**E**); b—1 mm for (**F**,**G**); c—500 µm for (**H**–**J**).

**Figure 30:**
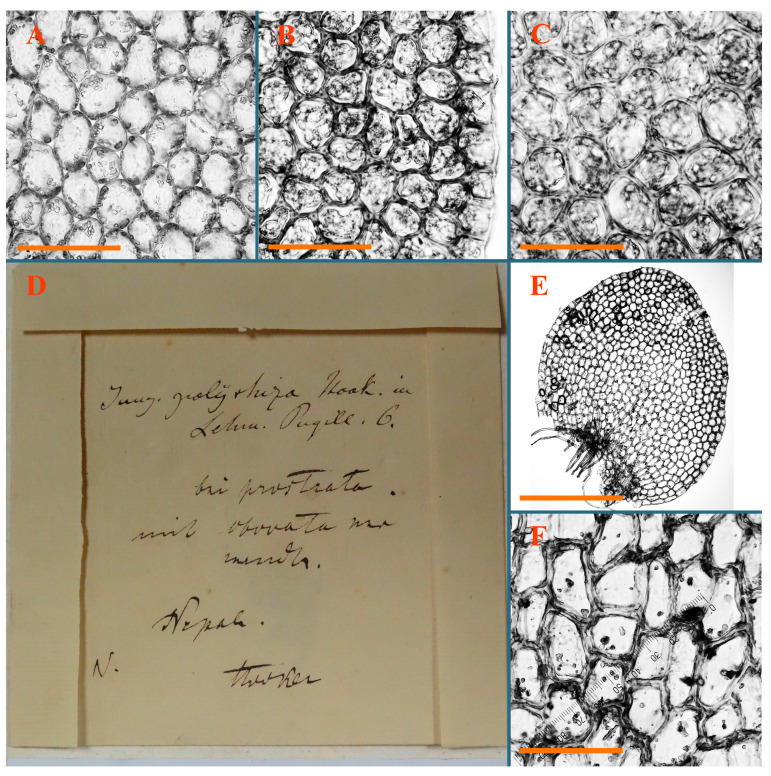
*Jungermannia plagiochilacea* Grolle Holotypus, NICH–73009: (**A**) midleaf cells. *Jungermannia poeltii* Amakawa Isotypus JE, s.n.: (**B**) leaf margin cells; (**C**) midleaf cells. *Jungermannia polyrhiza* Hook. ex Lehm. & Lindenb. Isotypus, STR, s.n.: (**D**) isotype label. *Jungermannia polyrhizoides* Holotypus, JE–04009139: (**E**) leaf; (**F**) midleaf cells. Scales: 100 µm for (**A**,**F**); 500 µm for (**B**,**C**); 300 µm for (**E**).

Comment 1: This species is perfectly described by Amakawa [8] and is characterized by a very distinct ‘pligiochiloid’ appearance due to plagiochiloid leaf insertion (subhorizontal in dorsal side, transverse in lateral side, and then again subhorizontal ventrally). Other distinctive characteristics are its more or less rigid texture and deep purple rhizoids, united into loose obliquely spreading fascicles or decurrent down the stem and forming obscure (although sometimes clear) fascicles (Figure 27E, Figure 29A–E and Figure 30A).

Comment 2: This is a very distinct species due to plagiochiloid leaf insertion (subhorizontal in dorsal side, transverse in lateral side and then again subhorizontal ventrally) that results in a Plagiochila-like appearance.

*Jungermannia poeltii* Amakawa, J. Hattori Bot. Lab. 29: 258, 1966.

Type: Nepal, Vorhimalaya, Okhaldunga, *Abies-Rhododendron*-Wald, um Thodung, Serting, 3250 m a.s.l., am Block; 1962, Poelt H108 (Holotypus NICH276487! Isotypus JE, s.n.!).

Accepted name: *Solenostoma poeltii* (Amakawa) Váňa et D.G.Long, Nova Hedwigia 89 (3/4): 505, 2009.

**Figure 31:**
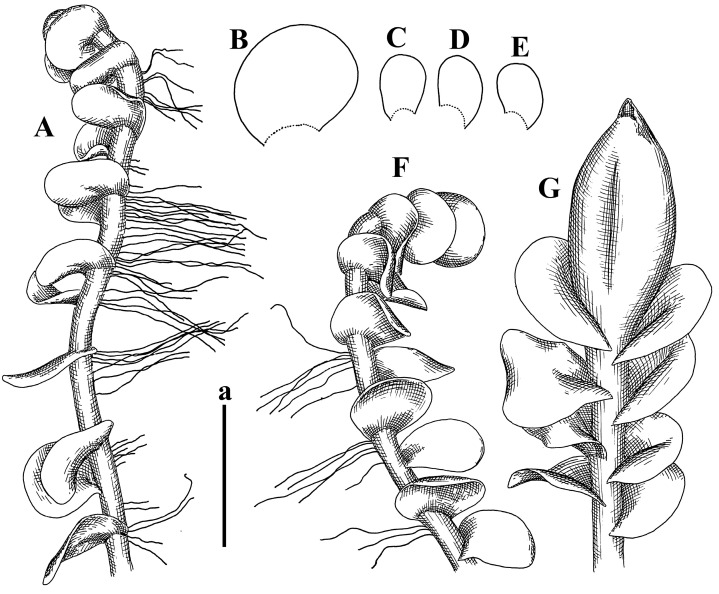
*Jungermannia poeltii* Amakawa Isotypus JE, s.n.: (**A**) plant habit; Holotypus, NICH–276487: (**B**) female bract; (**C**–**E**) leaves; (**F**) plant habit; (**G**) perianthous plant fragment. Scale: a—1 mm for (**A**–**G**).

Description: Plants prostrate, brownish to brown, (0.5)0.8–1.5 mm wide, more or less rigid, forming loose patches. Stem 100–180 µm in diameter, brownish to brown, branching lateral, rarely occurring, sometimes with 1–2 lateral subfloral innovations. Rhizoids sparse to numerous, erect spreading, mostly separated, but also united into unclear fascicles, colorless to grayish, sometimes near perianth with violet tint. Leaves distant, subtransversely inserted, not decurrent or up to 1/3 of stem width decurrent in both sides, mostly loosely sheathing the stem near base and obliquely to erect spreading and oriented above, more or less flattened in the general extent, but with recurved apex, in the slide mostly obliquely obovate ca. 570 × 450 µm, widest in upper 1/3, to (in perianthous shoots and near to perianth) ca. 1000 × 1200 µm widely obliquely obovate, widest near middle. Midleaf cells 12.5–22.5 × 12.5–20.0 µm, more or less thin-walled, walls brownish, with moderate-in-size to large, slightly concave or convex trigones, with visible (rust-colored) median lamina, cuticle smooth; cells along margin 12.5–17.5 µm, obscurely thickened, with external wall thickened, trigones moderate in size, concave. Possibly dioicous. Perianth 1600–1800 × 700–800 µm, obovate to subclavate, exerted for 3/4–4/4 of its length, 3–4-plicate (sometimes very loosely so) in upper 1/4 of its length, suddenly contracted to the beaked mouth, composed of subisodiametric to shortly oblong cells with thin to slightly thickened brown-colored walls and with moderate in size, mostly convex trigones, with visible median lamina, 2–3–stratose for 3/4 of its length; perigynium virtually absent; female bracts obliquely spreading, loosely sheathing perianth just near base and slightly concave–canaliculate above (Figure 29F–J, Figure 30B,C and Figure 31A–G).

Comment: Plants are very distinct due to rigid nature, and absence of red or purple pigmentation, and especially due to recurved obovate and loosely falcate leaves.

*Jungermannia polyrhiza* Hook. Ex Lehm. & Lindenb. Nov. Stirp. Pug. 6: 34, 1834.

Type: Nepal (Isotypus STR, s.n.!).

Accepted name: *Plectocolea comata* S. Hatt. Bull. Tokyo Sci. Mus. 11: 38, 1944.

**Figure 32:**
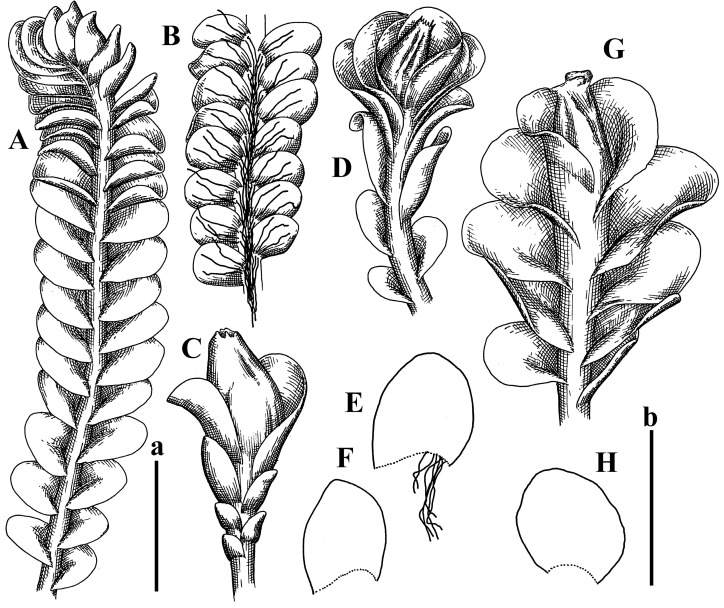
*Jungermannia polyrhiza* Hook. ex Lehm. & Lindenb. Isotypus, STR, s.n.: (**A**) plant habit; (**B**) plant habit ventral view. *Jungermannia polyrhizoides* Grolle ex Amakawa Isotypus, HIRO, s.n.: (**C**,**D**) perianthous plants fragments; (**E**,**F**) leaves; Holotypus, JE–04009139: (**G**) perianthous plant fragment; (**H**) leaf. Scales: a—3 mm for (**A**,**B**); b—1 mm for (**C**–**H**).

Comment: Although traditionally, *P. polyrhiza* is treated as the synonym of *P. truncata* (cf. [26]), the isotype of the taxon contains plants nearly identical to *P. comata* due to the (a) concentration of rhizoids origin near ventral leaf base; (b) unclear rhizoid fascicle decurrent down the stem, and many rhizoids obliquely spreading, with some decurrent upward along ventral leaf surface; and (c) pale-colored plants (including rhizoids). The only differentiating feature from common phases of P. comate are weakly developed leaf cuticle papillae (although papillae are present throughout, but not so high, as in typical P. comata; the latter feature may be due to the old specimen). Its identity with the holotype may be questioned; unfortunately, it was not studied (Figure 30D and Figure 32A,B).

*Jungermannia polyrhizoides* Grolle ex Amakawa, J. Hattori Bot. Lab. 29: 262, 1966.

Type: Nepal, O-Nepal, Vorhimalaja, Tutkosital bei Sorsale; 1962, Poelt H145 (Holotypus JE04009139!; Isotypus HIRO, s.n.!).

Accepted name: *Plectocolea polyrhizoides* (Grolle ex Amakawa) Bakalin & S.S. Choi Diversity 15(241): 11. 2023 (=*Solenostoma polyrhizoides* (Grolle ex Amakawa) Váňa et D.G.Long, Nova Hedwigia 89 (3/4): 505, 2009).

**Figure 33:**
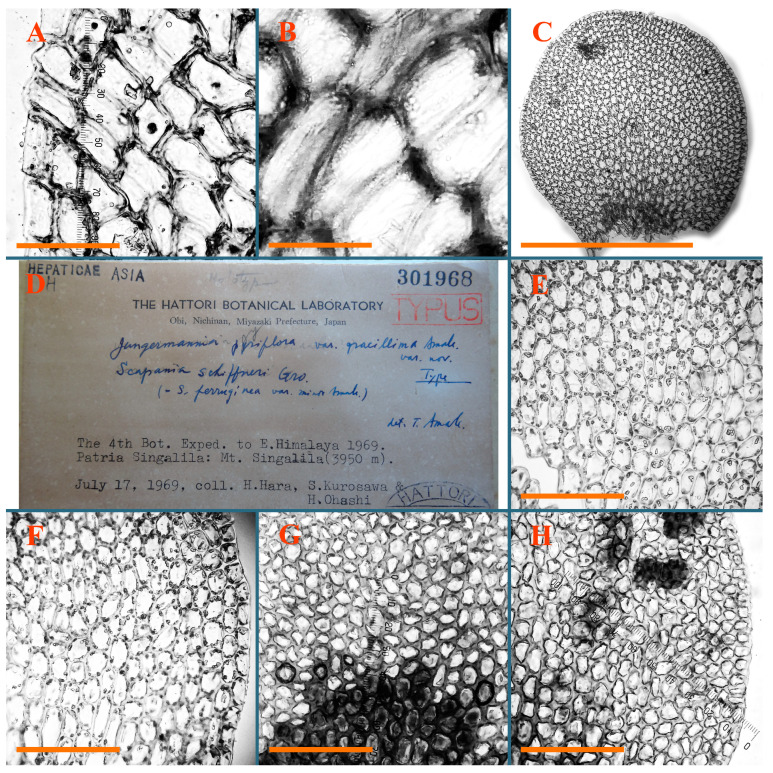
*Jungermannia polyrhizoides* Grolle ex Amakawa Holotypus, JE–04009139: (**A**) leaf margin cells; (**B**) basal cells showing papillae. *Jungermannia pyriflora* var. *gracillima* Amakawa Holotypus, NICH–301968: (**C**) leaf; (**D**) holotype label; (**E**) midleaf cells; (**F**) leaf margin cells. *Jungermannia raujeana* Grolle ex Amakawa Isotypus, HIRO, s.n.: (**G**) midleaf cells; (**H**) marginal cells. Scales: 100 µm for (**A**,**B**,**E**–**H**); 500 µm for (**C**).

Description: Plants prostrate, soft, lax, pellucid, greenish to pale yellowish greenish, 0.5 (sterile plants)–1.3 mm wide. Stem 100–250 µm in diameter, brownish, not branched, even as subfloral innovations. Rhizoids very dense, purplish (never bright purple), obliquely to erect spreading, separated or united into unclear fascicles, forming dense mat under stem and closely attaching plants one to another or to the substratum. Leaves distant to contiguous, obliquely to very obliquely inserted and oriented, not or barely decurrent dorsally and subtransversely inserted and not decurrent ventrally, nearly plane, obliquely spreading, near perianth subtransversely inserted and concave–canaliculate, obliquely spreading or sheathing the stem, ovate, 600–700 × 500–700 µm (rarely smaller or bigger). Midleaf cells thin-walled, 30–55 × 30–45 µm, with very small to vestigial, concave trigones, cuticle smooth; cells along leaf margin 30–60 µm, thin-walled, with small concave trigones, cuticle smooth or very loosely striolate. Perianth conical, exerted for 2/3 of its length, composed of elongated cells, brownish, 1000–1200 × 600–700 µm; perigynium ca. 1/3 of perianth length, with 2 pairs of bracts; female bracts closely sheathing perianth in lower half and concave–canaliculate and obliquely spreading above (Figure 30E,F, Figure 32C–F and Figure 33A,B).

Comment: The species is probably closely related to *P. granulata* (but not to “infusca group”, cf. [6]), from which it differs in its smooth or slightly papillose cuticle and dense, soft and only purplish rhizoids. The plants from the holotype in JE-04009139 (which, however, are also marked as an isotype(!)) are robust with a better developed striolate cuticle, but never with coarse ellipsoidal papillae as in *P. granulata*.

*Jungermannia pseudocyclops* Inoue, Bull. Natl. Sci. Mus. Tokyo (n.ser.) 9 (1): 37, 1966 (Inoue 1966a).

Type: Taiwan, Nan tow Hsien, Kuan-kao—Pa-tung-Kuan, ca 2700 m a.s.l.; 28 March 1963, S. Nakanishi 13,792 (Holotypus TNS-174431!).

Accepted name: *Solenostoma pseudocyclops* (Inoue) Váňa et D.G. Long, Nova Hedwigia 89 (3/4): 506, 2009.

**Figure 34:**
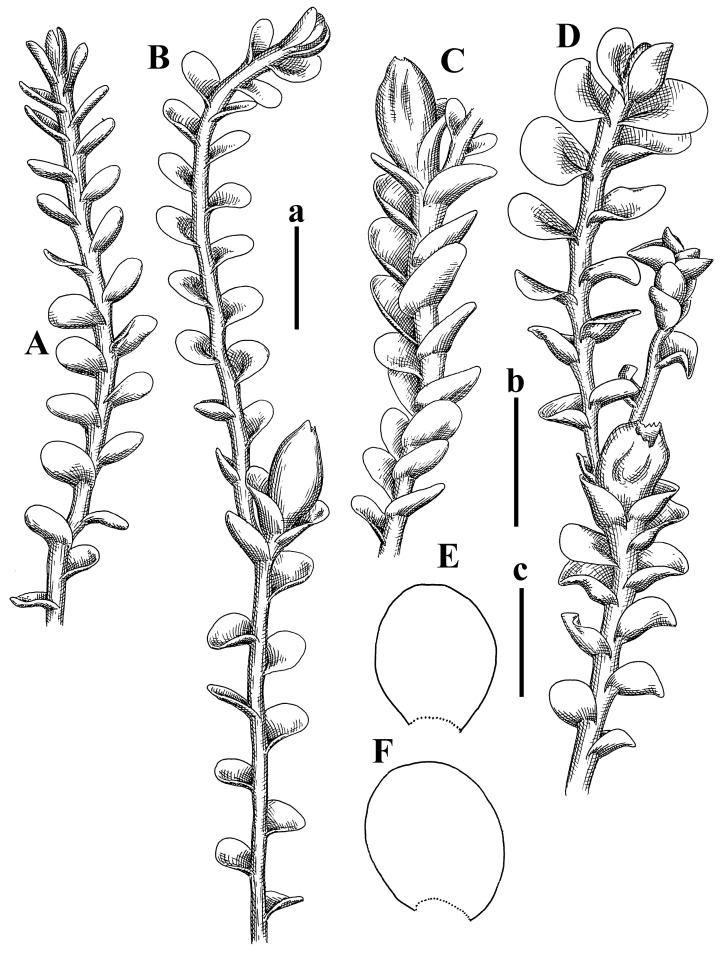
*Jungermannia pseudocyclops* Inoue Holotypus, TNS–174431: (**A**) plant habit ventral view; (**B**,**C**) perianthous plants. *Jungermannia pyriflora* var. *gracillima* Amakawa Holotypus, NICH–301968: (**D**) perianthous plant; (**E**,**F**) leaves. Scales: a—1 mm for (**A**–**C**); b—1 mm for (**D**); c—500 µm for (**E**,**F**).

Description: Plants filiform (as in other members of ‘*Nematocaulon* group’), brownish to yellowish brown, ascending (rarely creeping), 500–875 µm wide (near perianth up to 1.375 µm wide) and 5–10 mm long. Stem 125–225 µm in diameter, sparsely laterally (appeared near ventral leaf base) branched. Rhizoids not numerous, colorless to brown, obliquely to erect spreading. Leaves distant, concave to canaliculate–concave, shortly sheathing the stem near base and obliquely to erect spreading above, subtransversely inserted (ca. 50–70° with axis), dorsally shortly (up 1/4 of stem width) decurrent, ventrally transversely or arcuately inserted, orbicular to widely ovate, 400–700 × 450–770 µm. Midleaf cells isodiametric, 20–25 × 20–25 µm, thin-walled, walls brownish to yellowish brownish, trigones large, convex, cuticle papillose; along margin 12.5–22.5 µm, thin-walled, with large, convex to concave, sometimes confluent in tangential walls trigones, external wall noticeably thickened, cuticle sparsely verrucose; basal cells 22.5–42.5 × 20–25 µm, thin-walled, with moderate-in-size to large convex trigones, walls brownish, cuticle distinctly papillose. Dioicous. Perianth terminal, clavate, with 3(4) main plicae in upper 1/3, gradually narrowing to indistinctly beaked mouth, exerted for 3/4–4/4 of its length, 1.5–1.75 × 0.6–0.65 mm; mouth crenulate, composed of elongated ca. 22.5–25 × 12.5 µm thick-walled cells; perianth upper part cells isodiametric, 15–20 µm in diameter, with moderate-in-size, convex trigones; cells in the middle part of the perianth shortly elongated to subisodiametric, 20–25 × 15–20 µm, thin-walled, with large convex trigones, cuticle loosely papillose; perianth lower part cells 50–90 × 20–25 µm, thick-walled, trigones moderate, concave, cuticle coarsely striolate (Figure 34A–C).

Comment: The shortly fusiform perianth with distanced leaves are distinctive in this species.

*Jungermannia pyriflora* var. *gracillima* Amakawa, Fl. E. Himalaya 2: 228, 1971.

Type: India. West Bengal, Singalila Mt., 3950 m a.s.l.; 17 July 1969, H. Hara, S. Kurosawa & S. Ohashi (Holotypus NICH-301968!).

Accepted name: *Solenostoma amakawanum* Bakalin et S.S. Choi nom. nov. pro *Jungermannia pyriflora* var. *gracillima* Amakawa, Fl. E. Himalaya 2: 228, 1971 (this description validates the taxon) (=*Solenostoma pyriflorum* var. *gracillimum* (Amakawa) Váňa & D.G. Long)

**Figure 35:**
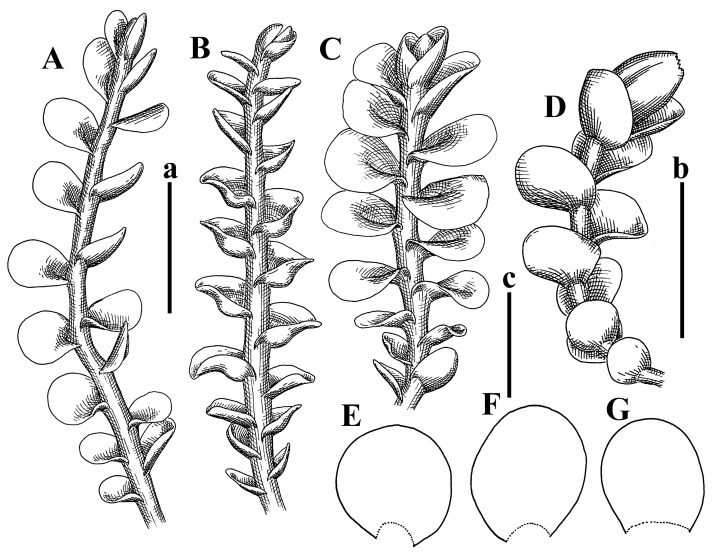
*Jungermannia pyriflora* var. *gracillima* Amakawa Holotypus, NICH–301968: (**A**,**C**) plant habit; (**B**) male plant. *Jungermannia raujeana* Grolle ex Amakawa Isotypus, HIRO, s.n.: (**D**) perianthous plant; (**E**–**G**) leaves. Scales: a—1 mm for (**A**–**C**); b—1 mm for (**D**); c—500 µm for (**E**–**G**).

Description: Plants ascending to nearly erect, 0.8–1.6 mm wide and 7–12 mm long, forming loose patches with *Scapania ciliatospinosa*, brownish greenish to yellowish brownish, rarely brown in the perianth and adjacent areas. Stem brownish to brown, 100–200 µm in diameter, not branched with the exception of subfloral innovations. Rhizoids sparse to common or virtually absent, obliquely spreading and united into unclear fascicles or decurrent down the stem and forming more or less distinct fascicles (in larger plants only), colorless to grayish. Leaves distant, subtransversely to transversely inserted, decurrent up to 1/2 of stem width in both sides, concave to concave–canaliculate or sheathing the stem near base and recurved above, ovate to nearly orbicular, 530–700 × 450–800 µm (1:0.9–1.1), erect spreading and transversely oriented. Midleaf cells subisodiametric to oblong, 17.5–30.0 × 15–17.5 µm, nearly thin-walled, trigones moderate to small, concave, cuticle smooth; cells along leaf margin 17.5–25.0 µm, thin-walled, external wall slightly thickened, trigones moderate in size, triangle to concave or slightly convex. Dioicous. Androecia intercalary, with 4–6 pairs of bracts, 1–2-androus, bracts inflate in lower half and recurved along margin in upper 1/3, similar in size with leaves, body nearly spherical, ca.100–110 µm in diameter, stalk biseriate, more or less short. Perianth exerted for 2/3 of its length, nearly obovate in outline, 3--plicate in upper 1/3, gradually or more or less suddenly contracted to the beaked mouth, composed of subisodiametric cells, bistratose in lower 2/3; perigynium virtually absent, bracts slightly inflate in lower 1/3 and concave–canaliculate and obliquely spreading above (Figure 33C–F, Figure 34D–F and Figure 35A–C).

Comment: The taxon is distinct due to leaves loosely sheathing the stem in the base, being nearly plane above; it has distanced leaves and a distinct rhizoid fascicle decurrent down along the ventral side of the stem in well-developed plants.

*Jungermannia raujeana* Grolle ex Amakawa, J. Hattori Bot. Lab. 29: 262, 1966.

Type: Ost-Nepal, Vorhimalaja, alpine Matten, Rauje ostseitige, Steilflashe eines Blockes, 4500 m a.s.l.; 1962 Poelt H136 p.p. (Isotypus HIRO, s.n.!; holotypus was not located in JE).

Accepted name: *Solenostoma raujeanum* (Grolle ex Amakawa) Váňa et D.G.Long, Nova Hedwigia 89 (3/4): 507, 2009.

**Figure 36:**
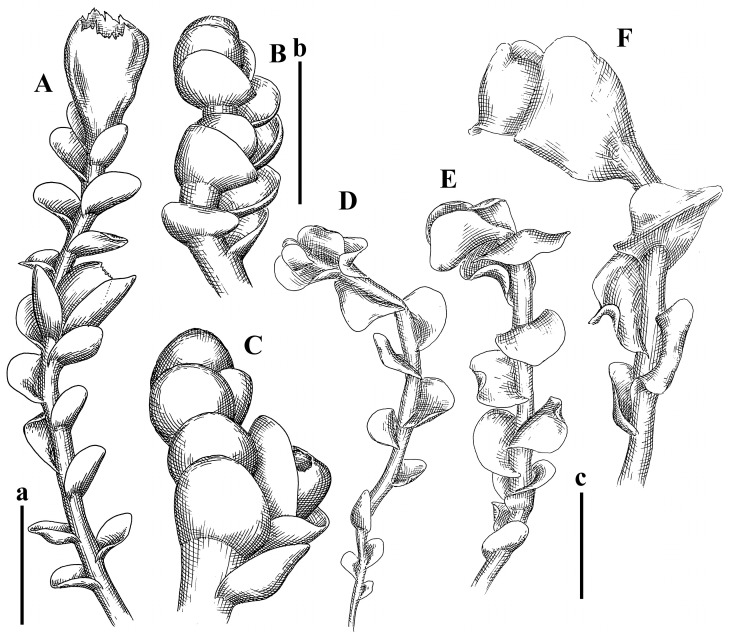
*Jungermannia raujeana* Grolle ex Amakawa Isotypus, HIRO, s.n.: (**A**) perianthous plant. *Jungermannia saccaticoncava* Amakawa Holotypus, NICH–312380: (**B**) plant habit; (**C**) perianthous plant. *Jungermannia scalariformis* Nees Holotypus, STR, s.n.: (**D**,**E**) plant habit; (**F**) plant with gynoecia. Scales: a—1 mm for (**A**); b—1 mm for (**B**,**C**); c—2 mm for (**D**,**F**).

Description: Plants rather erect, brown to rusty brown and blackish brown, (0.2–)0.5–0.9 mm wide and 5–7 mm long. Stem 70–120 µm in diameter, sparsely laterally branched, also with lateral subfloral innovations, blackish brown. Rhizoids rather sparse, colorless to brownish, obliquely to erect spreading. Leaves nearly transversely inserted, obliquely spreading, decurrent for 1–1.5 stem width in both sides, slightly concave to concave–canaliculate, ovate to suborbicular, 440–560 × 440–660 µm, rather distant. Midleaf cells subisodiametric, 10–20 × 10–18 µm, nearly thin-walled, with large and sometimes confluent, concave to convex trigones, cuticle distinctly papillose; cells along leaf margin 8–12 µm, thin-walled to strongly unequally thickened due to trigones’ confluence, external wall thickened, trigones large, slightly concave to convex, sometimes confluent, cuticle papillose, margin commonly discolored. Possible dioicous. Perianth ca. 1100 × 750 µm, obovate to obcuneate, nearly smooth or slightly 4-plicate in upper 1/5–1/4, where it gradually or more or less suddenly narrows to obscurely beaked mouth; composed of subisodiametric cells, 2–3-stratose in lower 2/3 (Figure 33G,H, Figure 35D–G and Figure 36A).

Comment: The species may be recognized by its rigid texture (probably growing in a strongly xerophytic habitat), dark color, small leaf cells with large trigones, and deeply colored leaf in its middle-basal portion.

*Jungermannia saccaticoncava* Amakawa Flora of Eastern Himalaya 3: 218, f. 26. 1975.

Type: East Nepal, between Thang La and Thudam, soil on rock, 4550 m a.s.l.; 21 June 1972, Z. Iwatsuki 1461 (Holotypus NICH-312380!).

Accepted name: *Solenostoma macrocarpum* (Schiffn. ex Steph.) Váňa et D.G.Long, Nova Hedwigia 89 (3/4): 504, 2009.

**Figure 37:**
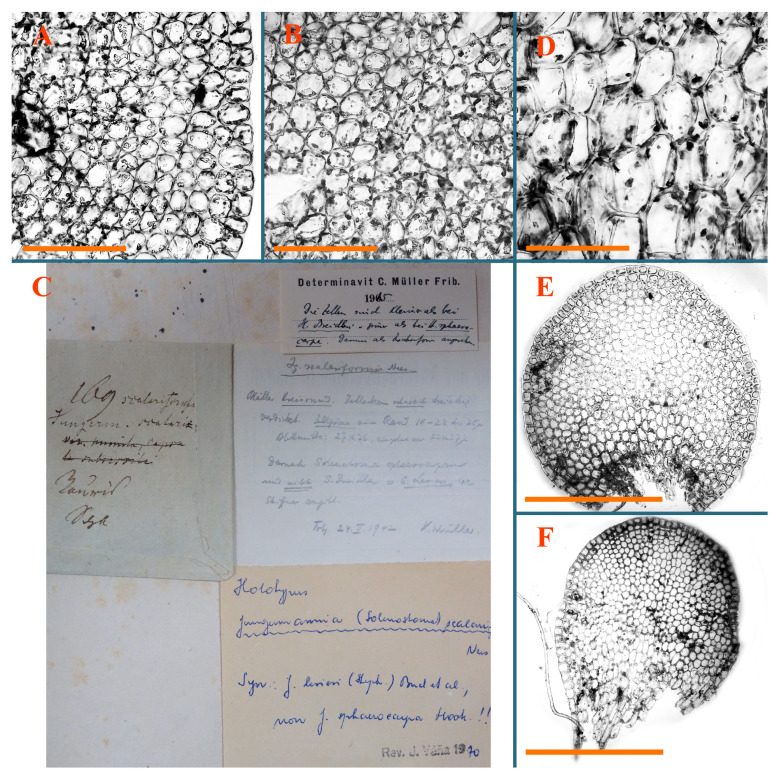
*Jungermannia saccaticoncava* Amakawa Holotypus, NICH–312380: (**A**) leaf margin cells; (**B**) midleaf cells. *Jungermannia scalariformis* Nees Holotypus, STR, s.n.: (**C**) holotype label. *Jungermannia shimizuana* S.Hatt. ex Váňa Paratypus, NICH–56399: (**D**) midleaf cells. *Jungermannia suborbiculata* Amakawa Holotypus, NICH–241976: (**E**,**F**) leaves. Scales: 100 µm for (**A**,**B**,**D**); 500 µm for (**E**,**F**).

Description: Plants prostrate to loosely ascending, closely attached to the substratum by the rhizoids, yellowish brownish, with purplish or reddish tint near apices of some shoots, forming dense patches encrusted by soil particles, 0.6–1.0 mm wide and 3–5 mm long. Stem 100–170 µm in diameter, not branched, with the exception of lateral subfloral innovations. Rhizoids numerous, dense, erect to obliquely spreading, sometimes united into unclear fascicles, closely attaching plant to the substratum, colorless to grayish and brownish. Leaves subtransversely inserted (near ventral base, transversely), not decurrent in both sides, sub- to imbricate, rarely contiguous, concave to almost cupped and lacerating, when flattened in the slide, nearly orbicular, 600–800 × 900–1200 µm, obliquely spreading. Midleaf cells nearly subisodiametric 20–25 × 15–25 µm, thin-walled, with moderate-in-size, slightly convex trigones, cuticle smooth; cells along leaf margin 15–25 µm, nearly thin-walled, with thickened external wall, trigones large, rarely confluent, cuticle smooth. Possibly dioicous. Perianth obovate, 600–800 × 500–600 µm, exerted for 1/3–1/2 of its length, loosely 3-plicate in upper half, suddenly contracted to the distinctly beaked mouth, with 1 lateral subfloral innovation per perianth that immediately becomes large, such that the perianth looks to be situated on the lateral short branch, but not on the main axis; composed of subisodiametric cells with large and convex trigones, bistratose in lower 2/3; perigynium virtually absent; female bracts concave, covering the perianth, similar to large leaves (Figure 36B,C and Figure 37A,B).

Comment: Although we agree with the the synonymization of this name with *Solenostoma macrocarpum* [26], *Jungermannia saccaticoncava* differs from the former in its smaller plant size, shorter perianth, and the presence of purplish pigmentation in androecial branches; these should be evaluated in further studies.

*Jungermannia scalariformis* Nees Naturgeschichte der Europäischen Lebermoose 2: 448. 1836.

Type: Austria, Salzburger Alpen; Funck (Holotypus STR, s.n.!).

Accepted name: *Solenostoma sclariforme* (Nees) Bakalin et S.S. Choi comb. nov. Basionym *Jungermannia scalariformis* Nees Naturgeschichte der Europäischen Lebermoose 2: 448. 1836. (Naturgesch. Eur. Leberm.) (=*Nardia levieri* Steph. Botanisches Centralblatt 50: 70. 1892.; =*Solenostoma levieri* (Steph.) Steph. Bulletin de l’Herbier Boissier, sér. 2, 1: 488. 1901.)

Description: Plants ascending, lax, gentle, pale yellowish brownish, without red pigmentation. Rhizoids numerous, obliquely spreading or decurrent down the stem in unclear fascicle. Leaves distant to merely contiguous, loosely concave, larger ones undulate at margin, obliquely inserted, transversely elliptic, 475–1000 × 600–1450 µm, lamina rhizogenous in well-developed plants. Midleaf cells 25.0–40.0 × 22.5–37.5 µm, thin-walled, trigones moderate in size to small, slightly concave to slightly convex. Paroicous. Androecia in 1(2) pairs of bracts, 1(2)-androus. Perianth nearly hidden in bracts to shortly exerted, 4–5-plicate, gradually narrowing to well-developed beak (Figure 36D–F and Figure 37C).

Comment: Váňa handwrote (Figure 37C) that this species is identical to *Solenostoma levieri* (Steph.) Steph., but not *S. sphaerocarpum*, as was indicated by Müller (Figure 37C). Later, Váňa [2] synonymized *S. levierii* with *S. confertissimum*; however, we guess the two species are quite different, as also indicated by Bakalin [1]. The present species also shows distinct features in common with the *S. levierii* type, like an only shortly exerted perianth and an absence of red pigmentation. The taxonomic value of these distinctions is difficult evaluate now.

*Jungermannia shimizuana* S.Hatt. ex Váňa, J. Hattori Bot. Lab. 35: 315, 1972.

Type: Japan, Saitama Prefecture, Chichibu Mts., Mt. Kobushi, 2300–2400 m a.s.l.; 24 August 1952, D. Shimizu s.n. (Holotypus NICH58537!; Paratypus from the same place NICH56399!).

Accepted name: *Solenostoma shimizuanum* (S.Hatt. ex Váňa) Váňa, Hentschel et Heinrichs, Cryptog. Bryol. 31 (2): 138, 2010.

**Figure 38:**
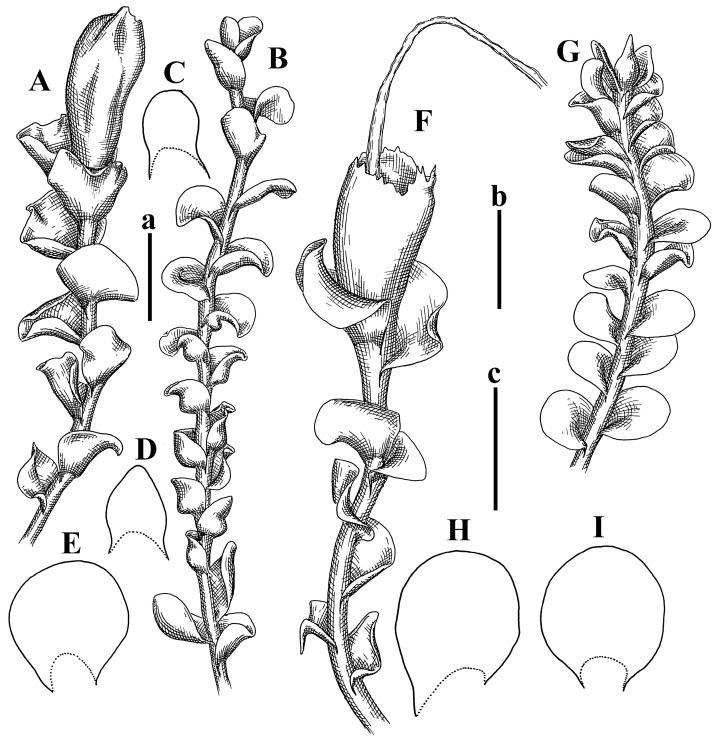
*Jungermannia shimizuana* S.Hatt. ex Váňa Holotypus, NICH–58537: (**A**) perianthous plant; (**B**) male plant, (**C**–**E**) leaves. *Jungermannia suborbiculata* Amakawa Holotypus, NICH–241976: (**F**) perianthous plant; (**G**) plant habit; (**H**,**I**) leaves. Scales: a—1 mm for (**A**–**E**); b—1 mm for (**F**,**G**); c—1 mm for (**H**,**I**).

Description: Plants brownish, sometimes with purplish tint in upper part of the perianth and inflated area of male bracts, 0.8–1.6 mm wide and 8–17 mm long. Stem 90–120 µm in diameter, brownish to brown, not branched, even as subfloral innovations. Rhizoids colorless to grayish, rarely with purplish tint, decurrent down the stem and forming distinct fascicle. Leaves lax, contiguous to distant, subtransversely (fertile branches) to very obliquely (sterile branches) inserted, dorsally decurrent for 1–3 stem width, ventrally arcuately inserted, decurrent for 1–3 stem width, obliquely spreading, concave–canaliculate to nearly flattened, mostly undulate along margin, commonly deflexed away of the shoot apex, 650–1200 × 650–1500, ovate to rounded and transversely elliptic. Midleaf cells thin-walled, oblong, 50–67 × 35–43 µm, trigones small, concave, cuticle nearly smooth; cells along leaf margin strongly collapsed, therefore difficult to see. Dioicous. Androecia with 4–5 pairs of bracts; male bracts strongly inflated in lower half and concave–canaliculate and obliquely spreading above. Perianth 1500–2300 × 600–1000 µm, exerted for 3/4 of its length, loosely 3-plicate in upper half, gradually to more or less distinctly contracted to not or obscurely beaked mouth, composed of subisodiametric cells, slightly rhizogenous in lower half (lateral and dorsal sides); rhizoids originating from perianth sometimes with purplish tint, bistratose in lower 2/3; perigynium virtually absent, bracts similar to large leaves (Figure 37D and Figure 38A–E).

Comment: The material in the holotype similar to the paratype, but more lax, without purplish and reddish pigmentation.

*Jungermannia suborbiculata* Amakawa, J. Hattori Bot. Lab. 31: 112, 1968.

Type: India, Darjeeling, Sandaphu (3550 m a.s.l.)—Kala Pokri (3000 m a.s.l.)—Caribans (2600 m a.s.l.)—Tonglu (3000 m a.s.l.); 7 June 1960, H. Hara et al. s.n. (Holotypus NICH241976!).

Accepted name: *Solenostoma suborbiculatum* (Amakawa) Váňa et D.G.Long, Nova Hedwigia 89 (3/4): 508, 2009.

**Figure 39:**
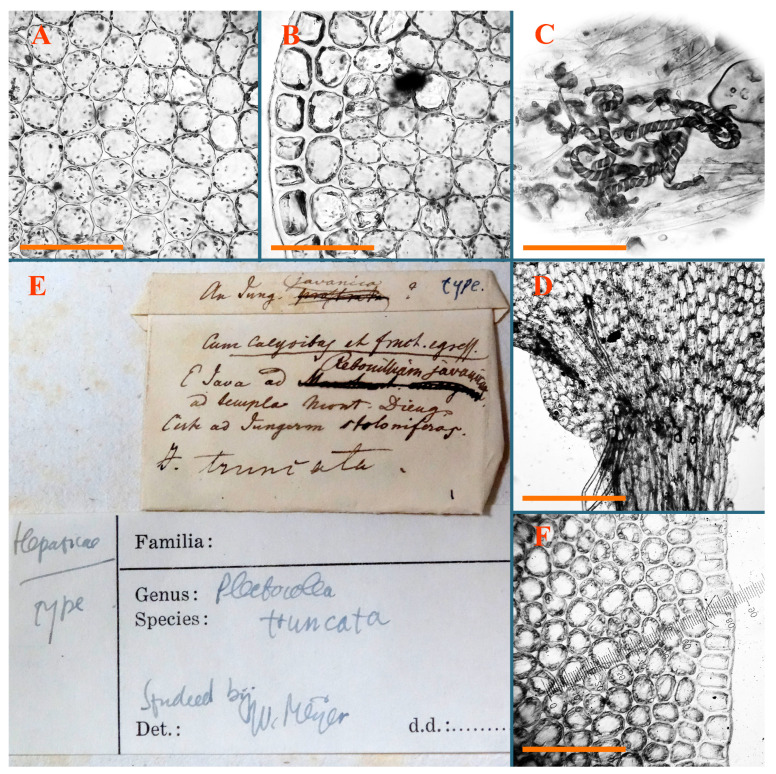
*Jungermannia suborbiculata* Amakawa Holotypus, NICH–241976: (**A**) midleaf cells; (**B**) marginal cells; (**C**) elaters. *Jungermannia tetragona* Lindenb. Isotypus, PC0102912: (**D**) rhizogenous cells in leaf base. *Jungermannia truncata* Nees Holotypus, STR, s.n.: (**E**) holotype label; (**F**) marginal cells. Scales: 100 µm for (**A**–**C**,**F**); 300 µm for (**D**).

Description: Plants ascending to erect, brownish, sometimes with light purple tint, 1.2–1.7 mm wide (near perianth up to 2.8 mm), 8–20 mm long, forming loose mats with other hepatics. Stem 200–300 µm in diameter, brownish, not branched, even as subfloral innovations. Rhizoids numerous to sparse, colorless, obliquely spreading and forming mat under stem, or decurrent down the stem, but not forming distinct fascicle. Leaves subtransversely to transversely inserted (in lower part of shoot or in depauperate branches, obliquely inserted), decurrent up to 1 stem width in both sides, concave to concave–canaliculate, sometimes loosely appressed to the stem laterally, but mostly obliquely spreading and subtransversely oriented, distant to contiguous and subimbricate, 700–1100 × 800–1200 µm, nearly orbicular. Midleaf cells subisodiametric, 32–50 × 33–45 µm, thin-walled, trigones moderate in size, convex, cuticle smooth; cells along leaf margin 30–48 µm, strongly unequally thick-walled, trigones large, sometimes confluent, mostly convex, cuticle smooth, the next row inward commonly with slightly thickened cell walls. Dioicous. Androecia intercalary, with 2–3 pairs of bracts, male bracts similar to leaves, but inflate in lower half and commonly with purplish tint in inflated area. Perianth 1700–2200 × 1100–1300 µm, obovate, suddenly contracted to loosely beaked mouth, loosely 3–4-plicate in upper half or almost smooth, exerted for 3/4 of its length or even more, composed of nearly subisodiametric or shortly oblong cells, densely rhizogenous, unistratose in upper 1/3, bistratose near middle and 3–4-stratose in loser third; perigynium 1/4–1/3 of perianth length, with 1 pair of bracts; female bracts similar to leaves, sheathing perianth near base and obliquely spreading above or obliquely spreading starting from the base. Spores brown, papillose, 20–25 µm in diameter. Elaters bispiral, ca. 150 × 10, with long homogenous ends, ca 20 µm long in each side (Figure 37E,F and Figure 39A–C).

Comment: This is a distinct species characterized by strongly thick-walled cells along the leaf margin, convex trigones, and sub- to transversely inserted leaves.

*Jungermannia tetragona* Lindenb., Bot. Zeitung (Berlin) 6 (25): 462, 1848.

Type: Java; Zollinger 1581b (Isotypus PC0102912!).

Accepted name: *Plectocolea tetragona* (Lindenb.) Amakawa Journal of Japanese Botany 33: 144. 1958. (J. Jap. Bot.) (=*Solenostoma tetragonum* (Lindenb.) R.M.Schust. ex Váňa et D.G.Long, Nova Hedwigia 89 (3/4): 509, 2009).

**Figure 40:**
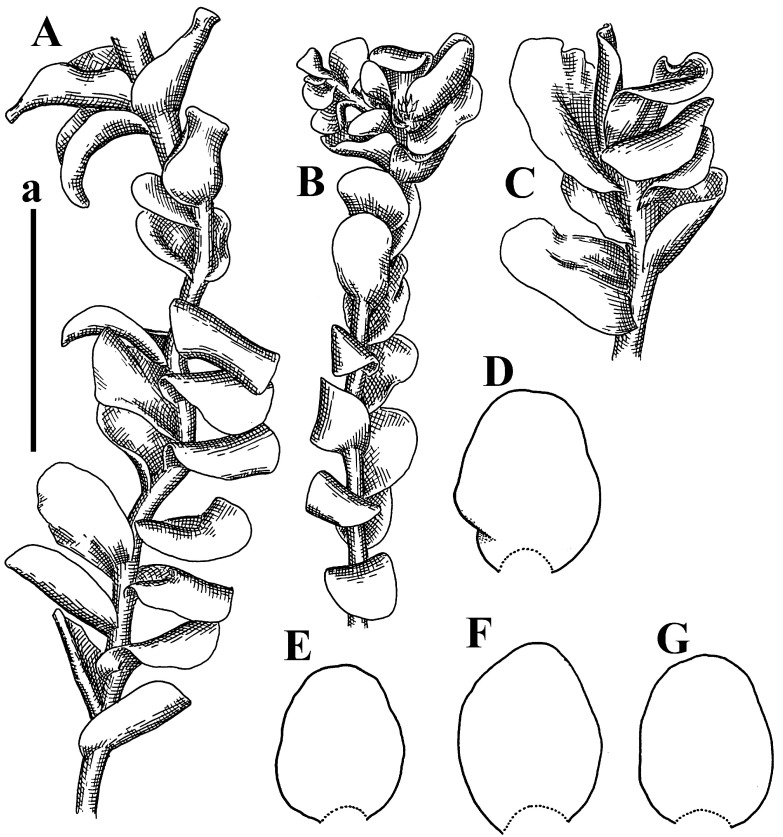
*Jungermannia tetragona* Lindenb. Isotypus, PC0102912: (**A**) plant habit; (**B**,**C**) plant with gynoecia; (**D**–**G**) leaves. Scale: a—3 mm for (**A**–**G**).

Description: Plants brown in the herbarium, erect to ascending, 1500–3000 µm wide and 15–20 mm long, more or less rigid. Rhizoids few to numerous, decurrent down the stem in loose fascicles or obliquely spreading, separated, deep purple in color, rigid, originating mostly near ventral leaf base, rarer in other part of ventral side of stem and solitarily in lower half of the leaf lamina. Leaves obliquely inserted, dorsally and ventrally shortly (to 1/3–1/2 of stem width) decurrent, obliquely spreading, transversely oriented, slightly canaliculate–concave to almost plane, contiguous, ovate, larger sometimes loosely undulate along margin, 1120–1750 × 875–1500 µm. Midleaf cells oblong, 32.5–50.0 × 25.0–32.5 µm, thin-walled, trigones moderate to small, concave, cuticle loosely papillose–striolate; 17.5–32.5 µm, nearly thin-walled, trigones small, concave. Dioicous. Perianth with 3–6 main plicae, plica ridge sometimes crispate, exerted for 2/3–3/4 of the length, nearly fusiform; perigynium less than 1/5 of perianth length; bracts similar to leaves, but wider, sheathing perianth in lower third and obliquely spreading above (Figure 39D and Figure 40).

Comment: This is an easily identified species due to its rhizogenous leaves, purple rigid rhizoids, and low perigynium.

*Jungermannia truncata* Nees, Enum. Pl. Crypt. Javae: 29, 1830.

Type: Java (Holotypus STR, s.n.!).

Accepted name: *Plectocolea truncata* (Nees) Herzog Transactions of the British Bryological Society 1: 281. 1950. (=*Solenostoma truncatum* (Nees) R.M.Schust. ex Váňa et D.G.Long, Nova Hedwigia 89 (3/4): 509, 2009).

**Figure 41:**
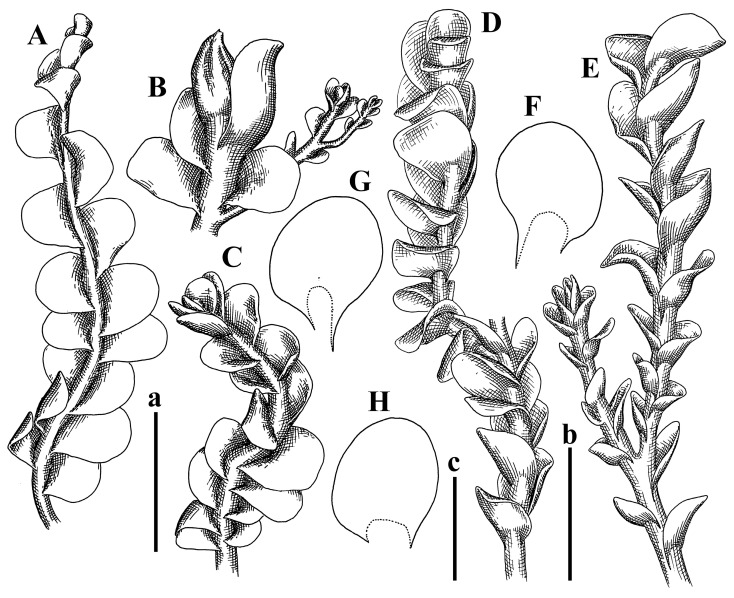
*Jungermannia truncata* Nees Holotypus, STR, s.n.: (**A**,**C**) plant habit; (**B**) perianthous plant fragment. *Jungermannia ventroversa* Grolle Isotypus, NICH–242611: (**D**,**E**) plant habit; (**F**–**H**) leaves. Scales: a—1 mm for (**A**–**C**); b—2 mm for (**D**,**E**); c—1 mm for (**F**–**H**).

**Figure 42:**
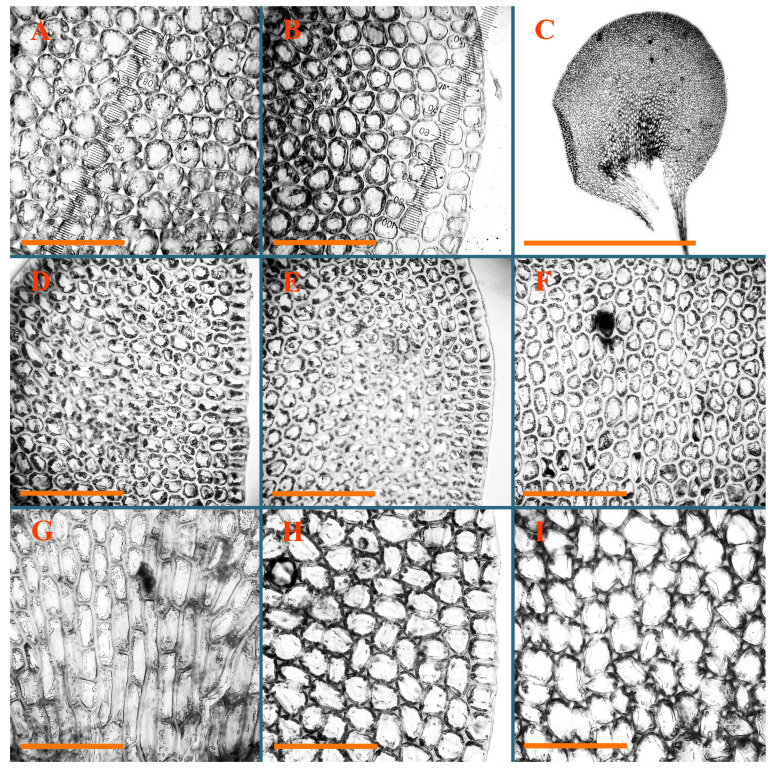
*Jungermannia truncata* Nees Holotypus, STR, s.n.: (**A**) midleaf cells; (**B**) leaf marginal cells. *Jungermannia ventroversa* Grolle Isotypus, NICH–242611: (**C**) leaf; (**D**) leaf margin cells; (**E**,**F**) midleaf cells; (**G**) leaf basal cells. *Jungermannia zantenii* Isotypus, NICH–242611: (**H**) leaf margin cells; (**I**) midleaf cells. Scales: 100 µm for (**A**,**B**,**D**–**I**); 1mm for (**C**).

Description: Plants soft, gentle, forming loose mats (as can be seen from the specimen), prostrate to loosely ascending near apices, pale brownish, 750–1000 µm wide and 5–8 mm long. Rhizoids dense, numerous, colorless to brownish or sometimes purple, originated along ventral side of stem, with concentration near ventral leaf bases. Leaves contiguous to subimbricate near apices, obliquely inserted, with insertion line in ventral side subtransverse, not or barely decurrent dorsally, 430–560 × 430–670 µm, reniform to widely obliquely ovate, slightly to evidently concave. Midleaf cells subisodiametric, 22.5–32.5 × 20–30 µm, thin-walled, trigones moderate in size, convex, cuticle papillose; cells along margin strongly subequally thickened, trigones small to moderate, concave, sometimes tangentially elongated, 12.5–30.0 µm, margin sometimes discolored (then looks as the rim). Dioicous. Perianth conical, loosely pluriplicate, exerted for 1/2–3.5 of the length, composed of elongated cells; pergynium distinct, ca. 1/2 of perianth length (Figure 39E,F, Figure 41A–C and Figure 42A,B).

Comment: This is one of the most common south-subtropical–tropical Asian taxa of the genus, easily recognizable by its thickened cell walls along the leaf margin, prostrate growth, and colorless to pinkish or rarely purple (only locally) rhizoids.

*Jungermannia ventroversa* Grolle, Khumbu Himal 1 (4): 284, 1966.

Type: Nepal, Ost-Nepal, Vorhimalaja, *Abies-Rhododendron*-Bergwald, alpine matten, ostlich uber Ringmo; 1962, Poelt H187 (Holotypus JE, s.n.!; Isotypus NICH242611!).

Accepted name: *Solenostoma ventroversum* (Grolle) Váňa et D.G.Long, Nova Hedwigia 89 (3/4): 510, 2009.

Description: Plants erect in rather dense patches, yellowish brownish, without traces of red pigmentation, rigid, 1.2–1.8 mm wide and 10–20 mm long. Stem 160–300 µm in diameter, more or less regularly laterally branched, brownish to brown. Rhizoids sparse to numerous, grayish to brownish, more or less rigid, obliquely spreading in unclear fascicles, not decurrent down the stem. Leaves loosely laterally appressed to the stem to obliquely spreading, concave–canaliculate with canal line commonly incurved to ventral side or loosely sheathing the stem near base and recurved above (thus, leaves seem convex); leaves ventrally secund, turned to ventral side (“ventroversa”), distant to contiguous, nearly transversely inserted, dorsally decurrent for 1.5–2.0 stem width, ventrally decurrent for 2–3 stem widths, dorsal side of leaf (in all well-developed leaves), narrowly or more broadly (3–4 cells rows) incurved, 800–1000 × 900–1200 µm, obliquely transversely elliptic to nearly orbicular, near central part of the leaf base slightly inflated (as if they contain antheridia) and more deeply (up to brown) colored there. Midleaf cells subisodiametric to shortly oblong, unequally thickened, 12.5–25.0 × 12.5–17.5 µm, trigones moderate in size, concave, cuticle smooth or nearly so; cells along margin 10–20 µm, unequally thick-walled, with moderate-in-size, concave trigones, cuticle smooth; cells in inflated area of the leaf near base elongated, more or less equally thick-walled, 32–55 × 12–22 µm, cuticle striolate (sometimes loosely) (Figure 41D–H and Figure 42C–G).

Comment: The authors of the World liverwort and hornwort checklist [17] regard *Solenostoma ventroversum* as a taxon with serious doubt status (possibly conspecific with *S. appressifolium*, as written there). We cannot agree with this point of view; on the contrary, we believe this is to be a very good species. The unique feature of the taxon is its combination of unequally thickened cell walls in the mid- and leaf marginal cells, and peculiarly ventrad leaves.

*Jungermannia zantenii* Amakawa, J. Hattori Bot. Lab. 31: 110, 1968.

Type: Papua New Guinea. West Irian, Antares Mts., Camp 39a, 1500 m a.s.l., terrestrial and at the base of shrubs in rain forest border; 2 July 1959, B.O. van Zanten 426 (Holotypus NICH-234643!; Isotypus TNS-27140!).

Accepted name: *Solenostoma zantenii* (Amakawa) R.M.Schust. ex Váňa et D.G.Long, Nova Hedwigia 89 (3/4): 510, 2009.

**Figure 43:**
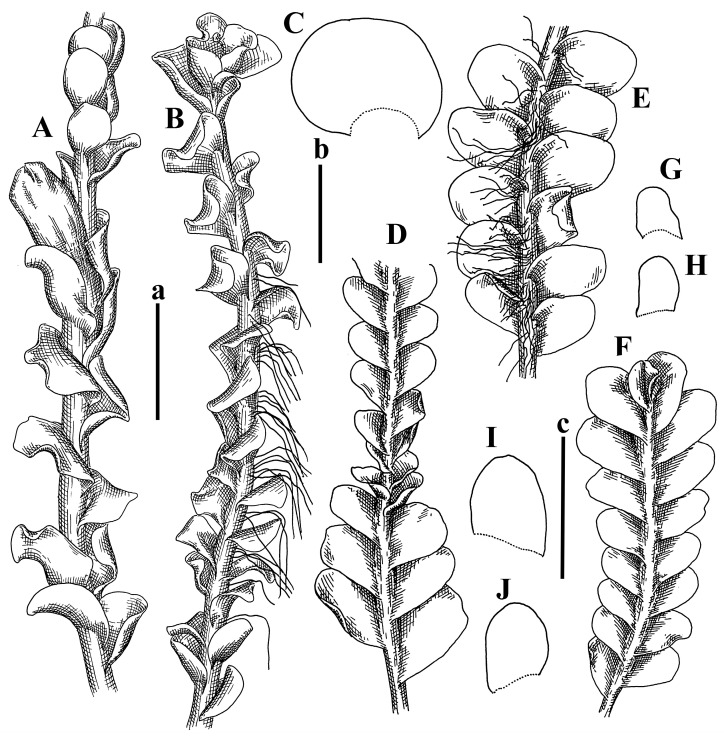
*Jungermannia zantenii* Amakawa Holotypus, NICH–23464: (**A**) perianthous plant; (**B**) plant habit; (**C**) leaf. *Nardia granulata* Steph. Holotypus, G–14763: (**D**,**F**) plant habit; (**E**) plant habit ventral view; (**G**–**J**) leaves. Scales: a—1 mm for (**A**,**B**); b—1 mm for (**C**); c—2 mm for (**D**–**J**).

Description: Plants rather erect in dense patches, more or less rigid, yellowish brown, without red or purplish pigmentation, 1.25–2.25 mm wide and 10–20 mm long. Stem 200–425 µm in diameter, not branched, with the exception of subfloral innovations. Rhizoids not numerous (rather sparse), erect to obliquely spreading, mostly separated or in unclear fascicles, brownish, more deeply colored than the stem, brownish to deep brownish. Leaves distant, rarely contiguous, obliquely to erect spreading, loosely sheathing the stem near base and recurved in upper half, obliquely inserted (ca. 30–45° with axis), strongly undulate at margin, loosely sheathing the stem near base and obliquely to erect spreading above, loosely canaliculate, commonly deflexed away the shoot apex, dorsally decurrent for 1.0–2.0 of stem width, ventrally arcuately inserted, shortly decurrent, obliquely ovate, 700–1025 × 700–1125 µm (~1:1). Midleaf cells subisodiametric, 25–45 × 25–38 µm, thin-walled, trigones moderate in size, convex, cuticle loosely papillose; cells along leaf margin 25–32 µm, thin-walled, with thickened external walls, trigones moderate in size, slightly convex, cuticle loosely papillose. Possibly dioicous. Perianth clavate to nearly fusiform, 3–4-plicate in upper 1/3, gradually narrowing to not or indistinctly beaked mouth, exerted for 2/3–1 of its length, ca. 2000 × 800 µm, mouth crenulate due to protrudent orbicular to shortly oblong, moderately thick-walled cells, 27.5–35 × 22.5–25.0 µm; cells in the perianth middle oblong, 45–75 × 20–25 µm, walls thin, yellowish brownish, trigones moderate to small, triangle to slightly convex, cuticle noticeably striolate; cells in the perianth base elongate, 55–105 × 15–23 µm, thin-walled, trigones small, concave, cuticle distinctly striolate; partly bistratose in lower 1/4. Perigynium virtually absent. Female bracts loosely sheathing the perianth to almost its full extent and recurved in upper 1/5–1/4 (Figure 42H,I and Figure 43A,C).

Comment: The species is morphologically similar to *Solenostoma hewsoniae*, as was noted by Amakawa [7]. Amakawa [7]: 110 differs *S. hewsoniae* differs in that the “plant is light green and is more slender, leaves are sinuately inserted on the stem and are canaliculately concave, leaf cells are larger (38–62 × 25–38 µm in the middle), and the perianth is long-exerted”. As it seems from the descriptions provided in the present account, these variation spans are largely overlapping, and further investigations comparing the two taxa are required.

*Nardia granulata* Steph. Bulletin de l’Herbier Boissier 5: 100. 1897.

Type: Japan, Ushioye-yama near Kochi; November 1895 T. Makino (Lectotypus designated here: G14763); at least two original specimens are known for this taxon, since Stephani returned part of the material to the sender, and kept only the duplicate in his herbarium (now in G). As this description is assumed based on the material in G, it is better to select it as a lectotype.

Accepted name: *Plectocolea granulata* (Steph.) Bakalin Polish Botanical Journal 58(1): 132. 2013.

**Figure 44:**
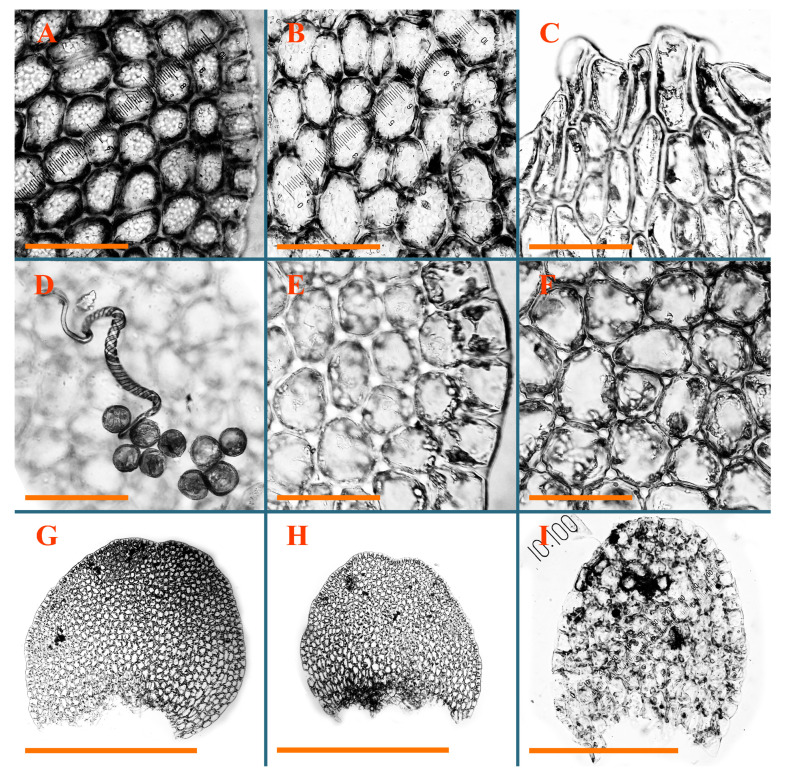
*Nardia granulata* Steph. Holotypus, G–14763: (**A**) marginal cells showing papillae; (**B**) midleaf cells. *Nardia paroica* Schiffn. Isotypus, PC–0102753: (**C**) perianth mouth; (**D**) spores and elater; (**E**) leaf margin cells; (**F**) midleaf cells; (**G**,**H**) leaves); *Nardia subtilissima* Schiffn. Isotypus, HIRO, s.n.: (**I**) leaf. Scales:100 µm for (**A**–**F**); 1 mm for (**G**,**H**); 200 µm for (**I**).

Description: Plants pale greenish to brownish greenish, prostrate, soft, forming loose patches, 1.2–2.5 mm wide and 20–30 mm long. Stem greenish, branching not seen. Rhizoids abundant, originating from the stem, mostly in the areas adjacent to ventral leaf base, obliquely to erect spreading, separated or in unclear fascicles, rigid to merely soft, purple to (rarer) purplish and brownish. Leaves contiguous, subhorizontally inserted and oriented, obliquely to erect spreading, nearly plane to slightly convex, obliquely lingulate, entire at margin, apex rounded to truncate and shallowly emarginate, 0.8–1.4 × 0.6–1.2 mm. Midleaf cells 40–60 × 30–40 µm, thin-walled, trigones very small, concave cuticle densely papillose-verrucose; cells along leaf margin 28–43 µm, thin-walled, with thin or slightly thickened external wall, trigones small, concave, cuticle distinctly verrucose. Dioicous. Androecia intercalary, with 2–3 pairs of bracts (Figure 43D–J and Figure 44A,B).

Comment: The species was synonymized with the very broadly treated *P. truncata* (*Jungermannia truncata* in that paper) by Váňa and Inoue [14]; however, it is distinctive due to its thin-walled cells even along the leaf margin, and densely and coarsely papillose-verrucose and commonly rigid and purple rhizoids.

*Nardia paroica* Schiffn., Lotos 58: 320, 1910.

Type: UK, England, Cumberland, Dalegarth, Boot, Eskdale, auf lehminger Erde, ca 260 m; April 1906, W.H. Pearson (V. Schiffner’s Hepaticae Europaeae Exsiccatae, no. 374) (Isotypus PC0102753!).

Accepted name: *Solenostoma paroicum* (Schiffn.) R.M.Schust., Amer. Midl. Naturalist 49 (2): 402, 1953

**Figure 45:**
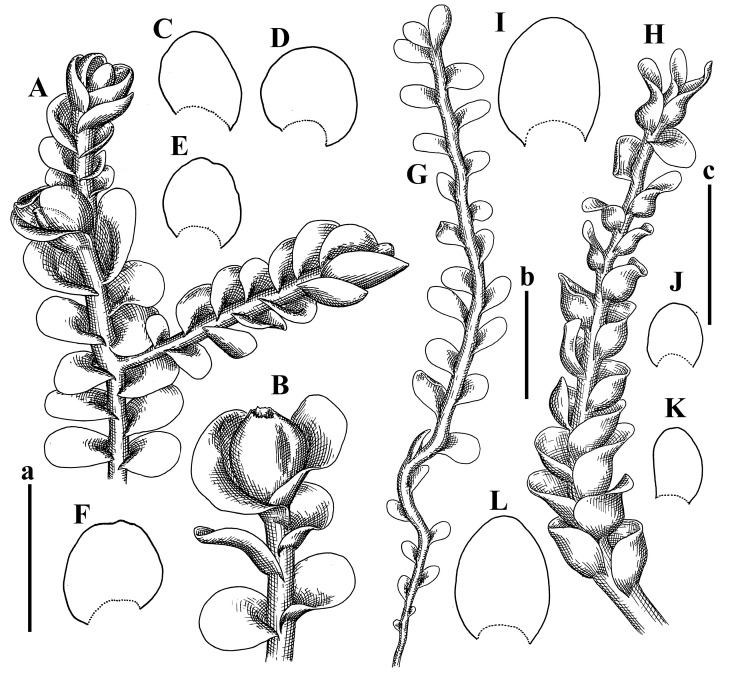
*Nardia paroica* Schiffn. Isotypus, PC–0102753: (**A**,**B**) perianthous plants; (**C**–**F**) leaves. *Nardia subtilissima* Schiffn. Isotypus, HIRO, s.n.: (**G**) plant habit; (**H**) male branch; (**I**–**L**) leaves. Scales: a—2 mm for (**A**–**F**); b—1 mm for (**G**–**I**,**L**); c—1 mm for (**J**,**K**).

Description: Plants yellowish brownish, with evident purplish tint (plants become purplish in ventral or distal sides of leaves and stem, especially on its ventral side), or purplish pigmentation is nearly absent and plants pale greenish–yellowish, merely soft, slightly hyaline, forming loose patches, closely attached by rhizoids to the substratum, 1500–1750 µm wide and 7–15 mm long. Rhizoids dense to common, erect to obliquely spreading, mostly united in unclear fascicles, or decurrent down along the stem, but not forming distinct fascicle, colorless to brownish, but in areas near origin (near stem or perigynium) purplish to nearly purple. Leaves contiguous, obliquely inserted, dorsally distinctly but shortly (max. 1/4 of stem width) decurrent, obliquely spreading and obliquely oriented, concave (sometimes strongly) to concave–canaliculate, when flattened in the slide obliquely reniform to obliquely ovate, commonly distinctly retuse at the apex, 1000–1300 × 1250–1750 µm. Midleaf cells 37.5–62.5 × 37.5–50.0 µm, thin-walled, trigones moderate in size, convex, cuticle smooth; cells along margin 32.5–40.0 µm, thin-walled, external wall slightly to evidently thickened, trigones moderate in size, bulging. Paroicous. Perianth nearly spherical, with four main plicae, composed of subisodiametric to shortly oblong cells with moderate-in-size, convex trigones (similar in all features to that in the cells in the midleaf), mouth crenulate; perigynium absent to vestigial, rarer with one pair of bracts and ca 1/4 of perianth length; female bracts similar to large leaves, commonly undulate at margin and retuse at apex. Androecia with 1–2 pairs of bracts, monandrous (at least, we observed this), bracts ventricose in lower half and recurved above. Spores 24–27 µm in diameter, papillose, brown. Elaters bispiral, ca 250 × 10 µm, with long, ca. 50 µm, homogenous ends (Figure 44C–H and Figure 45A–F).

Comment: This species is similar in many ways to *Solenostoma hyalinum* in prostrate growth, colorless to purplish rhizoids, hyaline habit, wide and retuse to emarginate leaf apices, although different in paroicous inflorescence.

*Nardia subtilissima* Schiffn., Ann. K. K. Naturhist. Hofmus. 23: 136, 1909.

Type: Asia Minor, districtus Trapezunti, prope viculum Bakajak (ad meridiem ab opidu Ordu), ad rupes umbratas in faucibus silvaticis Kabak Deressi, substrato andesitico, ca 650 m a.s.l.; 3 August 1907 Handel-Mazetti (Isotypus HIRO, s.n!).

Accepted name: *Solenostoma subtilissimum* (Schiffn.) R.M.Schust., Hepat. Anthocerotae N. Amer. 2: 1027, 1969.

Description: Plants prostrate, more or less closely attached by rhizoids to the substratum, encrusted by soil particles, 0.4–0.9 mm wide and 3–8 mm long, brownish green to dark– and dirty–green, forming loose mats, soft. Stem sparsely laterally branched, (30)40–80 µm in diameter, brownish, marginal cells in the cross section with only slightly thinner walls than inward. Rhizoids sparse to numerous, colorless, erect to obliquely spreading and forming mats under the stem. Leaves obliquely to very obliquely and almost horizontally inserted and oriented, obliquely spreading, mostly flattened, rarely slightly concave–canaliculate, in larger shoots loosely sheathing the stem in the base, nearly ovate, 240–440 × 200–370 µm, distant. Midleaf cells 20–26 × 18–24 µm, thin-walled, with moderate-in-size, triangle to slightly convex trigones; cuticle smooth to indistinctly papillose; along leaf margin 14–20 µm, thin-walled, with small to medium in size, concave trigones, external wall not thickened, cuticle loosely papillose. Dioicous. Perianth conical, ca. 300 × 200 µm, loosely plicate, exerted from bracts for 2/3 of its length, composed of elongated cells; perigynium 2 times longer than perianth, with 2 pairs of bracts, bare (not covered by bracts); female bracts erect spreading, nearly transversely inserted, similar to large leaves or slightly wider. Androecia intercalary, spicate, with 5–7 pairs of bracts, bracts strongly inflated and recurved along margin (Figure 44I and Figure 45G–L).

Comment: This species was regarded by Váňa [23] as closely related to *Solenostoma hokkaidense*. Indeed, the two taxa are quite similar both in general habit and habitat (andesite). However, aside from distribution, *S. subtilissimum* seems to be different from *S. hokkaidense* in its deeper color, monomorphous stem structure (probably due to the smaller size of the plants), slightly papillose leaf cuticle, and more elongated leaves. Discussion about the relationships of the two species is also placed under *S. hokkaidense*.

*Plectocolea biloba* S.Hatt. ex Amakawa, J. Jap. Bot. 32 (7): 216, 1957.

Type: Japan, “Hokkaido, Hidaka, near Samani along Porosdan-shibetsu River, 200 m alt., in greywacke crevice, Shimizu” [27], 11.VIII.1954 (Holotypus NICH-54772!).

Accepted name: *Solenostoma bilobum* (S.Hatt. ex Amakawa) Potemkin et Nyushko, Liverworts and Hornworts of Russia 1: 286, 2009.

**Figure 46:**
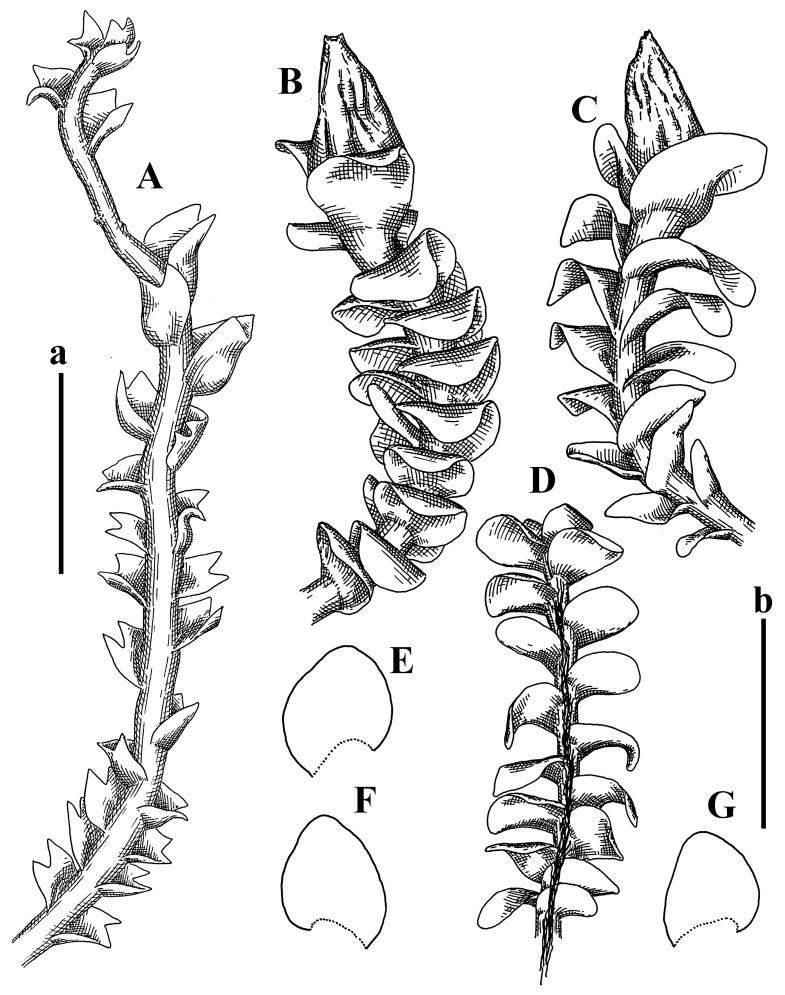
*Plectocolea biloba* S.Hatt. ex Amakawa Holotypus, NICH–54772: (**A**) male plant. *Plectocolea erecta* Amakawa Holotypus, NICH–64108: (**B**,**C**) perianthous plants; (**D**) plant habit ventral view; (**E**–**G**) leaves. Scales: a—1 mm for (**A**); b—2 mm for (**B**–**G**).

Comment: The plants are very similar to those described in the Russian Far East in Bakalin [10] and in the original description [27]. This species is very distinctive due to its bilobed leaves (Figure 46A).

*Plectocolea erecta* Amakawa, J. Jap. Bot. 32 (10): 307, 1957.

Type: Japan. “Yakushima Isl., between Kosugidani-Hananoego” [28] (original label is in Japanese) 1100 m alt.; 26 March 1956, T. Amakawa 2158 (Holotypus NICH-64108!).

Accepted name: *Plectocolea erecta* Amakawa, J. Jap. Bot. 32 (10): 307, 1957. (=*Solenostoma erectum* (Amakawa) C.Gao, Fl. Hepat. Chin. Boreali-Orient.: 66, 1981)

**Figure 47:**
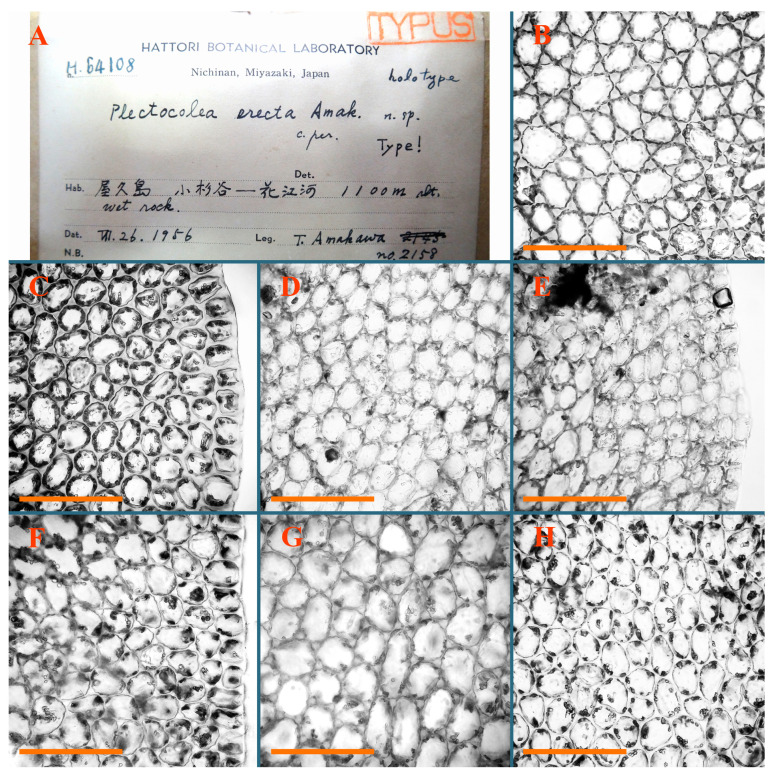
*Plectocolea erecta* Amakawa Holotypus, NICH–64108: (**A**) holotype label; (**B**) midleaf cells; (**C**) marginal cells. *Plectocolea hattoriana* Amakawa Holotypus, NICH–73004: (**D**) midleaf cells, (**E**) leaf margin cells. *Plectocolea horikawana* Amakawa Holotypus, NICH–64109: (**F**) leaf margin cells; (**G**) midleaf cells. *Plectocolea marginata* S. Hatt. Holotypus, NICH–12720: (**H**) midleaf cells. Scales: 100 µm for (**B**–**H**).

Comment: A perfect description is in the work of Amakawa [8]; only a few general comments are worth mentioning: (1) plants rusty brownish to yellowish; (2) rhizoids in distinct deep purple fascicle; (3) leaves subtransversely inserted and subtransversely oriented, loosely sheathing the stem near base and erect spreading and recurved in upper half; (4) leaves ovate-triangular (in a manner similar to *J. eucordifolia*); (5) trigones moderate in size to (mostly) large, convex to coarsely nodulose. This species is a distinct species due to its erect to ascending growth; its subtransversely inserted, but in a sterile state commonly obliquely oriented, purple rhizoids in fascicle; and its thin-walled leaf cells originating on the ventral side of the stem, (also including the leaf margin). Its trigones are commonly prominent, convex, and moderate to large in size; the perigynium is 1/4–1/3 of the perianth length (Figure 46B–G and Figure 47A–C).

*Plectocolea hattoriana* Amakawa, J. Jap. Bot. 33 (11): 341, 1958.

Type: Japan, Nagano Prefecture, Mt. Yatsu, 2550 m alt., on rock along stream near ridge-top; 12 August 1952 D. Shimizu (Holotypus NICH-73004!).

Accepted name: *Plectocolea hattoriana* Amakawa (=*Solenostoma hattorianum* (Amakawa) Potemkin et Nyushko, Liverworts and Hornworts of Russia 1: 287, 2009)

**Figure 48:**
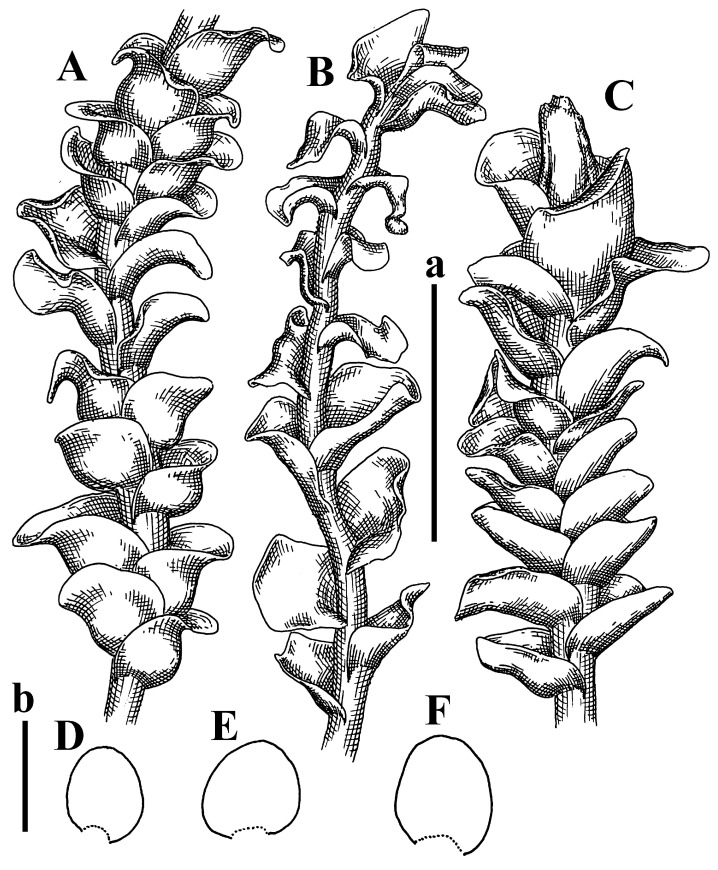
*Plectocolea hattoriana* Amakawa Holotypus, NICH–73004: (**A**) male branch fragment; (**B**) plant habit; (**C**) perianthous plant; (**D**–**F**) leaves. Scales: a—2 mm for (**A**–**C**); b—1 mm for (**D**–**F**).

Description: Plants ascending, brownish, in loose patches, 0.9–1.6 mm wide and 7–12 mm long. Stem 150–200 µm in diameter, branching as geotropic stolons that at least sometimes become normal branches; plants, where observable, originate from rhizomatous base, with stem with distinct hyalodermis in the cross-section. Rhizoids sparse to common, colorless to brownish (and as an exception, purplish in some areas), not rigid, obliquely spreading, and commonly united into unclear fascicles. Leaves contiguous to (rarer) distant, slightly concave to concave–canaliculate, sometimes undulate at margin also sometimes loosely sheathing the stem in lower third and spreading above, leaves obliquely to erect spreading, sometimes (perianthous branches) enclosed with one another, sub- to transversely inserted in fertile branches and subtransversely to obliquely inserted in sterile branches (similarly to above orientation, which varies from oblique to transverse), nearly ovate, 700–1000 × 800–1000 µm. Midleaf cells subisodiametric to shortly oblong, 25–37 × 20–27 µm, thin-walled, trigones moderate in size, slightly convex, cuticle smooth; cells along leaf margin 15–23 µm, thin-walled, with more or less thin external wall, trigones moderate in size, mostly concave, cuticle smooth. Dioicous. Androecia intercalary, although branches sometimes become depauperate and die after some pairs of sterile leaves following androecia, with 6–8 and more pairs of bracts; bracts inflated in lower half and erect spreading (margin recurved) above. Perianth conical, ca. 700 × 700 µm, composed of elongated cells; perigynium ca. 1–1.8 perianth length, with 2 pairs of bracts; bracts sheathing perianth for the whole extent (lower pair with obliquely spreading upper half) (Figure 47D,E and Figure 48).

Comment: The holotype of the taxon represents an ill-developed phase of the species that commonly has rigid and deep purple rhizoids. The striking features of the taxon are also a distinct hyaloderm (although not so much as, e.g., in *Solenostoma obscurum*) and subtransversely inserted (sometimes enclosed with one another) leaves. The species is somewhat related to *Plectocolea schusteriana* (J.D. Godfrey & G. Godfrey) Bakalin also in the feature of its oil bodies, from which differs in geographic distribution, a smaller size of plants, and a not-so-high perigynium. All listed are the subjects of great variation, and a DNA analysis comparison should be carried out to elucidate the relationships of these two taxa.

*Plectocolea horikawana* Amakawa, J. Jap. Bot. 32 (7): 219, 1957.

Type: Japan, “Nagasaki, Mt. Taradake, 400 m, on rock” [27]; 24 March 1957 T. Amakawa 2259 (Holotypus NICH-64109!).

Accepted name: *Plectocolea horikawana* Amakawa (=*Solenostoma horikawanum* (Amakawa) Váňa, Hentschel et Heinrichs, Cryptog. Bryol. 31 (2): 137, 2010)

**Figure 49:**
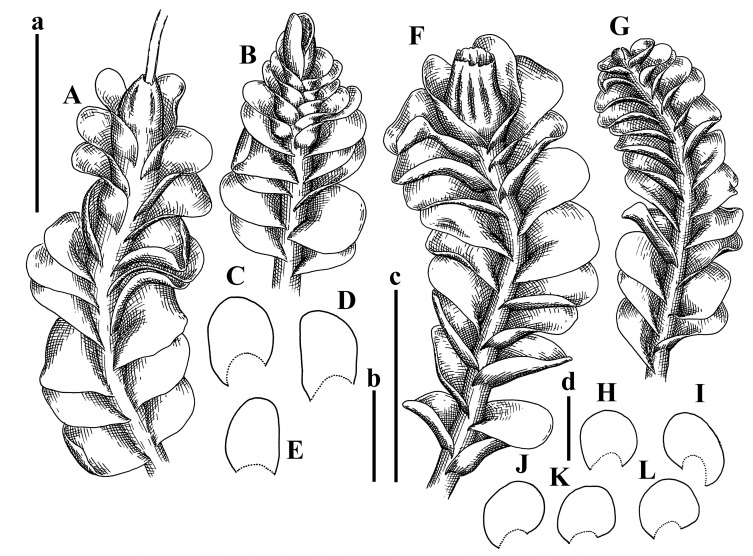
*Plectocolea horikawana* Amakawa Holotypus, NICH–64109: (**A**) perianthous plant; (**B**) male plant; (**C**–**E**) leaves. *Plectocolea marginata* S. Hatt. Holotypus, NICH–12720: (**F**) perianthous plant; (**G**) plant habit; (**H**–**L**) leaves. Scales: a—3 mm for (**A**,**B**); b—2 mm for (**C**–**E**); c—2 mm for (**F**,**G**); d—1 mm for (**H**–**L**).

Description: Plants prostrate to loosely ascending, rather soft, brownish to yellowish brownish (color probably not natural due to age), forming loose to dense patches, 2.0–3.3 mm wide, 5–15 mm long. Stem 250–300 µm in diameter, brownish, sparsely laterally branched. Rhizoids very dense, brownish to purplish (we did not observe a distinct purple color, although it is indicated by Amakawa [8]), erect to obliquely spreading and forming dense mat under stem. Leaves contiguous to subimbricate, enclosed with oneanother or sometimes overlapping, concave–canaliculate to canaliculate–concave, sometimes with convex antical side (as in the image from Amakawa [27], but that variant is not commonly present), obliquely inserted, decurrent up to 1.0 stem width dorsally and subtransversely to arcuately inserted ventrally; where not or barely decurrent, 1300–1800 × 1000–1600 µm, obliquely ovate to obliquely lingulate, obliquely spreading and obliquely to subhorizontally oriented. Midleaf cells 32.5–60 × 32.5–55 µm, subisodiametric to oblong, thin-walled, trigones moderate in size, convex, cuticle smooth; cells along margin 17–30 µm, nearly thin-walled, external wall slightly thickened, trigones moderate in size to (rarer) large, convex, cuticle smooth. Dioicous. Androecia intercalary (but within 4–6 pairs of sterile leaves above the branch, they depauperate and die), with 5–7 pairs of bracts, bracts strongly inflated in lower 1/3–1/2 and concave–canaliculate to canaliculate above, with commonly recurved margin. Perianth ca. 1300 × 900 µm, conical, pluriplicate, hidden within bracts or exerted for 1/3 of its length, composed of elongated cells; perigynium 1/2–2/3 of perianth length, with 1–2 pairs of bracts; female bracts similar to large leaves, sometimes slightly undulate at margin, sheathing perianth, canaliculate, not– or deflexed in upper 1/4–1/3 (Figure 47F,G and Figure 49A–E).

Comment: This taxon is very similar to *Plectocolea infusca* Mitt., from which it differs in its biconcentric oil bodies and more plane or (rarely) somewhat convex leaves. This species seems to occur in lowland evergreen forests in southern Japan, and is hardly to be expected in northern latitudes.

*Plectocolea marginata* S. Hatt., J. Hattori Bot. Lab. 3: 40, 1948 [1950].

Type: Japan. Prov. Higo, Kuma-gun, Mt. Ichifusa, ca 1250 m alt., on granitic rocks; 14 September 1947, K. Mayebara 842 (Holotypus NICH12720!)

Accepted name: *Plectocolea marginata* S.Hatt. (=*Solenostoma marginatum* (S.Hatt.) R.M.Schust., Hepat. Anthocerotae N. Amer. 2: 983, 1969)

**Figure 50:**
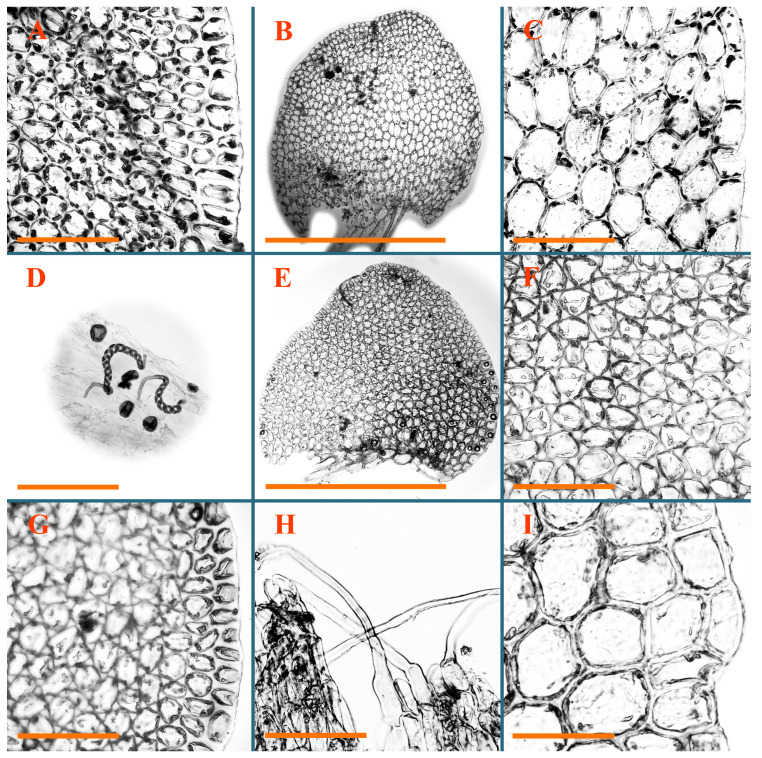
*Plectocolea marginata* S. Hatt. Holotypus, NICH–12720: (**A**) leaf margin cells. *Plectocolea otiana* S.Hatt. Holotypus, NICH–12044: (**B**) leaf; (**C**) leaf margin cells; (**D**) spores and elaters. *Plectocolea rigidula* S.Hatt. Holotypus, NICH–12707: (**E**) leaf; (**F**) midleaf cells; (**G**) leaf margin cells. *Plectocolea setulosa* Herzog, JE04000810: (**H**) perianth mouth; (**I**) leaf margin. Scales: 100 µm for (**A**,**C**,**D**,**F**–**H**); 1 mm for (**B**,**E**); 50 µm for (**I**).

Description: Plants more or less soft, pallid, yellowish brownish, in loose to dense patches, ascending to erect, 1.0–1.8 mm wide and 7–15 mm long. Stem 120–170 µm in diameter, not branched, brownish. Rhizoids numerous, brownish to with slight purplish tint, decurrent down and forming distinct fascicle the similar diameter than the stem or even larger. Leaves distant to contiguous (then enclosed with one another), concave–canaliculate to canaliculate, ‘canal line’ recurved down, subtransversely inserted, decurrent for 1/2–2/2 of stem width in dorsal side and transversely to arcuately inserted ventrally; where not or barely decurrent, when flattened in the slide, widely obliquely ovate to suborbicular and obliquely transversely elliptic, 600–850 × 900–1000 µm, transversely oriented, with easily visible leaf rim on larger cells. Midleaf cells subisodiametric to oblong, 25–42 × 22–30 µm, thin-walled with moderate-in-size to large convex trigones, cuticle smooth; cells along leaf margin larger (sometimes only slightly larger) than the next row inward and noticeably unequally thickened (sometimes only external wall strongly thickened), 20–30(50) µm, trigones large, convex, sometimes confluent on tangential wall, cuticle smooth. Dioicous. Perianth long conical, pluriplicate, ca. 1500–2000 × 600 µm, exerted for 2/3–4/5 of its length; perigynium less than 1/4 of perianth length, with 1 pair of bracts; bracts similar to large leaves, but comparatively wider, undulate at margin, sometimes shallowly emarginate at apex, sheathing perianth in its lower half (Figure 47H, Figure 49F–L and Figure 50A).

Comment: The taxon is superficially similar to *Solenostoma suborbiculatum,* but differs in its well-developed perigynium and other perianth characteristics. The subsequent interpretation by Amakawa [8] may be wrong; he wrote that the rhizoids were purple, although in this type they are colorless. That said, in his type description, Hattori [29]: 40 wrote “pallidis vel ± purpureis”.

*Plectocolea otiana* S.Hatt., J. Jap. Bot. 28 (6): 183, 1953.

Type: Japan, Prov. Iyo, Honaru Hill, Sumino-cho, Nii-gun; 24 October 1945 K. Oti 1123 (Holotypus NICH12044!).

Accepted name: *Plectocolea otiana* S.Hatt. (=*Solenostoma otianum* (S.Hatt.) R.M.Schust., Hepat. Anthocerotae N. Amer. 2: 984, 1969).

**Figure 51:**
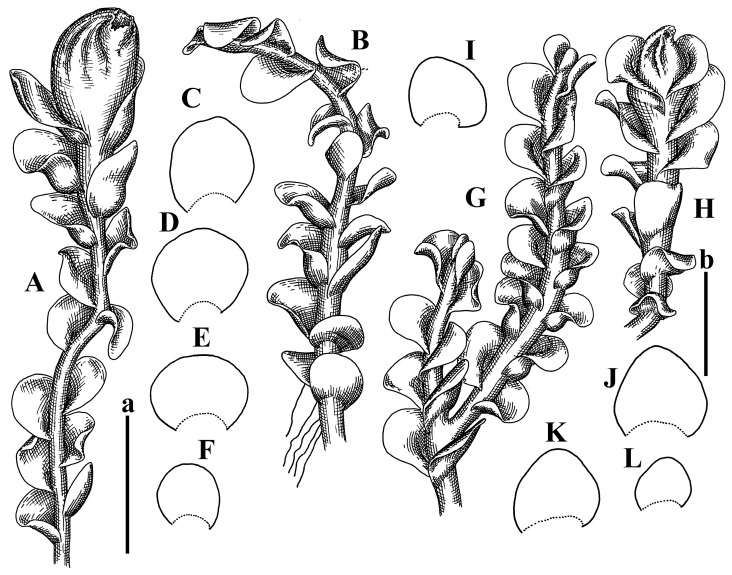
*Plectocolea otiana* S.Hatt. Holotypus, NICH–12044: (**A**) perianthous plant; (**B**) plant habit; (**C**–**F**) leaves. *Plectocolea rigidula* S.Hatt. Holotypus, NICH–12707: (**G**) plant habit; (**H**) perianthous plant; (**I**–**L**) leaves. Scales: a—2 mm for (**A**–**F**); b—2 mm for (**G**–**L**).

Description: Plants ascending to erect, in loose patches, 0.7–2.5 mm wide, yellowish brownish, more or less soft. Stem 70–200 µm in diameter, not branched (even as subfloral innovations). Rhizoids rather numerous, obliquely to erect spreading, colorless to brownish and purplish in sterile and male branches and purple and more or less rigid in fertile paroicous branches, separated or united into unclear fascicles. Leaves distant, obliquely inserted, sometimes slightly undulate, or up to 1/3 of stem width or non-decurrent dorsally and transversely inserted and not or barely decurrent ventrally, obliquely spreading and obliquely oriented, mostly obliquely ovate, well-developed branch leaves ca. 900–1100 × 900–1200 µm, in weak shoots leaves starting from 320 × 250 µm; large leaves sometimes emarginate at the apex. Midleaf cells shortly oblong, penta– to hexagonal, 45–70 × 37–50 µm, thin-walled, with small, concave trigones, cuticle smooth; cells along leaf margin 30–45 µm, nearly thin-walled, with slightly thickened external wall, trigones moderate in size, concave, cuticle smooth. Mostly monoicous, but also with male branches. Monoicous phase: Perianth pluriplicate, commonly with 3 main plicae, long conical, mostly not turbinate at the mouth, 1200–1500 × 600 µm, composed of elongated cells; perigynium ca. 1/5 of perianth length or less; bracts sheathing perianth near base and obliquely to erect spreading in upper 1/3–1/2; androecia in 2–3 pairs below female bracts, inflated in lower half and erect spreading above, both male and female bracts commonly undulate and shallowly retuse at apex. Androecial branches: with 2–3 pairs of bracts, no sequence of generation was observed, after androecia branch produce 5–7 or even more pairs of sterile leaves, then becomes gradually depauperate and die out. Spores finely papillose, brown, ca. 15 µm in diameter. Elaters bispiral, ca. 150 × 7–8 µm (spores are described for the first time from NICH–198520)] (Figure 50B–D and Figure 51A–F).

Comment: The species is distinct due to its heteroicous inflorescence, where paroicous plants dominate, although male branches also sporadically occur, and other features include a low perigynium and long-exerted perianth.

*Plectocolea rigidula* S.Hatt., J. Jap. Bot. 27 (2): 53, 1952.

Type: Japan. Prov. Higo, Mizukami, ca 750 m alt., rock; 2 November 1947 K. Mayebara 988 (Holotypus NICH12707!).

Accepted name: *Plectocolea rigidula* S.Hatt. (=*Solenostoma rigidulum* (S.Hatt.) R.M.Schust., Hepat. Anthocerotae N. Amer. 2: 983, 1969)

Comment: The species was described in enough detail by Amakawa [8] and Bakalin [10]. The morphological variability of the population is wider than the variability of plants in the type, and the following short description should be noted:

Stem sparsely laterally branched (branches in the sinus near ventral leaf base). Rhizoids rigid, purple–violet to purplish. Midleaf cells 20–38 × 20–30 µm, nearly thin-walled, with large, convex trigones (rarely with visible median lamina), cuticle smooth; cells along leaf margin 12.5–30.0 µm, strongly unequally thickened, trigones moderate in size to large, convex. Androecia intercalary, with 2–3 pairs of bracts; bracts inflated in lower half and canaliculate and recurved down in upper 1/3–1/2 (Figure 50E–G and Figure 51G–L).

The species is very morphologically similar to *Plectocolea crenuliformis* (Austin) Mitt., from which it differs in its distribution and more translucent appearance. Its taxonomic relationships are unclear, as was stressed by Amakawa [8].

*Plectocolea setulosa* Herzog, J. Hattori Bot. Lab. 14: 33, 1955.

Type. Taiwan, “Kwashyoto: Erdhang in feucht-schattiger Bachrinne, leg. G.H. Schwabe, 30.5.1947, no 63” [30]. (Holotypus JE04000809!; Epitypus (designated here) Schwabe 62, JE04000810!).

Accepted name: *Plectocolea setulosa* Herzog (=*Solenostoma truncatum* var. *setulosum* (Herzog) Váňa et D.G.Long, Nova Hedwigia 89 (3/4): 510, 2009)

Comments: 1) The main identification feature of this taxon is the setulose perianth mouth; other distinctions are not so clear, and include very soft-textured plants with thin-walled leaf cells (slightly papillose-verrucose along leaf margin). Cells are also thin-walled along the margin, and no border of thick-walled cells is developed along the margin. Rhizoids are colorless to purple, originating mostly near ventral leaf base, decurrent down the stem, but not forming even loose fascicles. In other traits and superficially, it is similar to *P. truncata* s. str.

**Figure 52:**
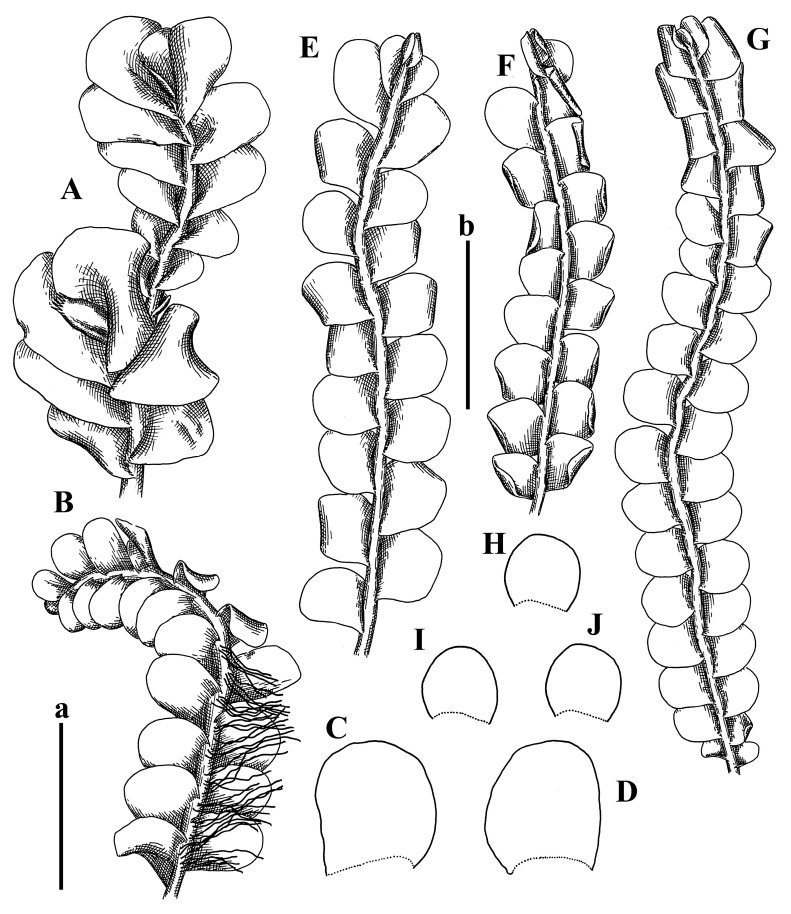
*Plectocolea setulosa* Herzog, Epitypus, JE04000810: (**A**) perianthous plant; (**B**) plant habit ventral view; (**C**,**D**) leaves. *Plectocolea sordida* S. Hatt. Holotypus NICH–225232: (**E**) plant habit; (**H**–**J**) leaves; Isotypus, JE04000808: (**F**) plant habit ventral view; (**G**) plant habit. Scales: a—2 mm for (**A**–**D**); b—3 mm for (**E**–**J**).

**Figure 53:**
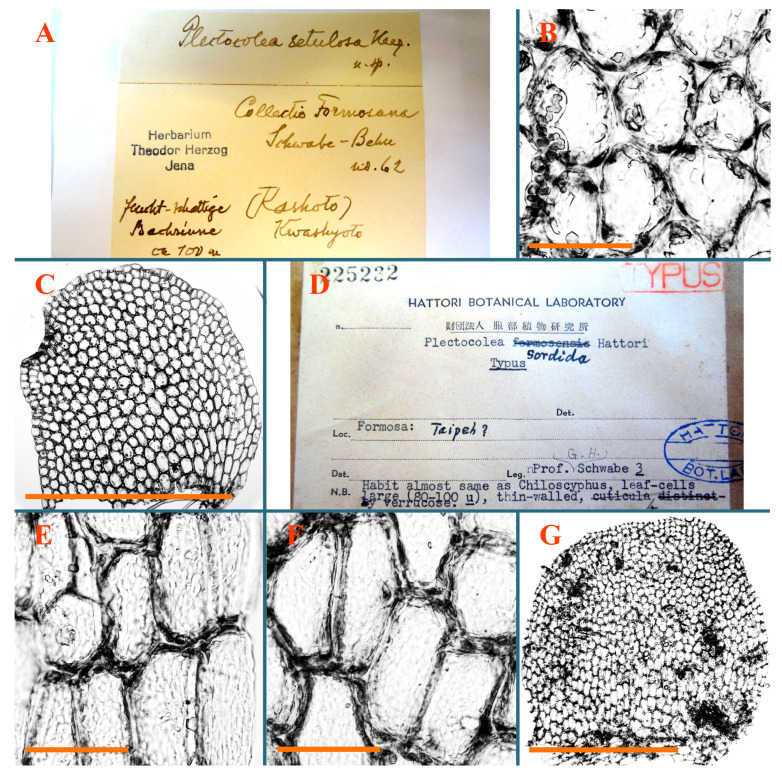
*Plectocolea setulosa* Herzog, Epitypus, JE04000810: (**A**) specimen label Schwabe62; (**B**) midleaf cells, (**C**) leaf. *Plectocolea sordida* Holotypus NICH–225232: (**D**) holotype label; (**E**) leaf margin cells showing papillae; (**F**) midleaf cells; (**G**) leaf. Scales: 50 µm for (**B**,**E**,**F**); 1 mm for (**C**,**G**).

2) The specimen (Schwabe 63), although marked as a holotype, can hardly be used for the purpose of the correct identification. Another specimen (Schwabe 62, JE04000810), virtually from the same place, was probably used to compile the original type description, because in the original description, Herzog mentions a ‘setulose’ perianth mouth. Meanwhile, the material in ‘Schwabe 62′ is perianthous, contrary to perianthless ‘Schwabe 63′. Moreover, the former specimen (‘Schwabe 62′) contains well-developed plants, characterized by rigid purplish rhizoids (not totally colorless to brownish as in Schwabe 63, although not purplish (‘purpurascentibus’, as in the original source)), more robust plants, leaf cells with well-developed, moderate-in-size convex trigones, and thickened (mostly unequally, although sometimes subequally) cell walls along the leaf margin. The cuticle is slightly papillose-verrucose along the leaf margin and virtually smooth in the midleaf (not as smooth as in the holotype). Following Article 9.9 of the International Code of Nomenclature for Algae, Fungi, and Plants, “An epitype is a specimen or illustration selected to serve as an interpretative type when the holotype, lectotype, or previously designated neotype, or all original material associated with a validly published name, is demonstrably ambiguous and cannot be critically identified for purposes of the precise application of the name to a taxon”. Therefore, we suggest proposing an epitype for the taxon using the specimen Schwabe 62, JE04000810 (Figure 50H,I, Figure 52A–D and Figure 53A–C).

*Plectocolea sordida* S. Hatt. Journal of the Hattori Botanical Laboratory 14: 34. f. 1: e–n. 1955.

Type: Taiwan, ‘Formosa’, Taipeh(?); G.H. Schwabe 3 (Holotypus NICH225232!; there are three isotypes in JE (JE04000806!, JE04000807!, JE04000808!); moreover, the last one is marked as a holotype, although the holotype should be in NICH, taking into account the description by Hattori. The original publication [30] does not indicate the herbarium. In any case, all specimens contain identical plants.

Accepted name: *Plectocolea sordida* S. Hatt.

Description: Plants similar to *Chiloscyphus polyanthos* in general appearance, prostrate, 2–3 mm wide, 10–20 mm long, brownish, forming loose patches, dorsiventrally flattened. Stem 150–200 µm in diameter, not branched, brownish. Rhizoids sparse, colorless to brownish, originating from ventral side of stem mostly near ventral leaf base, obliquely to erect spreading or forming mat in ventral side of stem. Leaves nearly horizontally inserted, orbicular–lingulate to widely ovate-lingulate, rarely with retuse apex, flattened to indistinctly convex (giving aspect of *Chiloscyphus*), 900–1200 × 1000–1300 µm, distant to contiguous. Midleaf cells 37–75 × 32–55 µm, thin-walled, trigones vestigial to very small, cuticle distinctly papillose throughout; cells along leaf margin 37–67 µm, thin-walled, trigones very small and concave, cuticle distinctly papillose (Figure 52E–J and Figure 53D–G).

Comment: The species was synonymized with P. *truncata* by Váňa and Inoue [14]. However, it is difficult to agree with this decision. *Plectocolea sordida* was described based on sterile material, from a probably submerged station (“aquatica?” in its type description [30]). Plants annotated by Hattori in the holotype label as “Habit almost same as *Chiloscyphus*, leaf cells large (80–100 µm), thin-walled, cuticle more or less verrucose”. The cell cuticle is indeed noticeably papillose throughout the leaf; this feature is hardly expected in the aquatic phase of *P. truncata* (which commonly have no papillae, even in mesic habitats). Other features include slightly convex leaves, an absence of thick-walled cells along the leaf margin, and large leaf cells.

*Solenostoma clavellatum* Mitt. ex Steph., Bull. Herb. Boissier (sér. 2) 1 (5): 491 (53), 1901.

Type: China, Yunnan, in monte Tsang-Chan, 4000 ft., Delavay 4047(Lectotypus [21] BM s.n.!; Isolectotypus: G00281662!)

Accepted name: *Solenostoma clavellatum* Mitt. ex Steph.

**Figure 54:**
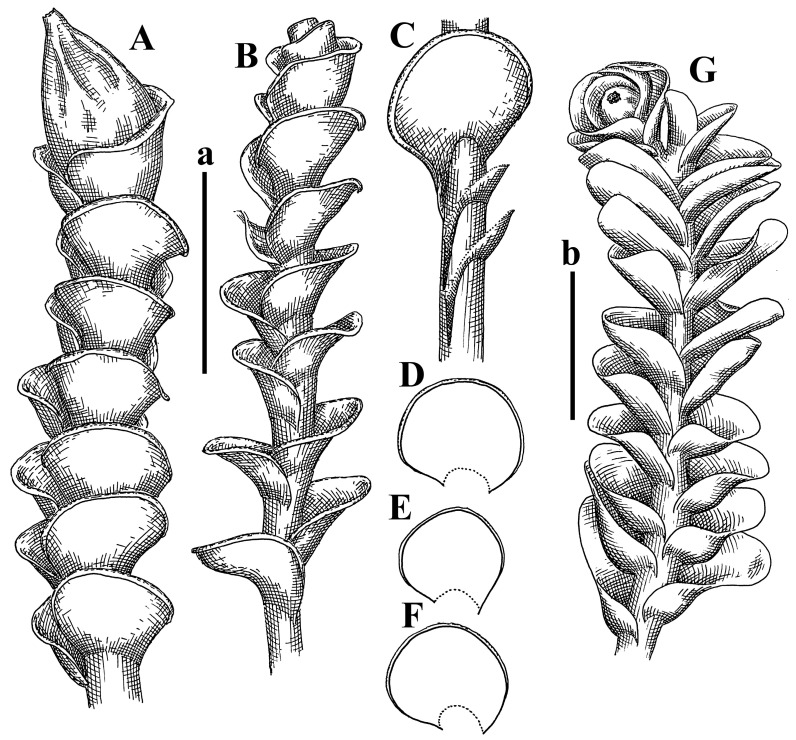
*Solenostoma clavellatum* Mitt. ex Steph. Lectotypus, BM s.n.: (**A**) perianthous plant; (**B**) plant habit; (**C**) leaf insertion feature; (**D**–**F**) leaves abaxial view. *Solenostoma hiugaense* Amakawa Isotypus, SAP, s.n.: (**G**) plant habit. Scales: a—2 mm for (**A**–**F**); b—2 mm for (**G**).

**Figure 55:**
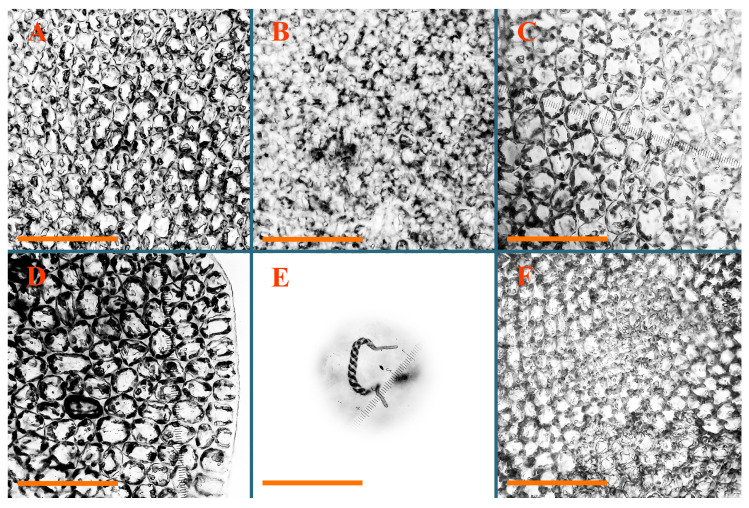
*Solenostoma clavellatum* Mitt. ex Steph. Lectotypus, BM s.n.: (**A**) midleaf cells; (**B**) midleaf cell cuticle showing papillae. *Solenostoma hiugaense* Amakawa Isotypus, SAP, s.n.: (**C**) midleaf cells; (**D**) leaf margin cells; (**E**) elater. *Solenostoma rishiriense* Amakawa Holotypus, NICH–53527: (**F**) midleaf cells. Scales: 100 µm for (**A**–**F**).

Description: Plants yellowish brownish in lower part of shoot and purplish to purple in upper part, erect, rigid, forming loose patches, 800–1700 µm wide (perianthous 1500–1700 µm) and 10–15 mm long, sparsely laterally branched. Rhizoids numerous, colorless to grayish, forming distinct fascicle decurrent down along dorsal (at least in the most cases) side of the stem, originated in outer side of leaf lamina and ventral side of lower half of the perianth. Leaves subtransversely to almost obliquely inserted, decurrent in dorsal side for 1–2 stem widths and in ventral side for 1/2–2/2 of stem width, contiguous to subimbricate, obliquely spreading, subtransversely oriented, nearly transversely elliptic, 550–1100 × 700–1600 µm, strongly recurved to revolute along margin (with the exception of smaller leaves). Midleaf cells isodiametric to shortly oblong, 20–30 × 20–28 µm, thin-walled, trigones large, convex, cuticle strongly papillose; cells along leaf margin commonly tangentially elongated, 8–18 µm, unequally thickened, trigones large, slightly convex, cuticle papillose–verrucose. Dioicous. Perianth 2000 × 900 µm, widely fusiform, 2–4–stratose in lower half, gradually narrowing to loosely beaked mouth, exerted for 2/3 of the length; perigynium, if present, ca. 1/5 of perianth length; bracts similar to leaves, sheathing the perianth (Figure 54A–F and Figure 55A,B).

Comments. (1) As was pointed out by Váňa [21], the species is closely related to *Solenostoma atrorevolutum* (Grolle ex Amakawa) Váňa & D.G. Long, from which it differs in its smaller plants, brown coloration, smaller leaf cells with smooth cuticle, and non-rhizogenous leaves.

(2) The somewhat confusing interpretation of the present taxon was described in detail by Váňa [21], who selected the lectotype for the species. After recent searches in herbarium G, we were able to find the specimen unavailable at the time Váňa visited G (it was not in the type collection, but in the general collection); it had the same field number and contained copious material, much more than in BM, and with a more easily recognizable lectotype than the latter. However, to avoid additional confusion, we follow Váňa’s lectotypification and regard G00281662 as an isolectotype.

*Solenostoma hiugaense* Amakawa, J. Jap. Bot. 31 (2): 47, 1956.

Type: Japan. Miyazaki Prefecture, Mt. Ogue, ca 900 m a.s.l., on moist granitic rocks; 1 April 1953, T. Amakawa s.n. (Isotypus SAP, s.n. [published in Hepaticae Japonicae Exsiccatae, ser. 6 (1954), No. 267]; the holotypus should be in NICH [31], but was not located in that herbarium).

Accepted name: *Solenostoma hiugaense* Amakawa, J. Jap. Bot. 31 (2): 47, 1956.

**Figure 56:**
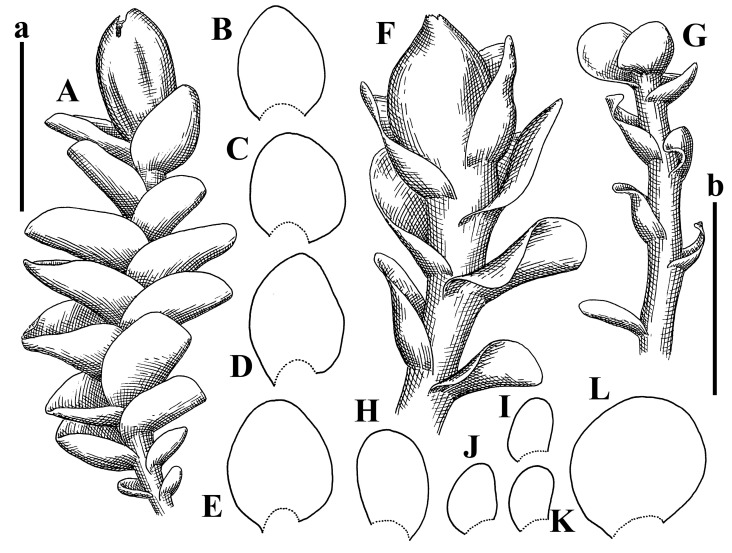
*Solenostoma hiugaense* Amakawa Isotypus, SAP, s.n.: (**A**) perianthous plant; (**B**–**E**) leaves. *Solenostoma rishiriense* Amakawa Holotypus, NICH–53527: (**F**) perianthous plant; (**G**) plant habit; (**H**–**L**) leaves. Scales: a—2 mm for (**A**–**E**); b—1 mm for (**F**–**L**).

Description: Plants rather erect, dirty green to greenish yellowish, brownish yellowish and brownish purplish, commonly tinged with red or purple in upper part of shoot, more or less rigid, 1.5–2.8 mm wide and 8–15 mm long, forming more or less dense patches. Stem 200–350 µm in diameter, rarely laterally branched (also as subfloral innovations), slightly flexuous. Rhizoids sparse to dense, colorless to brownish, rarely with purplish tint (when plants purple-colored), obliquely to erect spreading, not united into fascicles, forming loose mat adjacent to ventral side of stem. Leaves subtransversely inserted, canaliculate to slightly concave–canaliculate, obliquely inserted, shortly or up to 1/3 of stem width decurrent dorsally, subtransversely to arcuately inserted, barely or shortly decurrent ventrally, contiguous, rarely distant or subimbricate and then enclosed with one another, ovate to obliquely ovate, 950–1450 × 1000–1450 µm. Midleaf cells 30–42.5 × 22.5–27.5 µm, oblong, thin-walled, trigones moderate to large in size, convex, cuticle smooth; cells along leaf margin 25–35 µm, thin-walled, trigones moderate in size to large, convex to coarsely bulging, external wall noticeably thickened, cuticle smooth. Dioicous. Androecia intercalary, with 3–7 pairs of bracts. Bracts inflated in lower half and canaliculate above, 1-androus, different generations divided by 5–6 pairs of sterile leaves. Perianth obovate to obovate–fusiform, exerted for 2/3–3/4 of its length, more or less smooth or shallowly 3-plicate in upper 1/4–1/3 of its length, gradually narrowing to non-obscurely beaked mouth, composed of subisodiametric cells; cells in the perianth middle thin-walled, walls brownish to pinkish brownish, 20–33 × 25.0–37.5 µm, trigones moderate in size, triangle to slightly concave or convex, bistriatose in lower 1/2–2/3; perigynium virtually absent; female bracts canaliculate, obliquely spreading or loosely sheathing the perianth. Elaters bispiral, ca. 150 × 7 µm, with long (ca 25 µm) homogenous ends. Spores papillose, brownish to brown, ca. 20 µm in diameter (Figure 54G, Figure 55C–E and Figure 56A–E).

Comment: This species is distinctive due to its large size, ovate canaliculated and obliquely spreading leaves, and large leaf cells.

*Solenostoma rishiriense* Amakawa Journal of Japanese Botany 31: 48. f. 3: a–l. 1956.

Type: Japan, Hokkaido, near summit of Rishiri Island, 1600–1719 m a.s.l., on andesite rocks in exposed conditions (sunny, readily drier); 22 July 1964, D. Shimizu (Holotypus NICH-53527!).

Accepted name: *Solenostoma rishiriense* Amakawa.

Description: Plants prostrate to ascending, brownish to yellowish brown and blackish brown, 0.3–0.6 mm wide (to 1.0 mm wide near perianth) and 3–5 mm long, encrusted by soil particles. Stem 60–150 µm in diameter, sparsely laterally branched, brownish to brown. Rhizoids sparse, colorless to grayish and brownish, separated and erect spreading. Leaves distant to contiguous (in perianthous shoots, mostly contiguous), obliquely spreading, concave–canaliculate to concave, subtransversely inserted, up to 1/2 of stem width decurrent in both sides, 330–750 × 280–830 µm, ovate to obovate and nearly rounded (bigger ones). Midleaf cells subisodiametric to shortly oblong, 16–25 × 12–18 µm, thin-walled, walls brownish, trigones moderate in size, triangle to slightly concave or convex, cuticle smooth; cells along leaf margin 10–17 µm, thin-walled, with rather small, concave trigones, cuticle smooth. Possibly dioicous. Perianth exerted for 2/5–1/2 of its length or hidden within bracts, nearly spherical, ca. 600 × 550 µm, loosely 3-plicate in upper part, loosely beaked, mouth crenulate, composed of oblong, clavate cells with thickened walls, seemingly unistratose to base, composed of nearly isodiametric cells, 13–20 µm in diameter, thin-walled, with moderate-in-size, triangle to slightly concave or convex trigones; perigynium virtually absent; bracts sheathing the perianth or obliquely spreading in upper 1/3 (Figure 55F and Figure 56F–L).

Comment: The species is somewhat morphologically similar to *Solenostoma minutissimum,* from which, however, it differs in a number of features: (1) its hidden or only shortly exerted perianth, versus one exerted for 2/3 or more of the length; (2) the absence of red or purple coloration; and (3) its distribution, which is restricted to North Japan.

## 5. Materials and Methods

As in earlier works, we follow the ‘narrow’ generic concept within Solenostomataceae, which is not yet accepted widely, but in our opinion will be the most useful [32]. As previously argued [32], we consider it necessary to treat *Plectocolea*, *Metasolenostoma* and *Protosolenostoma* as independent genera from *Solenostoma* s.l. In addition, the splitting of these narrow genera may be logically continued via the re-establishment of *Eucalyx* at the generic level (type species *E. obovatus* (C. G. D. Nees) Breidler). The equal importance of *Eucalyx* to other divisions of *Solenostoma* s.l. is also obvious from the phylogenetic trees of Shaw et al. [16], which contain specimens of both Solenostomataceae and related groups. To do this within the framework of this paper, in our opinion, would be premature, since the exact species composition of this entity within *Solenostoma* s.l. is not yet well known (likely, the eight species included in the checklist by Söderström et al. [17] for this subgenus do not encompass all of the taxonomical diversity of the group, and a number of taxa should be transferred to *Eucalyx* from *Plectocolea* and *Solenostoma* s. str.).

All specimens mentioned here were studied using microscopes kindly provided by the curators of the herbaria (acronyms are provided in the nomenclatural citations). Morphological descriptions were compiled at the study site, and no material was loaned from herbaria outside of the holding herbaria. Photographs were taken with a hand-held digital camera or using digital tools provided by the herbaria. Subsequently, photographs of the external appearance were drawn by applying transparent tracing paper to the photographs.

Each studied specimen is annotated as follows:

(1) Basionym, with the nomenclatural citation. All discussed taxa are arranged alphabetically by basionym name;
(2) Type status, label citation and database number (if applicable);
(3) Accepted name in accordance with the accepted taxonomical concept, providing synonyms from Söderström et al. [17], if any;
(4) Morphological description for most of the specimens, to the extent possible due to material quality;
(5) Comments on taxonomy.

Line-art drawings are provided for all taxa. In the vast majority of cases, they are supplemented by microphotographs. Labelled photos are included when it is very difficult to discern the identity of specimens (especially concerning the type specimens in STR that have not yet been barcoded).

In the course of our study, the following herbaria were visited, and their materials were studied: BM (The Natural History Museum, London, UK), G (Conservatoire et Jardin botaniques de la Ville de Genève, Genève, Switzerland), HIRO (Hiroshima University, Hiroshima-ken, Higashi-Hiroshima-shi, Japan), JE (Friedrich Schiller University Jena, Jena, Germany), NICH (Hattori Botanical Laboratory, Miyazaki Prefecture, Nichinan City, Japan), PC (Muséum National d’Histoire Naturelle, Paris, France) and STR (University of Strasbourg, Strasbourg, France). The abbreviation ‘s.n.’ used after the herbarium acronyms means ‘sine numero’ or ‘without number’ in English.

## 6. Conclusions

The taxonomic diversity of *Solenostoma*, *Metasolenostoma*, *Protosolenostoma*, and *Plectocolea* in Pacific Asia is greatly underestimated. The taxonomic composition in the transition zone between the Himalayan, Tibetan and East China floras is only partially represented by the species known due to Amakawa’s studies [5,6,7]. Many taxa need to be described, but first, it is necessary to understand the morphology of already known species based on type specimens, which is partially achieved in the present account. There is still very little information on the genetic structure of *Solenostoma* s.l., and the information available is not sufficient for a comprehensive comparison and description of population characteristics.

## Figures and Tables

**Table 1:** Distinctions between *Solenostoma abyssinicum* and morphologically similar taxa.

*S. abyssinicum*	*S. sphaerocarpum*	*S. confertissimum*
Paroicous + male branches	Paroicous + male branches	Paroicous
Perianth mouth not beaked	Perianth mouth beaked	Perianth mouth beaked
Rhizogenous cells very near base (1–2 rows), occurring upper in male and female bracts only	No rhizogenous cells	Rhizogenous cells in sterile leaves in lower 1/5–1/4 of the leaf length

## Data Availability

Data are contained within the article.

## References

[1] Bakalin V.A. (2014). The study of type collection in Conservatoire et Jardin Botanique de la Ville de Genève (G): The hepatic genera Jungermannia, Solenostoma and Plectocolea. Arctoa.

[2] Váňa J. (1974). Studien über die Jungermannioideae (Hepaticae) 6. Jungermannia Subg. Solenostoma: Europäische und nordamerica nische Arten. Folia Geobot. Phytotaxon..

[3] Váňa J. (1975). Studien über die Jungermannioideae (Hepaticae) 8. *Jungermannia* Subg. *Plectocolea* und Subg. *Solenostoma* in Australien, Neuseeland und Ozeanien. Folia Geobot. Phytotaxon..

[4] Váňa J. (1975). Studien über die Jungermannioideae (Hepaticae) 7. *Jungermannia* Subg. *Plectocolea*: Europäische und nordamerikanische Arten. Folia Geobot. Phytotaxon..

[5] Amakawa T. (1963). New or little known Asiatic species of the family Jungermanniaceae. I. J. Hattori Bot. Lab..

[6] Amakawa T. (1966). New or little known Asiatic species of the family Jungermanniaceae. II. J. Hattori Bot. Lab..

[7] Amakawa T. (1968). New or little known Asiatic species of the family Jungermanniaceae. IV. J. Hattori Bot. Lab..

[8] Amakawa T. (1960). Family Jungermanniaceae of Japan, II. J. Hattori Bot. Lab..

[9] Gao C., Bai X.-L. (2001). A synoptic revision of family *Jungermanniaceae* (Hepaticae) in China including some taxa nova. Philipp. Sci..

[10] Bakalin V.A. (2014). The revision of ‘*Jungermannia* s.l.’ in the North Pacific: The genera *Endogemma*, *Jungermannia* s. str., *Metasolenostoma*, *Plectocolea* and *Solenostoma* (Hepaticae). Bot. Pacifica.

[11] Bakalin V.A., Choi S.S., Park S.J., Sim S.H., Hyun C.W. (2020). A taxonomic revision of *Solenostomataceae* (Marchantiophyta) in Korea. Korean J. Plant Taxon..

[12] Bakalin V.A., Klimova K.G., Nguyen V.S., Bakalin D.A., Choi S.S. (2021). Liverwort oil body diversity in Pacific Asia. Arctoa.

[13] Váňa J. (1974). Miscellaneous notes on the Asiatic Jungermannioideae III. J. Hattori Bot. Lab..

[14] Váňa J., Inoue H. (1983). Studies on Taiwan Hepaticae. V. Jungermanniaceae. Bull. Nat. Sci. Mus. Ser. B.

[15] Gottsche C.M., Lindenberg J.B.W., Nees C.G. (1844). Synopsis Hepaticarum: Coniunctis Studiis Scripserunt et Edi Curaverunt.

[16] Shaw B., Crandall-Stotler B., Váňa J., Stotler R.E., von Konrat M., Engel J.J., Davis C.E., Long D.G., Sova P., Shaw A.J. (2015). Phylogenetic relationships and morphological evolution in a major clade of leafy liverworts (phylum Marchantiophyta, order *Jungermanniales*): Suborder *Jungermanniineae*. Syst. Bot..

[17] Söderström L., Hagborg A., von Konrat M., Bartholomew-Began S., Bell D., Briscoe L., Brown E., Cargill D.C., Costa D.P., Crandall-Stotler B.J. (2016). World checklist of hornworts and liverworts. PhytoKeys.

[18] Inoue H., Kurokawa S. (1979). Three new hepatics from Papua New Guinea. Studies on Cryptogams of Papua New Guinea.

[19] Nees C.G. (1830). Enumeratio Plantarum Cryptogamicarum Javae et Insularum Adiacentium. Fasciculus Prior, Hepaticas Complectens, ab Editore Illustratas.

[20] Nees C.G., Montagne J.F.C. (1836). *Jungermanniearum* herbarii Montagneani species. Ann. Des Sci. Nat. Bot..

[21] Váňa J. (1972). Miscellaneous notes on the Asiatic *Jungermannioideae* II. J. Hattori Bot. Lab..

[22] Hattori S., Hara H. (1966). Anthocerotae and Hepaticae. The Flora of Eastern Himalaya: Results of the Botanical Expedition to Eastern Himalaya Organized by the University of Tokyo 1960 and 1963.

[23] Váňa J. (1972). Miscellaneous notes on the Asiatic *Jungermannioideae*. J. Hattori Bot. Lab..

[24] Bakalin V.A., Vilnet A.A. (2012). New combinations and new species of *Solenostoma* and *Plectocolea* (Solenostomataceae) from the Russian Far East. Bryologist.

[25] Hattori S., Ohashi H. (1975). Bryophyta, Anthocerotae & Hepaticae. Flora of Eastern Himalaya.

[26] Váňa J., Long D.G. (2009). Jungermanniaceae of the Sino-Himalayan region. Nova Hedwig..

[27] Amakawa T. (1957). Notes on Japanese Hepaticae (5). J. Jpn. Bot..

[28] Amakawa T. (1957). Notes on Japanese Hepaticae (6). J. Jpn. Bot..

[29] Hattori S. (1948). Hepaticarum species novae vel minus cognitae Nipponenses. VI. J. Hattori Bot. Lab..

[30] Herzog T., Noguchi A. (1955). Beitrag zur Kenntnis der Bryophytenflora von Formosa und den benachbarten Inseln Botel Tobago und Kwashyoto. J. Hattori Bot. Lab..

[31] Amakawa T. (1956). Notes on Japanese Hepaticae (2). J. Jpn. Bot..

[32] Bakalin V.A., Vilnet A.V., Furuki T., Katagiri T. (2014). Taxonomic novelties in Solenostoma-Plectocolea complex (Solenostomataceae, Hepaticae) in East Asia. Bot. Pacifica.

